# Transcriptional kinetics and molecular functions of long noncoding RNAs

**DOI:** 10.1038/s41588-022-01014-1

**Published:** 2022-03-03

**Authors:** Per Johnsson, Christoph Ziegenhain, Leonard Hartmanis, Gert-Jan Hendriks, Michael Hagemann-Jensen, Björn Reinius, Rickard Sandberg

**Affiliations:** 1 Department of Cell and Molecular Biology, Karolinska Institutet, Stockholm, Sweden; 2 Department of Medical Biochemistry and Biophysics, Karolinska Institutet, Stockholm, Sweden

**Keywords:** Gene expression profiling, Gene regulation, Genomics

## Abstract

An increasing number of long noncoding RNAs (lncRNAs) have experimentally confirmed functions, yet little is known about their transcriptional dynamics and it is challenging to determine their regulatory effects. Here, we used allele-sensitive single-cell RNA sequencing to demonstrate that, compared to messenger RNAs, lncRNAs have twice as long duration between two transcriptional bursts. Additionally, we observed increased cell-to-cell variability in lncRNA expression due to lower frequency bursting producing larger numbers of RNA molecules. Exploiting heterogeneity in asynchronously growing cells, we identified and experimentally validated lncRNAs with cell state-specific functions involved in cell cycle progression and apoptosis. Finally, we identified *cis*-functioning lncRNAs and showed that knockdown of these lncRNAs modulated the nearby protein-coding gene’s transcriptional burst frequency or size. In summary, we identified distinct transcriptional regulation of lncRNAs and demonstrated a role for lncRNAs in the regulation of mRNA transcriptional bursting.

## Abstract (web summary)

Allele-sensitive single-cell RNA sequencing analysis of long noncoding RNA (lncRNA) transcriptional kinetics shows that their lower expression compared to mRNA is due to lower burst frequencies and highlights cell-state-specific functions for several lncRNAs.

## Main

Mammalian genomes encode thousands of lncRNAs^1,2^ but identifying their molecular functions has proven difficult. Functional predictions based on primary sequence, evolutionary conservation^3^ or genomic location are often unreliable; to date we still cannot identify active lncRNAs and their mechanism of action without extensive experimentation. Consequently, the functions of most lncRNAs are unknown^4^ and new experimental and computational approaches are needed to efficiently identify lncRNAs for in-depth functional validation and characterization.

Transcription of mammalian genes typically occurs in short bursts of activity^5^. Through recent methodological^6^ and computational^7^ developments, it is now feasible to infer burst parameters for thousands of genes simultaneously. lncRNAs are typically expressed at lower levels than mRNAs^2,8–12^ and many at average levels below one RNA copy per cell^13^. Therefore, it has been proposed that averaging transcriptomes over thousands of cells masks the presence of rare cells with high lncRNAs expression^14^. However, analyses of transcriptional bursting to date have focused on protein-coding genes and it is unknown whether the low expression of lncRNAs is mediated by lowered burst sizes (fewer RNA molecules per cell) or burst frequencies (expression in fewer cells). Moreover, comprehensive analyses of transcriptional dynamics and cell-to-cell variability of lncRNAs are still missing and most studies to date were limited to low throughput methods measuring limited numbers of genes and cells^15^.

The introduction of single-cell RNA sequencing (scRNA-seq) technologies^16^ and protocols for allele-specific quantification^17^ offers new opportunities to characterize transcriptional dynamics and allele-specific gene expression in individual cells for thousands of genes simultaneously. In this study, we introduce allele-sensitive scRNA-seq of lncRNAs to investigate lncRNA transcriptional bursting kinetics and identify lncRNA candidates with roles in cellular processes and transcriptional regulation.

## Results

### Detection of lncRNAs and mRNAs in individual cells

**Fig. 1:**
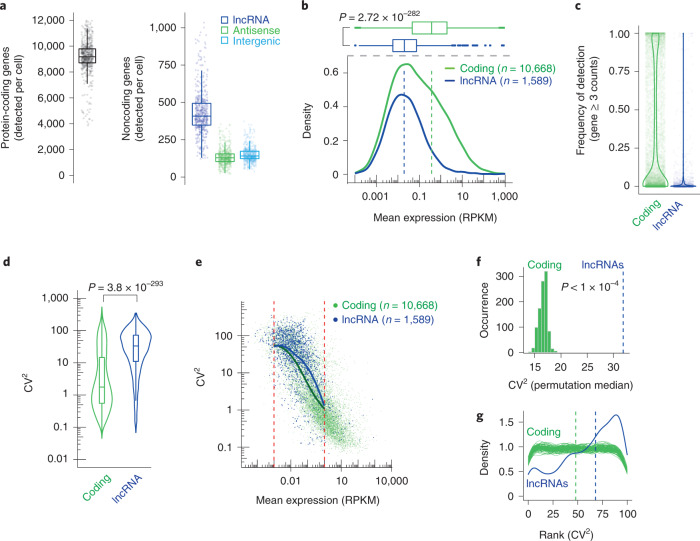
Levels and variability of lncRNA and mRNA expression. **a**, Boxplots showing the detected numbers of protein-coding genes (left) and subtypes of lncRNAs per fibroblast (right), based on Smart-seq2 data (*n* = 533 cells), requiring 3 or more read counts for detection. **b**, Densities and boxplots of mean expression levels for lncRNAs and mRNAs across fibroblasts (*n* = 533). The dashed lines denote the medians, the *P* value represents a two-sided Wilcoxon test. **c**, Violin plots showing the fraction of cells that detected individual lncRNAs and mRNAs (requiring three or more read counts for detection). **d**, Violin plots showing the CV^2^ for lncRNAs and mRNAs expression across fibroblasts (*n* = 533). The *P* value represents a two-sided Wilcoxon test**. e**, Scatterplot of mean expression against the CV^2^ for lncRNAs (blue) and mRNAs (green). The lines denote a smoothed fit to the rolling mean (width = 15) for lncRNAs and mRNAs. The red dotted lines denote the expression range for the smoothed fit. **f**, Histogram showing the distribution of median CV^2^ for sampled expression-matched sets of mRNAs. The *P* value represents the outcome of the permutation test (*n* = 10,000) where the CV^2^_mRNA_ (median) was higher than the observed CV^2^_lncRNA_ (median, blue dashed line). **g**, Densities of rankings of CV^2^ for lncRNAs (*n* = 1,519) and subsampled mRNAs. Blue, Ranking of CV^2^ (lncRNAs) to 100 expression-matched mRNAs (frequency CV^2^_lncRNA_ > CV^2^_mRNA_matched_). Green, the ranking of CV^2^ to subsampled mRNAs (*n* = 1,519 mRNAs, as many as lncRNAs) to 100 expression-matched mRNAs (frequency CV^2^_mRNA_random_ > CV^2^_mRNA_matched_, subsampling repeated 100 times). The dashed lines denote the medians of ranking for lncRNAs and mRNAs. **a**,**b**,**d**, The center lines show the medians, the interquartile limits indicate the 25th and 75th percentiles, the whiskers denote the farthest points at a maximum of 1.5 times the interquartile range (IQR). The analysis in Fig. 1d–g represents easily separated transcriptional units of lncRNAs and mRNAs (Extended Data Fig. 1e).

**Extended Data Fig. 1:**
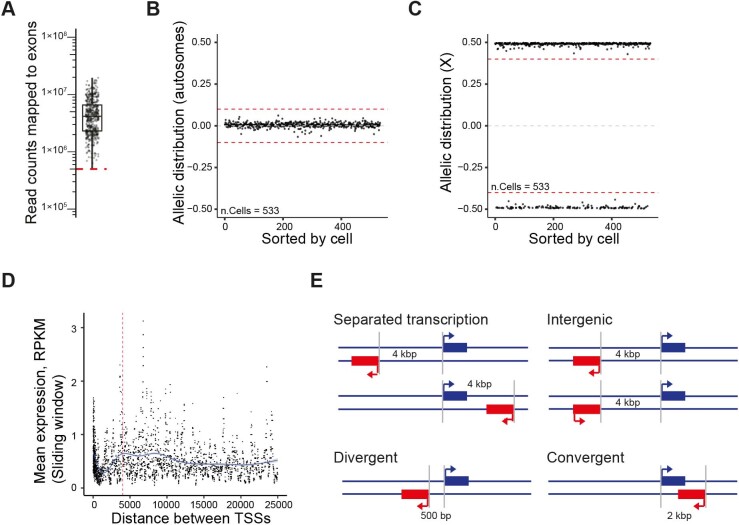
Quality controls for allele-sensitive scRNA-seq data. **(A)** Boxplot showing number of sequenced read counts mapped to genes (Smart-seq2 libraries, n = 533 cells). The center line shows the median, interquartile limits indicate the 25^th^ and 75^th^ percentiles and whiskers denote the farthest points at a maximum of 1.5 times the interquartile range. The red line represents quality control cutoff. **(B)** Scatter plot showing the distribution of allele sensitive read counts ((counts_CAST_ / (counts_CAST_ + counts_C57_) − 0.5)) for non-imprinted autosomal genes (red dashed lines represent quality control cutoffs). **(C)** Scatter plot showing the distribution of allele sensitive read counts for non-escapee genes on the X-chromosome (red dashed lines represent quality control cutoff). **(D)** Mean expression against the distance between two promoters. The blue line denotes a generalized additive mode smoothing (gam) fitted to the sliding median (median RPKM, Width=51). The red dashed line represents the cutoff in distance between two promoters for being considered as separated transcriptional unit (4 kb). **(E)** Schematic representation of organizations of genes and the nomenclature used in this manuscript.

We first investigated the expression patterns of lncRNAs and mRNAs in 533 individual primary adult tail fibroblasts derived from the cross between the distantly related CAST/EiJ and C57BL/6J mouse strains (5 animals). Single-cell transcriptomes were created with Smart-seq2 (ref. ^18^) to leverage that method’s high sensitivity^19^ and full gene body coverage, enabling allele-level RNA profiling for more than 80% of all genes^17^. We verified that non-imprinted autosomal genes had similar overall expression from the CAST and C57 alleles and that our allelic expression levels accurately detected monoallelic expression for X chromosome genes^20^ (Extended Data Fig. 1a–c and Supplementary Table 1). A total of 24,653 genes were detected, including 15,869 mRNAs and 3,311 noncoding RNAs (Supplementary Table 2). The detection of hundreds of lncRNAs per cell (median 9,173 protein-coding mRNAs and 408 lncRNAs per cell; Fig. 1a) motivated us to proceed with in-depth investigations of lncRNA expression across cells. We initially excluded lncRNAs and mRNAs that had another promoter within 4 kilobases (kb) since we noticed that genes with closely located promoters had increased expression (Extended Data Fig. 1d,e, referred to as easily separated transcriptional units).

### lncRNAs are expressed with higher cell-to-cell variability

**Extended Data Fig. 2:**
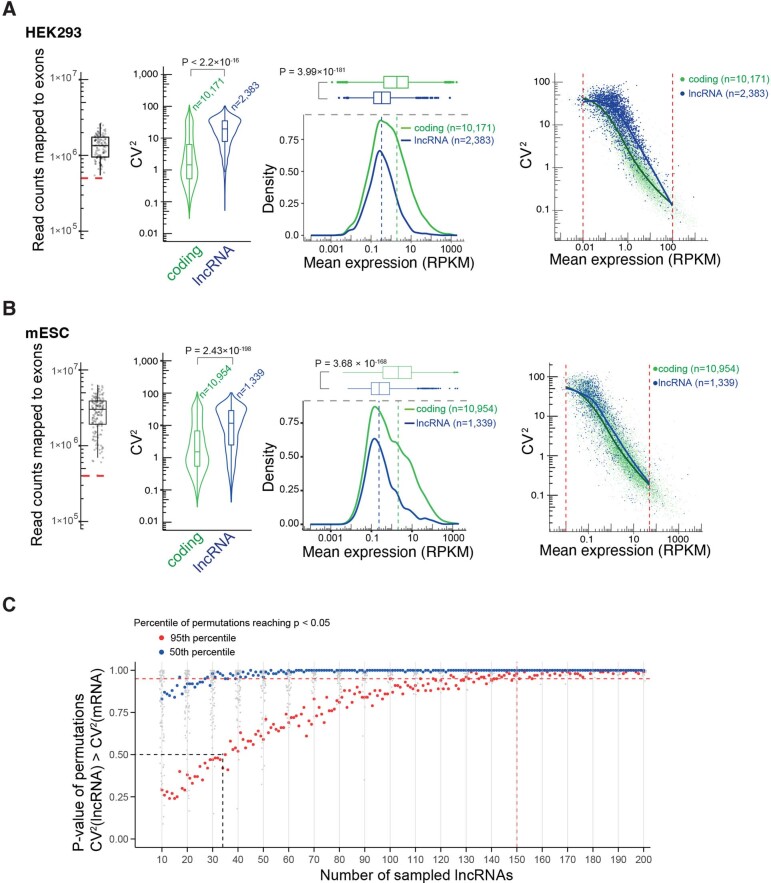
Analyses of lncRNA expression variability in HEK293 and mES cells. **(A-B)** Reproducing the analyses and results presented in Fig. 1A, B, D, E usingHEK293 **(A)** cell or mouse embryonic stem cell **(B)** data (p-values represent a two-sided Wilcoxon test). The center lines of the boxplots show the medians, interquartile limits indicate the 25^th^ and 75^th^ percentiles, whiskers denote the farthest points at a maximum of 1.5 times the interquartile range). **(C)** Scatter plot showing the number of lncRNAs required to identify increased CV^2^ compared to expression-matched mRNAs. The x-axis represents the number of lncRNAs used for CV^2^ quantification. For each number of lncRNAs analyzed, expression-matched mRNAs were randomly selected followed by subsampling one expression-matched mRNA for each lncRNA (1,000 permutations, similar as in Fig. 1F,). The y-axis represents the outcome of the permutation test (median(CV^2^_lncRNA_) > median(CV^2^_mRNA_)). Grey points represent individual p-values for the permutation test. Blue points represent the 50^th^ percentile of permutations reaching significance. Red points represent the 95^th^ percentile of permutations reaching significance. The black dashed line represents subsampling 34 lncRNAs.

We first investigated the expression patterns of lncRNAs and mRNAs; as expected^2,21^, lncRNAs were expressed at lower levels than mRNAs (Fig. 1b and Supplementary Note) and detected in fewer cells (median 3 and 31% of cells, respectively) (Fig. 1c). To investigate if lncRNA expression is more variable between cells, we computed the squared coefficient of variation (CV^2^) and observed significantly higher variability for lncRNAs (Fig. 1d). Contrasting CV^2^ against the mean expression revealed that lncRNAs had higher CV^2^ than mRNAs across a wide range of expression levels (Fig. 1e). To systematically account for possible confounding differences in mean expression of lncRNAs and mRNAs, we generated thousands of randomly drawn sets of mRNAs with expressions matched to lncRNAs (Fig. 1f) and ranked the CV^2^ of each lncRNA against 100 expression-matched mRNAs (Fig. 1g; Methods). Consistently, lncRNAs had significantly higher expression variability than expression-matched mRNAs (Fig. 1f,g); this observation was validated in human HEK293 and mouse embryonic stem cells (Extended Data Fig. 2a,b). The ability to detect the increased cell-to-cell variability was dependent on the number of lncRNAs analyzed; when subsampling lncRNAs (and their expression-matched mRNAs) the difference declined and eventually disappeared (Extended Data Fig. 2c).

### Low expression of lncRNAs results from longer burst duration

**Fig. 2:**
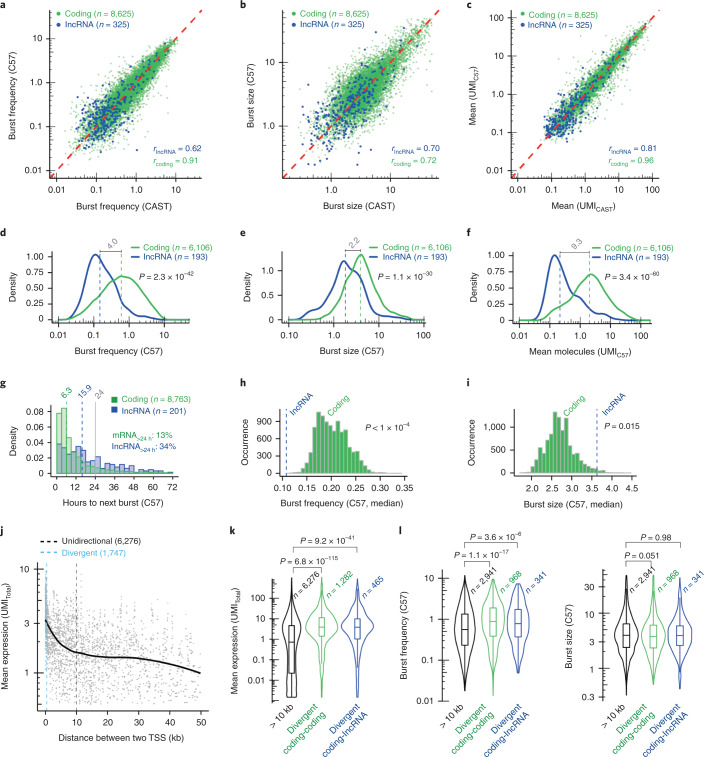
Transcriptional burst kinetics of lncRNAs and divergent promoters. **a**–**c**, Scatterplots of burst frequencies (**a**), burst sizes (**b**) and mean expression (**c**) for mRNAs and lncRNAs comparing the parameters inferred from the CAST allele against the C57 allele for non-imprinted autosomal genes (the red line denotes *x* = *y*, *r* represents the Spearman correlation). **d**–**f**, Density plots for burst frequencies (**d**) burst sizes (**e**) and mean expression (allele-distributed UMIs) (**f**) for mRNAs and lncRNAs (showing the C57 allele). The dashed lines represent the median burst frequencies, sizes and mean expression for mRNAs (green) and lncRNAs (blue). The relative fold changes (median) are annotated in gray. *P* values represent a two-sided Wilcoxon test. **g**, Histogram showing the duration between two bursts from the same allele for mRNAs and lncRNAs. The dashed lines represent the median duration between two bursts for mRNAs (green) and lncRNAs (blue). The gray line represents a duration of 24 h between two bursts. **h**,**i**, Histograms showing the distribution of median burst frequencies (**h**) and burst sizes (**i**) for sampled expression-matched sets of mRNAs with 50 lncRNAs (identified in Extended Data Fig. 4g). The *P* value represents the outcome of the permutation test (*n* = 10,000), where the observed burst parameters (lncRNAs, median) was higher (for burst frequencies) or lower (for burst sizes) than the burst parameters for sampled mRNAs (median). **j**, Scatterplot showing the distance between the TSS of pairs of genes against their mean expression levels (UMIs). The black solid line represents a locally estimated scatterplot smoothing fit to the rolling median (width = 31). The dashed lines represent the distance between two TSS for being assigned as divergent promoters (blue, maximum distance of 500 bp) or unidirectional promoters (black, minimum distance of 10 kbp). **k**, Violin plots showing the mean expression levels of unidirectional mRNAs and for mRNAs transcribed from divergent promoters (either with another mRNA or an lncRNA). *P* values represent a two-sided Wilcoxon test. Fold change in medians: coding-coding: 5.44; coding-lncRNA: 5.37. **l**, Violin plots for unidirectional and divergent promoters representing burst frequencies and burst sizes for the C57 allele. *P* values represent a two-sided Wilcoxon test. **k**,**l**, The center lines represent the medians, the interquartile limits indicate the 25th and 75th percentiles and the whiskers denote the farthest points at a maximum of 1.5 times the IQR.

**Extended Data Fig. 3:**
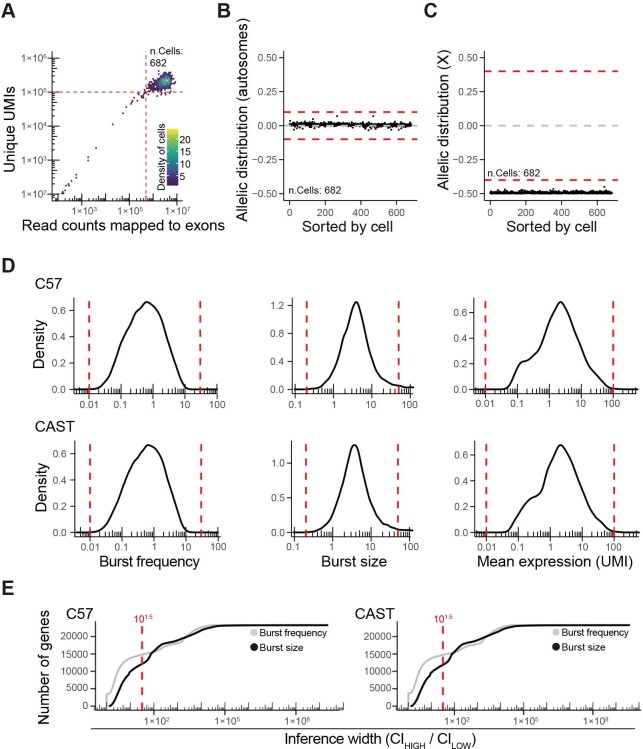
Quality controls of bursting inference data. **(A)** Scatter plot showing the number of sequenced read counts mapped to exons (Smart-seq3 libraries, n = 682 cells passing quality control) (red lines represent quality control cutoffs). **(B)** Scatter plot showing the distribution of allele sensitive read counts for non-imprinted autosomal genes (red dashed lines represent quality control cutoffs). **(C)** Scatter plot showing the distribution of allele sensitive read counts for non-escapee genes on the X-chromosome (red dashed lines represent quality control cutoff). **(D)** Density plots for burst frequencies, burst sizes and mean expression (for allele distributed UMIs). Red dashed lines represent cutoffs for passing the quality control. **(E)** Scatter plots representing widths of confidence intervals (x-axis) against genes (y-axis, sorted by widths of confidence intervals) for burst frequencies and burst sizes. Red dashed lines represent cutoffs for passing the quality control.

**Extended Data Fig. 4:**
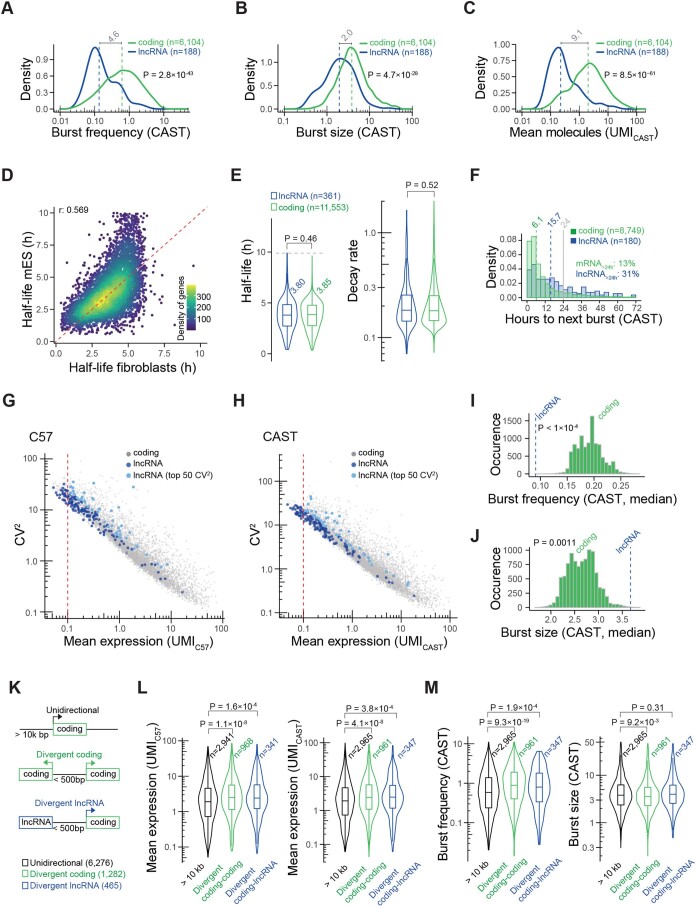
Transcriptional burst kinetics of lncRNAs and divergent promoters. **(A-C)** Density plots for burst frequencies **(A)** burst sizes **(B)** and mean expression (for allele-distributed UMIs) **(C)** for mRNAs and lncRNAs (showing the CAST allele). Dashed lines represent the median burst frequencies, sizes and mean expression for mRNAs and lncRNAs. The relative fold changes are annotated in grey. P-values represent a two-sided Wilcoxon test. **(D)** Scatter plot comparing estimated RNA half-lives for mouse ES cells (y-axis) and mouse fibroblast cells (x-axis). R represents the Spearman correlation. **(E)** Violin plots for RNA half-lives (left) and RNA decay rates (right) for mouse fibroblasts. P-values represent a two-sided Wilcoxon test. **(F)** Histogram showing the duration between two bursts from the same allele (CAST), for mRNAs and lncRNAs. Dashed lines represent the median duration between two bursts for mRNAs and lncRNAs. Grey line represents a duration of 24 hours between two bursts. **(G,H)** Scatter plot of mean expression (for allele-distributed UMIs) against the CV^2^ for lncRNAs and mRNAs for the C57 **(G)** and CAST **(H)** genomes. The top 50 ranked lncRNAs are marked in light blue (see Methods). The red dotted line denotes the lower expression cutoff for lncRNAs to be included in the analysis. **(I,J)** Histograms showing the distribution of median burst frequencies **(I)** and burst sizes **(J)** for sampled expression-matched sets of mRNAs (for lncRNAs identified in (H)). The p-values represent the outcome of the permutation test (n = 10,000, see Methods), **(K)** Schematic representation of divergent and unidirectional transcribing promoters. **(L)** Violin plots for mean expression of mRNAs (allele-distributed UMIs) for divergent and unidirectional promoters for the C57 and CAST alleles. The p-values represent a two-sided Wilcoxon test. Fold-change in medians: C57_coding-coding_ = 1.31; C57_coding-lncRNA_ = 1.27; CAST_coding-coding_ = 1.27 and CAST_coding-lncRNA_ = 1.29. **(M)** Violin plots for unidirectional and divergent promoters representing burst frequencies and burst sizes, for the CAST allele. P-values represent a two-sided Wilcoxon test. **L,M,** The center lines represent the medians, the interquartile limits indicate the 25th and 75th percentiles and the whiskers denote the farthest points at a maximum of 1.5 times the IQR.

We next studied whether the lowered expression level of lncRNAs is due to intrinsic differences in transcriptional bursting kinetics when compared to protein-coding genes. To this end, we generated a comprehensive dataset of 682 cells (postquality control, median 3 × 10^6^ PE100 reads mapped to exons per cell; Extended Data Fig. 3a–e) of adult tail fibroblasts using Smart-seq3 (ref. ^6^) since the unique molecular identifiers (UMIs) are important for accurate burst size inference (Supplementary Note)^7^. After quality control, bursting kinetic parameters were inferred for 10,121 coding genes and 626 lncRNAs on at least 1 of the alleles (8,625 coding and 325 lncRNAs genes on both alleles). Reassuringly, burst parameters and expression levels correlated well between the CAST and C57 alleles for both coding and noncoding genes (Fig. 2a–c). Focusing the analysis on separated transcriptional units (Extended Data Fig. 1e), we found that lncRNAs have a fourfold lower burst frequency compared to mRNAs (Fig. 2d and Extended Data Fig. 4a), and only a twofold decrease in burst size (Fig. 2e and Extended Data Fig. 4b). Thus, the decreased expression of lncRNAs (Fig. 2f and Extended Data Fig. 4c) was mainly achieved through longer duration between transcriptional bursts of expression.

Since the inferred parameters for burst frequencies were on the timescale of RNA degradation^7^, we next generated RNA decay rates in primary fibroblasts to derive burst frequencies on absolute timescales (using actinomycin D to inhibit transcription; Methods). The estimates were in agreement with previous measurements (Extended Data Fig. 4d)^22^, with an average half-life slightly below 4 h, with, as expected^23^, similar decay rates for mRNAs and lncRNAs (Extended Data Fig. 4e). The decay rates were used to transform burst frequencies into hours, which interestingly revealed that the duration between two subsequent lncRNA bursts (from the same allele) were more than twice as long compared to mRNAs (15.9 and 6.9 h, respectively, median) (Fig. 2g and Extended Data Fig. 4f). Notably, over 30% of lncRNAs were found to burst less than once every 24 h on each individual allele.

We next explored if the increased cell-to-cell variability of lncRNAs compared to expression-matched mRNAs (Fig. 1e-g and Extended Data Fig. 2a,b) was related to alterations in bursting parameters. Focusing on the top 50 most variable lncRNAs from each allele (ranked CV^2^; Extended Data Fig. 4g,h and Methods), we observed that lncRNAs had decreased burst frequencies (Fig. 2h and Extended Data Fig. 4i) and increased burst sizes (Fig. 2i and Extended Data Fig. 4j) compared to expression-matched mRNAs. These data suggest more sporadic expression of lncRNAs (due to lowered burst frequency), although with increased numbers of RNA molecules produced per burst (due to increased burst size), and link lncRNAs with the highest cell-to-cell variability to a shift in transcriptional burst kinetics.

Many lncRNAs are transcribed in the antisense direction of protein-coding (sense) genes^24^ and we next investigated if such genomic organizations could result in altered transcriptional kinetics. We identified loci with divergent (in this article referred to the presence of a stable annotated transcript in both sense and antisense direction) mRNA-mRNA pairs, divergent mRNA-lncRNA pairs and unidirectional mRNA-transcribed promoters (Extended Data Fig. 4k). In line with previous studies^8,25^, we identified increased expression of divergently transcribing promoters (Fig. 2j,k and Extended Data Fig. 4l), for mRNA-mRNA and mRNA-lncRNA promoters, compared to unidirectional transcribing promoters (approximately fivefold increase; Fig. 2k). We identified an increase in burst frequency for divergently mRNA-mRNA- and lncRNA-mRNA-transcribing promoters, with no consistent increase in burst size (Fig. 2l and Extended Data Fig. 4m).

### Transient cell cycle states reveal lncRNA functions

**Fig. 3:**
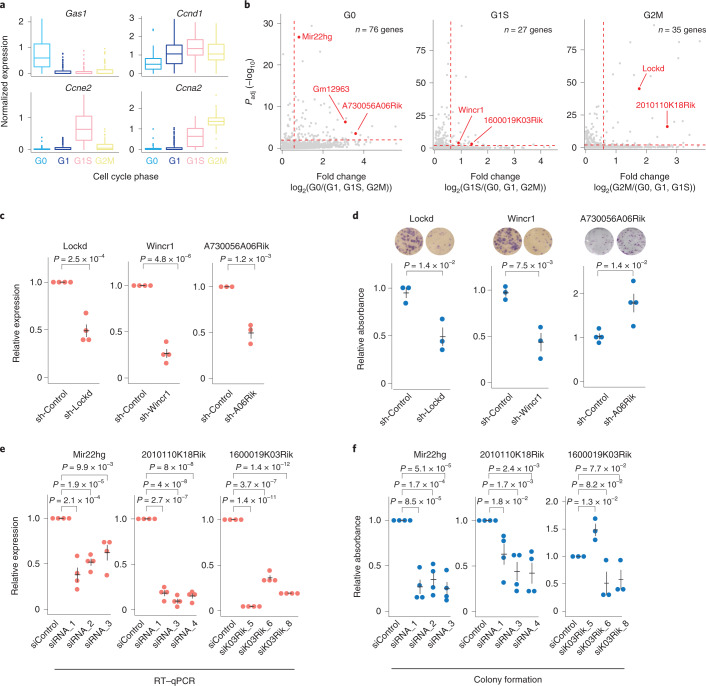
Identification of cell cycle regulated lncRNAs using scRNA-seq. **a**, Boxplots showing the normalized expression levels of cell cycle marker genes in cells classified to the cell cycle phase (*n* = 533 cells, the center lines show the medians, the interquartile limits indicate the 25th and 75th percentiles and the whiskers denote the farthest points at a maximum of 1.5 times the IQR, colored according to the cell cycle phase). **b**, Scatterplots showing lncRNAs with significant expression differences across cell cycle phases (*y* axis, Benjamini–Hochberg-adjusted ANOVA) against the fold induction (*x* axis) compared to the other cell cycle phases. The top ranked candidates selected for further validation are colored red. **c**, Relative expression levels of candidate lncRNAs in lentiviral transduced NIH/3T3 cells measured by RT–qPCR. **d**, Quantification of colony-forming cells in shControl cells and cells with stable shRNA-induced knockdown of lncRNAs, together with representative photos of staining (whole-well images). **e**, Relative expression of cell cycle-associated lncRNAs on siRNA-induced knockdown, measured by RT–qPCR. **f**, Quantification of colony-forming cells on siRNA-induced knockdown for candidate lncRNAs. **c**–**f**, *n* = 3–4 biologically independent samples, data presented as mean values ± s.e.m. and the *P* values represent a two-sided Student’s *t*-test.

**Extended Data Fig. 5:**
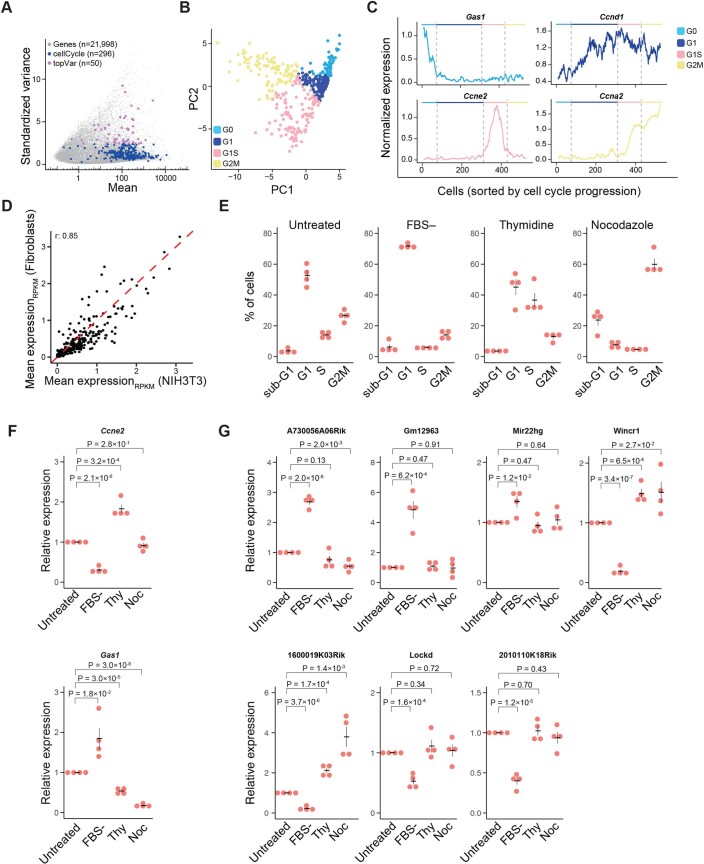
Identification of cell cycle associated lncRNAs. **(A)** Scatter plot highlighting the 50 most variable cell cycle genes on top of a scatter plot showing mean and standardized variance in expression over cells. **(B)** Low dimensional PCA projections of cells based on the most variable genes identified in (A). Cells are colored according to the annotated cell cycle phase. **(C)** Expression pattern of representative marker genes across cells, ordered according to cell cycle progression. The expression represents a sliding window (width = 15) of the mean expression. Colors represent marker genes for the individual cell cycle phases. **(D)** Scatter plot representing the mean expression of cell cycle genes in mouse primary fibroblasts (n = 533 cells) against lentiviral transduced (shRNA-Control) NIH/3T3 (n = 147 cells). R represents the Spearman correlation. **(E)** Cell cycle distribution of NIH/3T3 cells upon various cell cycle synchronizations by indicated compounds. **(F)** Relative expression measured by RT-qPCR of two cell cycle marker genes upon cell cycle synchronization in NIH/3T3 cells. Samples were standardized to DNase treated RNA input. **(G)** Relative expression measured by RT-qPCR for candidate lncRNAs upon cell cycle synchronization in NIH/3T3 cells. Samples were standardized to DNast treated RNA input. (**E-G**; n = 4 biologically independent samples, data presented as mean values + /- s.e.m., p-values represent a two-sided Student’s t-test).

We hypothesized that variable lncRNA expression across transient cellular states carries information as to their function (guilt by association^26^); we first evaluated this strategy on lncRNA expression during the cell cycle. Single-cell transcriptomes from asynchronously grown mouse fibroblasts (*n* = 533; Extended Data Fig. 1a–c) were projected into low-dimensional principal component analysis (PCA) space using the most variable^27^ cell cycle genes^28^ (Extended Data Fig. 5a,b and Supplementary Table 3), clustered; the PCA coordinates were used to fit a principal curve^29^. Cells were aligned onto the cell cycle progression curve and we confirmed the relative expression of a subset of well-established cell cycle genes expressed specifically in G0, G1, G1/S or G2/M (Fig. 3a and Extended Data Fig. 5c). We identified 128 lncRNAs with significant cell cycle-specific expression patterns (Fig. 3b and Supplementary Table 4; analysis of variance (ANOVA) test, false discovery rate (FDR) < 0.01, Benjamini–Hochberg-adjusted). For the validation experiments, we selected at least two highly ranked candidate lncRNAs from each cell cycle phase (based on adjusted *P* values and fold change inductions), excluded lncRNAs that overlapped with multiple other genes to facilitate downstream perturbation experiments and proceeded with seven lncRNA candidates for further characterization (marked in Fig. 3c).

To evaluate potential lncRNA functions in cell cycle progression, we used the immortalized mouse embryonic NIH/3T3 fibroblasts, which express similar cell cycle genes^28^ as primary fibroblasts (Supplementary Table 3) and also correlate well in expression levels (Extended Data Fig. 5d). Next, the cell cycle progression of NIH/3T3 cells was synchronized by serum starvation (G0/G1), thymidine block (G1/S) or nocodazole treatment (G2/M) and validated by flow cytometry (Extended Data Fig. 5e) and quantitative PCR with reverse transcription (RT–qPCR) for two cell cycle marker genes (Extended Data Fig. 5f and Supplementary Table 5a). All seven lncRNAs had the predicted cell cycle expression pattern as measured by RT–qPCR (Extended Data Fig. 5g). Having validated the cell cycle-specific expression of the selected lncRNAs, we next generated individual lentiviral transduced NIH/3T3 cell lines with stable short hairpin RNA (shRNA)-induced knockdown for three of the candidates (Wincr1, Lockd and A730056A06Rik, representing candidates from each cell cycle phase) to perform an in-depth functional investigation (Fig. 3c). Notably, significant effects were observed in the colony formation assays (Fig. 3d), which provide a moderate stress on cells. While the knockdown of A730056A06Rik (expressed on serum starvation; Extended Data Fig. 5g) resulted in the formation of more colonies, the knockdown of Wincr1 and Lockd (expressed in proliferating cells; Extended Data Fig. 5g) reduced the numbers of colonies formed (Fig. 3d). To evaluate our approach more broadly, three additional candidate lncRNAs (Mir22hg, 2010110K18Rik, 1600019K03Rik) were targeted by small interfering RNAs (siRNA) (Fig. 3e) and the effect measured in colony formation assays. Two of three lncRNAs (Mir22hg, 2010110K18Rik) had a consistent effect with fewer colony-forming cells for multiple evaluated siRNAs, while knockdown of 1600019K03Rik was inconsistent between the three evaluated siRNAs (Fig. 3f). Together, this showed that lncRNA expression through cellular states can be efficiently utilized to predict their cellular phenotypes.

### Functional investigation of the lncRNA Lockd

**Fig. 4:**
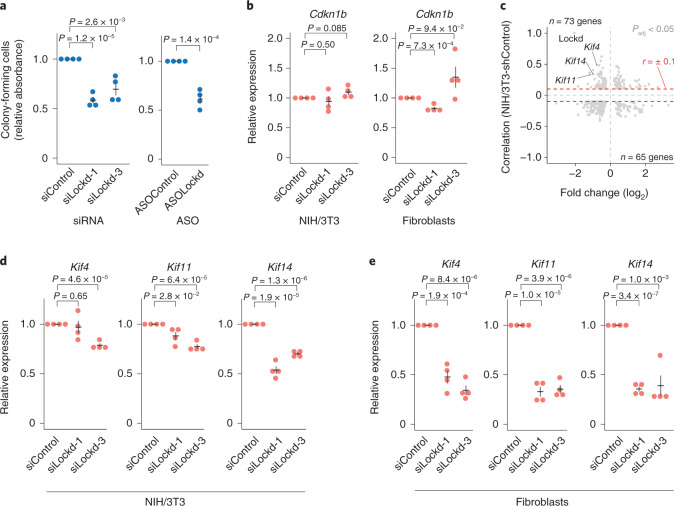
Functional analysis of the lncRNA Lockd. **a**, Quantification of colony-forming cells on siRNA- and ASO-induced knockdown of Lockd in NIH/3T3 cells. **b**, Relative expression of *Cdkn1b* on siRNA-induced knockdown of Lockd in NIH/3T3 cells (left) and primary fibroblasts (right) measured by RT–qPCR. **c**, Scatterplot representing the magnitudes of fold changes of gene expression (shLockd/shControl) for significant genes (SCDE *P* < 0.05, two-sided test using the multiple testing-corrected (Holm procedure) z-score) of stably transduced NIH/3T3 cells (*x* axis) against gene correlations to Lockd in stably shControl-transduced NIH/3T3 cells (*y* axis). Genes reaching the threshold of Spearman correlations (±0.1, denoted with red dashed lines) were considered for downstream analysis. **d**, Relative expression of candidate genes on siRNA-induced knockdown of Lockd in NIH/3T3 cells measured by RT–qPCR. **e**, Relative expression of candidate genes on siRNA-induced knockdown of Lockd in primary fibroblast cells measured by RT–qPCR. **a**,**b**,**d**,**e**, *n* = 4 biologically independent samples, data presented as mean values ± s.e.m, *P* values represent a two-sided Student’s *t*-test.

**Extended Data Fig. 6:**
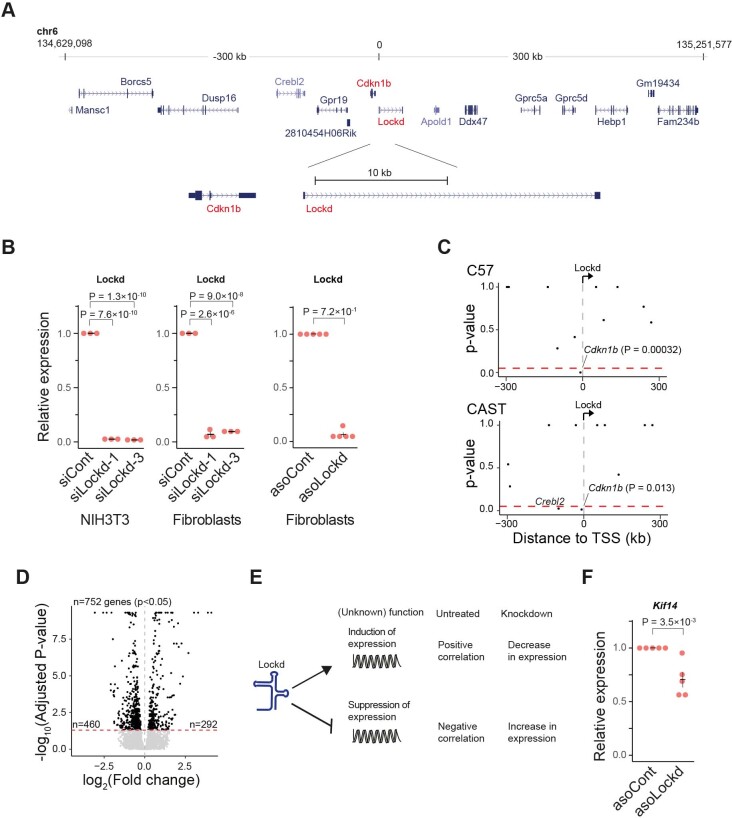
Analysis of the Lockd harboring genomic loci. **(A)** A schematic from the UCSC genome browser representing the *Cdkn1b-Lockd* gene locus. **(B)** Relative expression of Lockd upon; siRNA induced knockdown in NIH/3T3 cells (n = 3 biologically independent samples), primary fibroblasts (n = 3 biologically independent samples) and ASO induced knockdown in primary fibroblasts (n = 5 biologically independent samples) measured by RT-qPCR. **(C)** Scatter plot showing p-values for co-expression of Lockd against genes in proximity for the CAST and C57 genomes (Fisher’s exact test, ± 300 kb of TSS-Lockd, genes filtered for > = 3 allelic counts). The red dashed lines denote threshold for significance (p < 0.05). **(D)** Scatter plot showing the magnitude (x-axis, SCDE maximum likelihood estimate of the fold change) against significance levels (y-axis, SCDE p-value, two-sided test using the multiple testing corrected (Holm procedure) z-score) for shLockd contrasted shControl treated NIH/3T3 cells. The red dashed line denotes threshold for significance (p < 0.05). Black and grey colored genes represent significant and non-significant fold changes, respectively. **(E)** Schematic representation for the intersection of fold change (shLockd / shControl) against gene-gene correlations (in shControl treated NIH/3T3 cells)**. (F)** Relative expression of *Kif14* upon ASO induced knockdown of Lockd, measured by RT-qPCR (n = 5 biologically independent samples). **B, F**; data presented as mean values + /- s.e.m., p-values represent a two-sided Student’s t-test.

Transcription of the *Lockd* gene functions in *cis* by promoting expression of the cell cycle regulator *Cdkn1b* gene (10 kb upstream of the *Lockd* locus; Extended Data Fig. 6a) in a manner where the Lockd transcript itself was reported dispensable^30^ and without apparent function. In contrast, on shRNA-mediated Lockd transcript knockdown in NIH/3T3 cells, we observed reduced colony formation capacity (Fig. 3d), thus suggesting additional RNA-dependent functions. To complement the stable Lockd knockdown experiment, we designed two siRNAs and one antisense oligo (ASO) (Supplementary Table 5b) against the Lockd transcript with good knockdown efficiency (<25% remaining expression) in NIH/3T3 cells and primary fibroblasts (Extended Data Fig. 6b). In agreement with the NIH/3T3-shLockd stable cell line (Fig. 3d), a consistent decrease in colony-forming cells was observed on siRNA- and ASO-induced Lockd depletion (Fig. 4a). In line with a previous report^30^, no consistent change in RNA expression was observed for Cdkn1b on knockdown of the Lockd transcript in NIH/3T3 or primary fibroblast cells, although siLockd-3 induced the mRNA expression of Cdkn1b in primary fibroblasts (Fig. 4b). However, the allele-resolved scRNA-seq data suggested coexpression of Lockd and Cdkn1b (which tended to be expressed in the same cells and from the same allele) on both the CAST and C57 alleles (Extended Data Fig. 6c).

To characterize the molecular function of the Lockd transcripts in more detail, we generated scRNA-seq data from stable shLockd (*n* = 144) and shControl (*n* = 147) cells. Using SCDE^31^, we observed that 752 genes had significantly altered expression in the shLockd cells (292 genes upregulated and 460 genes downregulated) (Extended Data Fig. 6d and Supplementary Table 6). Next, we filtered for genes that had expression levels that correlated with Lockd expression in shControl cells. Requiring a positive correlation and reduced expression in the shLockd cells, or a negative correlation and increased expression in shLockd cells (Extended Data Fig. 6e), we refined the list of candidate genes to 138, which included several well-established cell cycle regulators (Supplementary Table 6). Particularly, three members of the kinesin superfamily (*Kif4*, *Kif11* and *Kif14*, all among the top 15 ranked genes based on positive Spearman correlations), a group of genes encoding proteins known to be involved in mitosis, appeared as main candidates (Fig. 4c). Notably, a link between these genes and the Cdkn1b protein has been suggested. While Cdkn1b acts as a transcriptional suppressor by binding to the *Kif11* promoter through a p130/E2F4-dependent mechanism^32^, Kif14 regulates the protein levels of Cdkn1b through a proteasome-dependent pathway^33^. Based on these previous findings, we set out to directly confirm the effect on *Kif4*, *Kif11* and *Kif14* by measuring expression levels with RT–qPCR on siRNA-induced knockdown of Lockd in NIH/3T3 and primary fibroblast cells. The effect on *Kif11* and *Kif14* was seen in both cell lines while the effect on *Kif4* could only be observed in primary fibroblasts (Fig. 4d,e). However, this is consistent with the scRNA-seq data of NIH/3T3 cells (Fig. 4c) where *Kif4* was more modestly affected compared to *Kif11* and *Kif14*. The effect on *Kif14* was also confirmed on ASO-induced depletion of Lockd (Extended Data Fig. 6f). In summary, while transcription of the *Lockd* gene has been reported to promote transcription of *Cdkn1b*^30^ in *cis*, we observed additional effects on Lockd transcript knockdown that appeared to function in the same pathway as Cdkn1b and enhanced the negative effects on cell cycle progression.

### Functional investigation of the lncRNA Wincr1

**Fig. 5:**
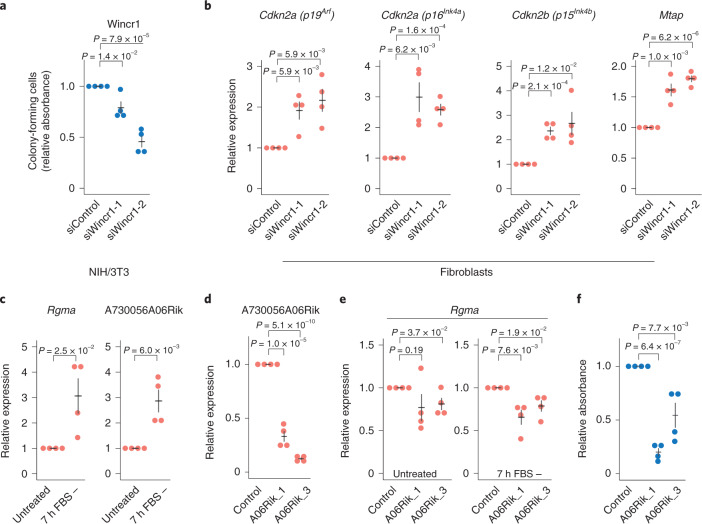
Functional analysis of lncRNAs Wincr1 and A730056A06Rik. **a**, Quantification of colony-forming cells on siRNA-induced knockdown of Wincr1 in NIH/3T3 cells. **b**, Relative expression of candidate *cis*-interacting genes on siRNA-induced knockdown of Wincr1 in primary fibroblast cells measured by RT–qPCR. **c**, Relative expression of *Rgma* and A730056A06Rik measured by RT–qPCR after 7-h serum starvation in NIH/3T3 cells. **d**, Relative expression of A730056A06Rik on ASO-mediated knockdown in NIH/3T3 cells measured by RT–qPCR. **e**, Relative expression of *Rgma* on serum starvation and ASO-induced knockdown of A730056A06Rik measured by RT–qPCR. **f**, Quantification of colony-forming cells on ASO-induced knockdown of A730056A06Rik in NIH/3T3 cells. Throughout the figure, *n* = 4 biologically independent samples; data are presented as mean values ± s.e.m.; *P* values represent a two-sided Student’s *t*-test.

**Extended Data Fig. 7:**
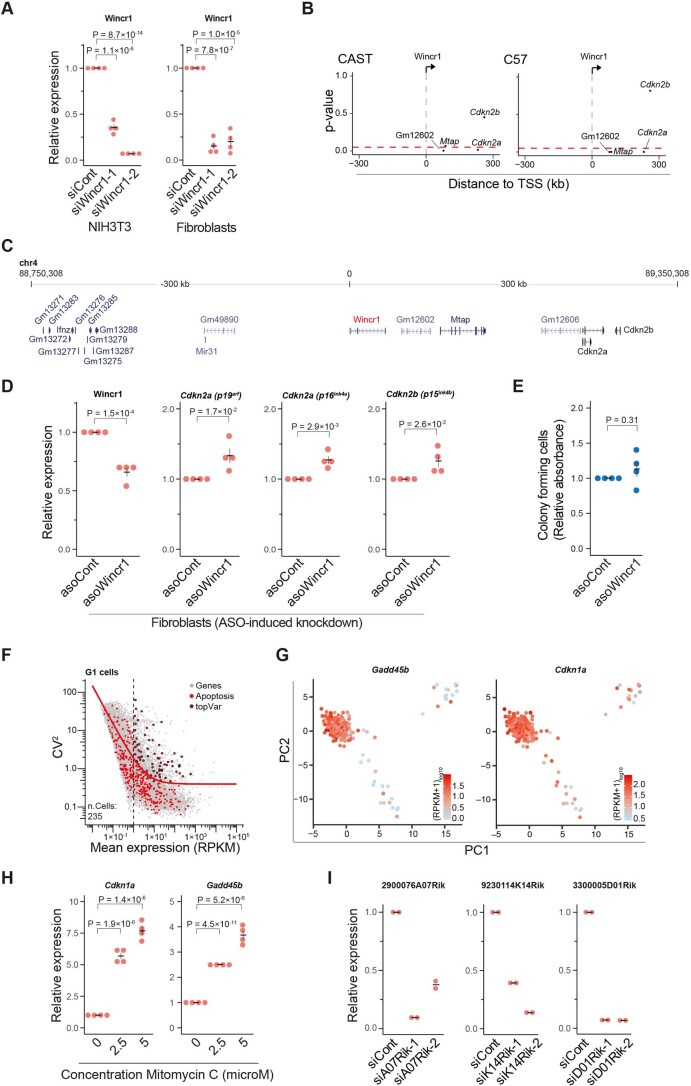
Analysis of Wincr1 and identification of apoptosis-associated lncRNAs. **(A)** Relative expression of Wincr1 upon siRNA induced knockdown in NIH/3T3 cells (left) and primary fibroblasts (right) measured by RT-qPCR (n = 4 biologically independent samples). **(B)** Scatter plots showing p-values for co-expression of Wincr1 (Fisher’s exact test) against other genes in proximity (± 300 kb of TSS-Wincr1, genes filtered for > = 3 allelic counts) for the CAST and C57 genomes. The red dashed lines denote threshold for significance (p < 0.05). **(C)** A schematic from the UCSC genome browser representing the *Wincr1* gene locus. **(D)** Relative expression of candidate *cis*-interacting genes upon ASO induced knockdown of Wincr1 in primary fibroblast cells measured by RT-qPCR (n = 4 biologically independent samples). **(E)** Quantification of colony forming cells upon ASO induced knockdown of Wincr1 in NIH/3T3 cells (n = 4 biologically independent samples). **(F)** Scatter plot showing the 75 most variable genes related to apoptosis, on top of a scatter plot showing mean expression levels (x-axis) and CV^2^ (y-axis) (n = 235 cells). **(G)** Low dimensional PCA projections of cells based on the most variable genes identified in (F), colored by the expression of *Gadd45b* and *Cdkn1a* (n = 235 cells). **(H)** Relative expression measured by RT-qPCR for *Gadd45b* and *Cdkn1a* in NIH/3T3 cells treated with MMC (n = 4 biologically independent samples). **(I)** Relative expression of candidate lncRNAs upon siRNA induced knockdown in NIH/3T3 cells measured by RT-qPCR (n = 2 biologically independent samples, horizontal lines represent the mean expression). **A, D, E, H**; data presented as mean values + /- s.e.m., p-values represent a two-sided Student’s t-test.

To explore the molecular function of Wincr1 (ref. ^34^) in greater detail, we designed two siRNAs against Wincr1 and confirmed their knockdown by RT–qPCR (Extended Data Fig. 7a). As observed in the shWincr1 stable NIH/3T3 cell line, loss of Wincr1 decreased colony-forming cells at magnitudes that corresponded to siRNA depletion efficiency (Fig. 5a and Extended Data Fig. 7a). Analyzing the Smart-seq2 scRNA-seq data (Extended Data Fig. 1a–c) identified several closely located genes with expressions that were coordinated with Wincr1, including *Cdkn2a* (encoding *p16*^*Ink4a*^ and *p19*^*Arf*^), Gm12602 and *Mtap* (Extended Data Fig. 7b,c). Intriguingly, the homologous loci in humans have been reported to regulate the expression of *CDKN2A* (*p16*^*INK4A*^) in a mechanism where the microRNA-31 host gene (MIR31HG) recruits chromatin remodeling factors to the promoter of *p16*^*INK4A*^ (ref. ^35^). However, *Mir31hg* has a different genomic structure in the mouse and Wincr1 is absent in human cells. siRNA-mediated Wincr1 knockdown in primary fibroblasts (approximately 75% depletion; Extended Data Fig. 7a) resulted in the significant increase in *Cdkn2a* (*p16*^*Ink4a*^ and *p19*^*Arf*^), *Cdkn2b* (*p15*^*Ink4b*^) and *Mtap* expression (Fig. 5b), an effect that was further confirmed by ASO-induced knockdown (Extended Data Fig. 7d). However, the effect on *Cdkn2a* (*p16*^*Ink4a*^ and *p19*^*Arf*^) and *Cdkn2b* (*p15*^*Ink4b*^) was lower on ASO-induced knockdown, in line with their less efficient Wincr1 knockdown (approximately 40% depletion; Extended Data Fig. 7d), and did not affect the colony-forming capacity of the cells, likely due to the incomplete knockdown (Extended Data Fig. 7e). We note that *Cdkn2a* (*p16*^*Ink4a*^ and *p19*^*Arf*^)^36^ and *Cdkn2b* (*p15*^*Ink4b*^)^36^ are inactivated in NIH/3T3 cells due to homozygous deletions of their chromosomal regions; therefore, they are not involved in the colony-forming capacity of NIH/3T3 cells on Wincr1 knockdown (Fig. 5a).

### Functional investigation of the lncRNA A730056A06Rik

We noted that A730056A06Rik is a natural antisense transcript to *Rgma* (involved in cell survival^37^) and found both to be induced on serum starvation (Fig. 5c). To investigate their molecular interaction, we designed two ASOs against A730056A06Rik (Fig. 5d). We measured the ASO effect in both untreated cells and on serum starvation and observed that *Rgma* expression was lowered by the ASOs in both serum-starved and untreated cells (with three of the four conditions reaching statistical significance, Fig. 5e). Unexpectedly, we observed a decrease in colony-forming cells on ASO-mediated A730056A06Rik knockdown (Fig. 5f), in contrast to the effect seen in the stable lentiviral transduced cells (Fig. 3d). In summary, the ASO-mediated knockdowns support the function of A730056A06Rik on *Rgma*, while the effects on colony formation are inconclusive and need further evaluation. We speculate that these disparities could relate to shRNA off-target effects^38^, their different modes of knockdown (to target spliced or unspliced transcripts) or potentially compensatory effects in long-term (shRNAs) versus short-term (ASOs) knockdowns.

### Generalization of lncRNA functions to other phenotypes

**Fig. 6:**
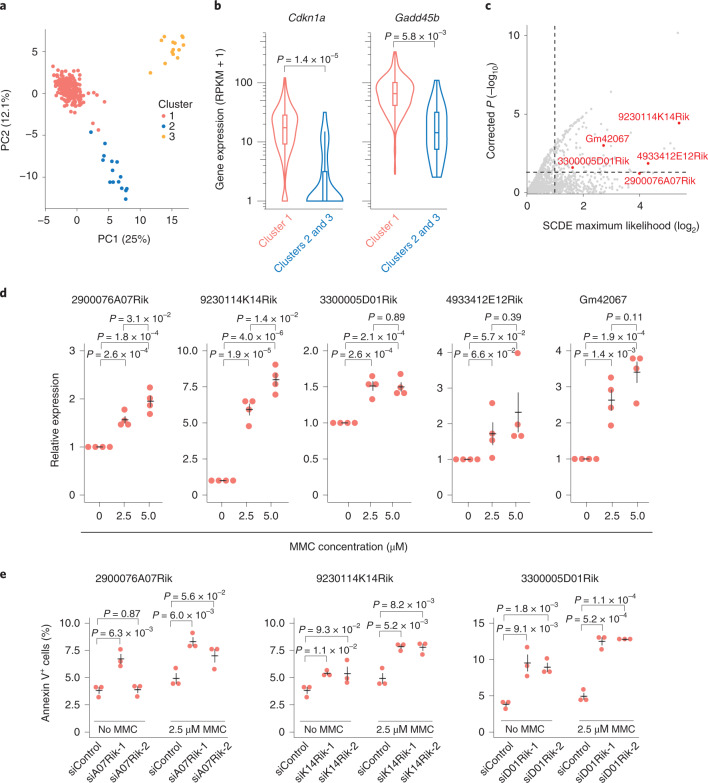
Identification of lncRNAs involved in apoptosis by single-cell profiling. **a**, Low-dimensional PCA projection of cells using the most variable apoptosis-related genes. Cells are colored according to clusters. **b**, Violin plots showing the expression levels of two marker genes (box plots: the center lines show the medians, the interquartile limits indicate the 25th and 75th percentiles, the whiskers denote the farthest points at a maximum of 1.5 times the IQR; *P* values represent a two-sided Wilcoxon test). **c**, Scatterplot showing the fold change magnitudes (*x* axis, SCDE maximum likelihood estimate of the fold change) and significance levels (*y* axis, SCDE *P* value, two-sided test using the multiple testing-corrected (Holm procedure) z-score) for cluster 1 against clusters 2 and 3 identified in **a**. The lncRNAs selected for validation are marked in red. **d**, Relative expression measured by RT–qPCR of candidate lncRNAs in NIH/3T3 cells treated with MMC (*n* = 4 biologically independent samples). **e**, Quantification of apoptosis using annexin V for siRNA-targeted NIH/3T3 cells treated with MMC (*n* = 3 biologically independent samples). **d**,**e**, Data are presented as mean values ± s.e.m.; *P* values represent a two-sided Student’s *t*-test.

We next generalized the strategy to an additional cellular state, by investigating lncRNAs involved in apoptotic signaling. Since apoptotic signaling is linked to proliferation, we based the analysis to cells in the G1 phase (Fig. 3a) and repeated the low-dimensional projection, now using the most variable genes related to apoptotic signaling (using GO:0043065; Extended Data Fig. 7f). We focused specifically on one cluster of cells that expressed genes involved in growth arrest and DNA damage, exemplified by *Gadd45b*^39^ and the p53 target gene *Cdkn1a* (Fig. 6a,b and Extended Data Fig. 7g). Again, SCDE^31^ was applied to find lncRNAs with increased expression in this cluster of cells and we could design siRNAs against five highly ranked lncRNAs (based on adjusted *P* values and fold changes) (Fig. 6c). To investigate these candidates, DNA damage-induced apoptosis was triggered in NIH/3T3 cells by the chemotherapeutic and DNA cross-linking reagent mitomycin C (MMC). DNA damage was validated by increased *Cdkn1a* and *Gadd45b* expression using RT–qPCR (Extended Data Fig. 7h); importantly, expression of the five candidate lncRNAs was induced on MMC treatment, with two lncRNAs having expressions in an MMC concentration-dependent manner (Fig. 6d). To further investigate the regulatory effects of these lncRNA on apoptosis, three of the candidates were suppressed by two siRNAs each (Extended Data Fig. 7i). The levels of apoptosis in lncRNA-suppressed NIH/3T3 cells was measured by annexin V on flow cytometry after treatment with MMC (Fig. 6e). Notably, apoptosis was repeatedly induced when exposed to MMC, suggesting that knockdown of these lncRNAs sensitizes cells to undergo apoptosis. In summary, the separation of cellular transcriptomes according to state-dependent cellular processes, exemplified in this study by more subtle proapoptotic signaling, was efficient in predicting lncRNA phenotypes.

### Allele-resolved expression identifies *cis*-functioning lncRNAs

**Fig. 7:**
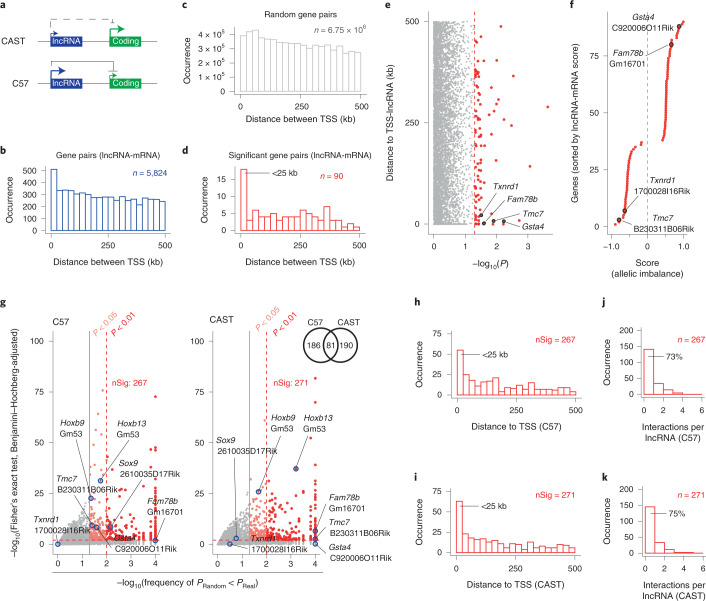
Identification of *cis*-functioning lncRNAs using allele-resolved expression. **a**, Schematic illustration of hypothetical *cis* effects between lncRNAs and proximal mRNAs that could manifest as coordinated allelic expression dynamics across cells. **b**, Histogram showing lncRNA-mRNA gene pairs within ± 500 kb of the lncRNA TSS. **c**, Histogram showing permutated lncRNA-mRNA gene pairs (where each lncRNA was moved to 1,000 randomly selected gene locations). **d**, Histogram showing significant lncRNA-mRNA gene pairs (*P* < 0.05, permutation test). **e**, Scatterplot representing *P* values (permutation test) against the distance between lncRNA and mRNA pairs of genes. Significant gene pairs are colored in red; the dashed red line represents *P* = 0.05. **f**, Scatterplot representing the ranking of significant lncRNA-mRNA pairs of genes. A positive value (*x* axis) represents allelic imbalance toward the same allele while a negative value represents imbalance on opposite alleles. The highlighted lncRNA-mRNA pairs of genes were considered for downstream validation. **g**, Scatterplot where the *x* axis represents *P* values (permutation test where each lncRNA was moved to 1,000 randomly selected gene locations) against the *y* axis, which represents *P* values of a Fisher’s exact test (Benjamini–Hochberg-adjusted) of lncRNA-mRNA gene pairs within 500 kbp of each lncRNA TSS. Significant gene pairs are colored in red. The dashed red line represents *P* = 0.01, the solid light red line represents *P* = 0.05. The Venn diagram represents significant lncRNA-mRNA pairs of genes for the C57 and CAST genomes. **h**,**i**, Histogram showing significant lncRNA-mRNA pairs of genes for the C57 (**h**) and CAST (**i**) genomes. **j**,**k**, Histogram representing the number of significant interactions for individual lncRNAs for the C57 (**j**) and CAST (**k**) genomes.

**Extended Data Fig. 8:**
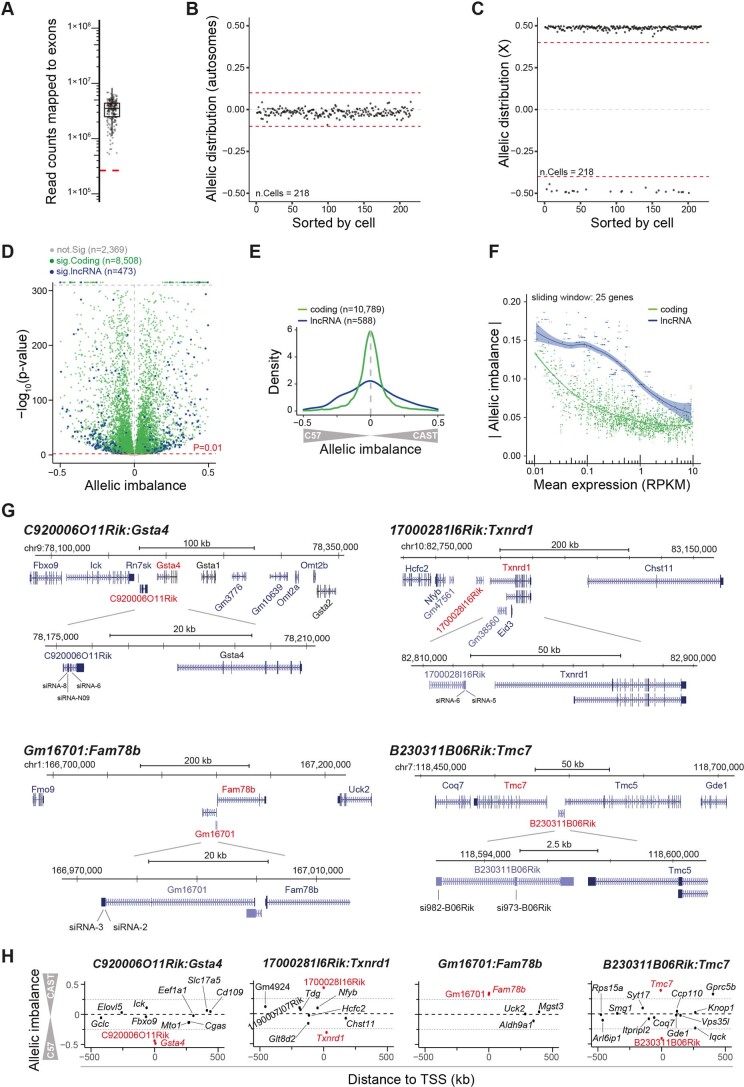
Control experiments on allelic imbalance in fibroblast cells. **(A-C)** Quality control for Smart-seq2 libraries from CASTxC57 primary fibroblast cells. Red lines represent quality control cutoff. **(A)** Boxplot showing number of sequenced reads counts mapped to exons (Smart-seq2 libraries, n = 218 cells). The center line shows the median, interquartile limits indicate the 25^th^ and 75^th^ percentiles and whiskers denote the farthest points at a maximum of 1.5 times the IQR. **(B)** Scatter plot showing the distribution of allele sensitive read counts for non-imprinted autosomal genes. **(C)** Scatter plot showing the distribution of allele sensitive read counts for non-escapee genes on the X-chromosome. **(D)** Scatter plot of the allelic imbalance against p-values (binomial test, Benjamini-Hochberg adjusted) across fibroblasts (n = 751 cells). **(E)** Density plot summarizing observed allelic imbalance of lncRNAs and mRNAs across fibroblasts (n = 751 cells). **(F)** Mean expression towards the median of allelic imbalance of a sliding window (width = 25) for mRNAs and lncRNAs. The green and blue lines denote a loess fit to the sliding window with a 95% confidence interval for mRNAs and lncRNAs, respectively. **(G)** Schematics from the UCSC genome browser representing the gene loci of four candidate lncRNA-mRNA gene pair interactions. **(H)** Scatter plot representing allelic imbalance of genes within + /−500kb (of lncRNA TSS) of candidate genes. Candidate lncRNA-mRNA gene pair interactions are colored in red.

Allelic imbalance in gene expression across heterozygous F1 hybrid mice is pervasive^40^ and we next investigated if the allelic imbalance of lncRNAs could reveal information about *cis*-regulatory mechanisms and gene–gene interactions (Fig. 7a). To improve the power to detect gene–gene interactions, we profiled an additional 218 mouse adult tail fibroblasts (by Smart-seq2) resulting in 751 postquality control cells (Extended Data Fig. 8a–c and Extended Data Fig. 1a–c). We counted allele-informative reads across all cells to quantify allelic imbalance as (CAST_allelicCounts_ /(CAST_allelicCounts_ + C57_allelicCounts_) − 0.5) where a positive score reflects increased RNA expression toward the CAST genome. Consistent with previous bulk RNA-seq studies^40^, we confirmed that approximately 75% of mouse genes (8,981 of 11,350) had RNA expression levels dependent on the genetic background (Extended Data Fig. 8d). lncRNAs had stronger allelic imbalance than mRNAs (Extended Data Fig. 8e) across a wide range of expression levels (Extended Data Fig. 8f). To identify *cis*-functioning lncRNAs, we first retrieved all lncRNA-mRNA gene pairs (with allelic coverage) within ± 500 kb of each lncRNA transcription start site (TSS) (5,824 pairs in total; Fig. 7b) and calculated a score for allelic imbalance for each lncRNA-mRNA gene pair (Methods). Next, a permutation test was applied, where each lncRNA was moved to 1,000 randomly selected gene locations and the score for in silico sampled gene pairs recomputed (±500 kb of the lncRNA TSS, 6.8 M random gene pairs in total; Fig. 7c). In total, 90 significant lncRNA-mRNA interactions were identified (Supplementary Table 7) and the significant gene pairs were enriched at closer distance (within 25 kb; Fig. 7d,e). We sorted the significant interactions (Methods) according to coordinated allelic imbalances (Fig. 7f) and selected four highly ranked lncRNA-mRNA interactions that were accessible to siRNA depletion, within 25 kb of each other and with diverse genomic organization (Extended Data Fig. 8g,h).

**Extended Data Fig. 9:**
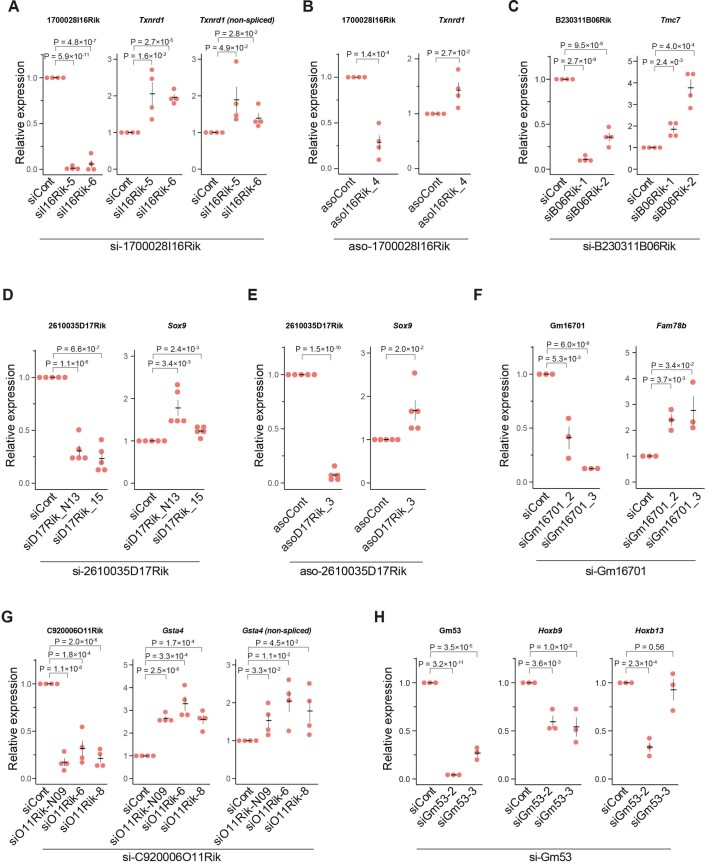
Validation experiments on lncRNA-mRNA interactions. **(A-H)** Relative expression levels upon siRNA/ASO induced knockdown in primary fibroblasts of **(A)** *Txnrd1* upon siRNA induced knockdown of 1700028I16Rik (n = 4 biologically independent samples), **(B)** *Txnrd1* upon ASO induced knockdown of 1700028I16Rik (n = 4 biologically independent samples), **(C)** *Tmc7* upon siRNA induced knockdown of B230311B06Rik, **(D)** *Sox9* upon siRNA induced knockdown of 2610035D17Rik (n = 5 biologically independent samples), **(E)** *Sox9* upon ASO induced knockdown of 2610035D17Rik (n = 5 biologically independent samples), **(F)** *Fam78b* upon siRNA induced knockdown of Gm16701, **(G)** *Gsta4* upon siRNA induced knockdown of C920006O11Rik and **(H)** *Hoxb9* and *Hoxb13* upon siRNA induced knockdown of Gm53. **A-H**; measured by RT-qPCR, data presented as mean values + /- s.e.m., p-values represent a two-sided Student’s t-test.

In parallel, we assessed if allele-specific expression patterns at the single-cell level could be used as a strategy to identify pairs of potential *cis*-regulatory function for in-depth molecular characterization. Evaluating the same set of lncRNA-mRNAs gene pairs as above (5,824 gene pairs ± 500 kb of the lncRNA TSS; Fig. 7b), a Fisher’s exact test was applied to each gene pair (*P*_Real_, Benjamini–Hochberg-adjusted) and also for in silico sampled gene pairs by moving each lncRNA to 1,000 randomly selected gene locations (*P*_Random_, Benjamini–Hochberg-adjusted, as in Fig. 7c–e; Methods). These criteria identified significant coordinated expression of 457 lncRNA-mRNA gene pairs on at least 1 allele (Fig. 7g and Supplementary Table 8). The gene pairs were enriched at a closer distance (<25 kb; Fig. 7h,i) and most lncRNAs had only 1 significant interaction (Fig. 7j,k). Encouraged to see that several of the candidates overlapped between the population and single-cell resolution approaches (Fig. 7e,g), we next functionally dissected a subset of interactions. We selected six lncRNA-mRNA gene pairs, covering two that were identified by both approaches (B230311B06Rik*:Tmc7* and Gm16701*:Fam78b*), two by allelic imbalance (1700028I16Rik*:Txnrd1* and C920006O11Rik*:Gsta4*) and two by the single-cell strategy (2610035D17Rik*:Sox9* and Gm53*:Hoxb13*). We also noted that the lncRNA Gm53 showed a second significant interaction with *Hoxb9* (in addition to *Hoxb13*) at a slightly lower significance threshold (0.01 < *P* < 0.05) (Fig. 7g). To evaluate these molecular interactions, we designed at least two siRNAs against each lncRNA and measured the effects with RT–qPCR. All candidate gene pairs were confirmed to show the expected target mRNA expression change (Extended Data Fig. 9a–h) and we also validated an increase in unspliced RNA levels for *Txnrd1* and *Gsta4* (Extended Data Fig. 9a,g), which indicated an effect on transcription. In addition, ASOs toward 2610035D17Rik and 1700028I16Rik had similar effects (Extended Data Fig. 9b,e) as the siRNAs.

**Fig. 8:**
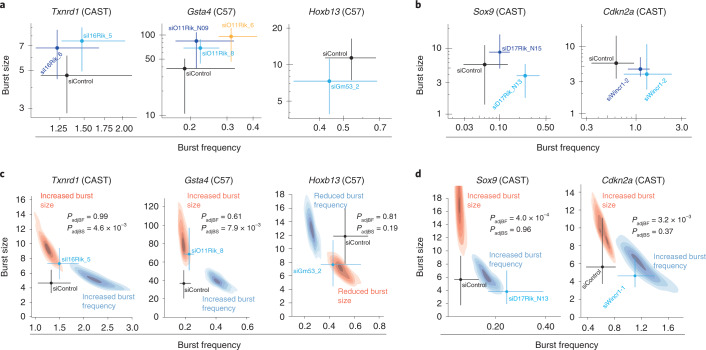
The effect of lncRNAs on transcriptional bursting. **a**, Scatterplots representing burst parameters with 95% CIs for *Txnrd1*, *Gsta4* and *Hoxb13* on siRNA-induced knockdown of lncRNAs. **b**, Scatterplots representing burst parameters with 95% CIs for *Sox9* and *Cdkn2a* on siRNA-induced knockdown of lncRNAs. **c**, Scatterplots representing burst parameters with 95% CIs for *Txnrd1*, *Gsta4* and *Hoxb13* on siRNA-induced knockdown of lncRNAs (for 1 representative siRNA from **a**). Distribution of simulated cases (100 simulations) when expression was modulated by burst frequency or size is shown in blue and red, respectively. **d**, Scatterplots representing burst parameters with 95% CIs for *Sox9* and *Cdkn2a* on siRNA-induced knockdown of lncRNAs (for 1 representative siRNA from **b**). Distribution of simulated cases (100 simulations) when expression was modulated by burst frequency or size is shown in blue and red, respectively. **a**–**d**, Colored by siRNA. **c**,**d**, *P* values represent a two-sided maximum likelihood ratio test.

**Extended Data Fig. 10:**
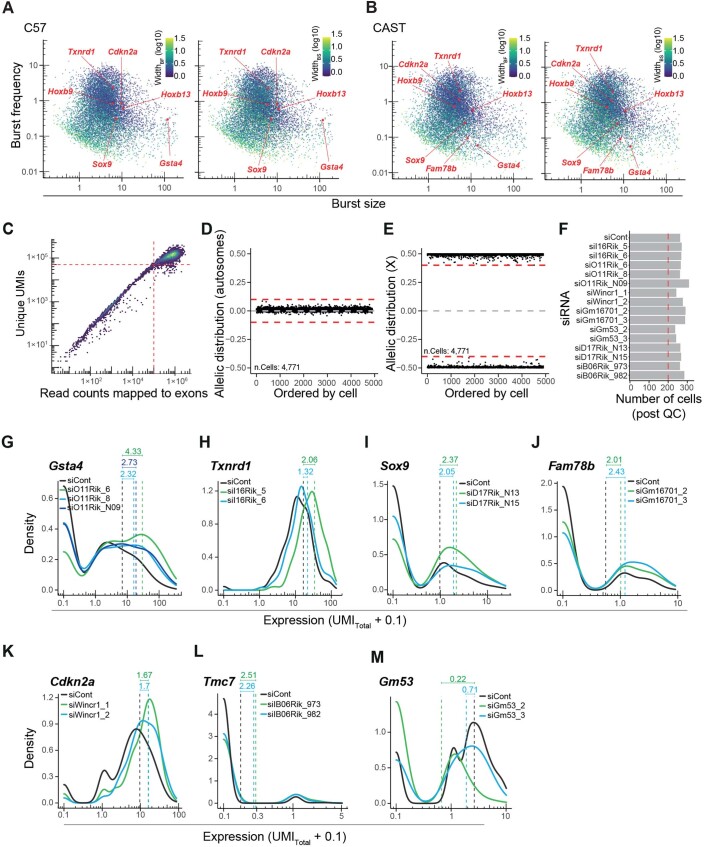
Analyses of lncRNAs’ effect on burst kinetics of nearby mRNAs. **(A,B)** Scatter plots showing the burst sizes against the burst frequencies for the C57 **(A)** and CAST **(B)** genomes. Genes are colored according to the width of a 95% confidence interval (CI_high_/CI_low_). **(C-E)** Quality control for Smart-seq3 libraries from siRNA treated primary fibroblast cells (red lines represent quality control cutoffs); scatter plot showing the number of unique UMI reads against the number of read counts mapped to exons **(C)**, scatter plot showing the distribution of allele sensitive read counts for non-imprinted autosomal genes **(D)**, and scatter plot showing the distribution of allele sensitive read counts for non-escapee genes on the X-chromosome **(E)**. **(F)** Barplot representing the number of cells passing quality control for individual siRNA treatments. **(G-L)** Density plots representing mean expression (UMIs) of modulated genes upon siRNA induced suppression of lncRNAs for *Gsta4* **(G)**, *Txnrd1* **(H)**, *Sox9* **(I)**, *Fam78b* **(J)**, *Cdkn2a* **(K)** and *Tmc7* **(L)**. **(M)** Density plot representing expression of the lncRNA Gm53 upon siRNA induced knockdown. **G-M**; mean expression fold changes are quantified and highlighted in green and blue. Vertical dashed lines represent mean expression levels.

While many lncRNAs affect transcription of nearby mRNAs, it is not known how lncRNAs alter their burst frequencies or sizes. To address this question, we further investigated the validated lncRNA-mRNA interactions (1700028I16Rik*:Txnrd1*, C920006O11Rik*:Gsta4*, Gm16701*:Fam78b*, B230311B06Rik*:Tmc7*, 2610035D17Rik*:Sox9*, Gm53*:Hoxb9*, Gm53*:Hoxb13* (Extended Data Fig. 9a–h) and Wincr1*:Cdkn2a* (Fig. 5b)) that had mRNA targets expressed in a part of the transcriptional kinetics parameter space for which we had good precision (narrow confidence intervals (CIs); Methods) for burst inference (Extended Data Fig. 10a,b). To obtain burst parameters across lncRNAs perturbations, we profiled individual adult tail fibroblasts with Smart-seq3 (ref. ^6^) on siRNA-induced knockdown and generated a comprehensive dataset with at least 200 cells (postquality control) for each siRNA knockdown (Extended Data Fig. 10c–f). We first compared the fold changes of the Smart-seq3 measurements (Extended Data Fig. 10g–l) with those of RT–qPCR (Extended Data Fig. 9a–h) and found generally good agreement with approximately similar fold changes (Supplementary Table 9). Noteworthy, knockdown of lncRNA-Gm53 using siGm53_3 was less efficient than siGm53_2 on both RT–qPCR (Extended Data Fig. 9h**)** and scRNA-seq measurements (Extended Data Fig. 10m); induction on *Txnrd1* was less robust for siI16Rik_6 compared to siI16Rik_5 (Extended Data Figs. 9a and 10h). We next inferred burst parameters for *Txnrd1*, *Gsta4*, *Sox9*, *Cdkn2a* and *Hoxb13* from the allele with the highest precision in burst inference (generally the highest expressed allele) since their allelic imbalance precluded bursting inference from both alleles, while *Tmc7* and *Fam78b* did not reach sufficient UMI counts and SNP coverage for burst inference from either allele. The inference showed a consistent effect on burst size for *Txnrd1, Gsta4* and *Hoxb13* (Fig. 8a), whereas *Sox9* and *Cdkn2a* showed an increase in burst frequency (Fig. 8b). Using simulations for one representative siRNA for each lncRNA, we demonstrated that the observed effects were in the regions of parameter space expected for an exclusive effect on either burst size (Fig. 8c) or burst frequency (Fig. 8d). Taken together, these observations suggest that lncRNAs can regulate both burst frequencies and burst sizes; it will be interestingly to further investigate the biochemical processes (that is, transcriptional initiation and elongation) that may be altered by lncRNAs.

## Discussion

Several studies have demonstrated lower lncRNA expression levels than mRNAs but the underlying molecular causes have remained unclear^2^. Using allele-resolved scRNA-seq, we discovered that low expression of lncRNAs is mostly governed by lowered transcriptional burst frequencies (Fig. 2d and Extended Data Fig. 4a) and the durations between two transcriptional bursts of lncRNAs on the same allele were approximately twice as long compared to mRNAs (Fig. 2g and Extended Data Fig. 4f). Notably, over 30% of lncRNAs were estimated to burst less than once every 24 h from each allele, suggesting that many lncRNA alleles may be inactive throughout an entire cell cycle. While the lowered burst frequency of lncRNAs (fourfold decrease; Fig. 2d and Extended Data Fig. 4a) likely represents a decrease in enhancer-mediated transcriptional initiation^7,41–43^, we also detected a more modest effect on burst size (twofold decrease; Fig. 2e and Extended Data Fig. 4b) that might reflect fewer transcription factor binding sites near core promoters of lncRNAs^8^.

Interestingly, pairs of genes that are divergently transcribed (lncRNA-mRNA as well as mRNA-mRNA gene pairs) had higher burst frequencies than genes separated by larger distances (Fig. 2l and Extended Data Fig. 4m). Divergent promoters typically harbor more transcription factor binding sites^8^ and their increased burst frequencies might result from positive interactions and more efficient recruitment of the required transcriptional complexes at two closely located promoters.

We also revisited the question whether lncRNAs have increased cell-to-cell variability in expression compared to mRNAs of similar expression. Although lncRNA expression patterns are heterogeneous (Fig. 1d), we observed in both mouse and human cells that lncRNAs had generally higher cell-to-cell variability compared to expression-matched mRNAs (Fig. 1e–g and Extended Data Fig. 2a,b). The increased number of lncRNAs measured in our study likely explains why earlier reports with fewer genes studied did not identify any increased variability of lncRNAs^15^ since subsampling lncRNAs to smaller numbers often reduced the statistical power needed to identify this increase (Extended Data Fig. 2c). Notably, we also found that the lncRNAs with the highest cell-to-cell variability were transcribed less frequently although with higher burst sizes (Fig. 2h,i and Extended Data Fig. 4i,j).

Analysis of scRNA-seq data also allowed us to identify putative functions of lncRNAs. Specific lncRNA expression in transient cellular states, for example, cell cycle and proapoptotic states, was predictive of lncRNA functions in particular cellular condition without the need for initial perturbation experiments. Notably, knockdown of several identified lncRNAs had only apparent phenotypes when exposed to relevant stress (Fig. 3d) and are therefore likely missed in large genome-wide perturbation studies carried out at steady-state growth conditions. Finally, identifying mRNA genes correlated to lncRNAs across untreated cells, in combination with differential expression on lncRNA knockdown, was highly useful for decoding lncRNA functions (Extended Data Fig. 6e) by revealing the most relevant targets for Lockd (Fig. 4c). In summary, our functional analysis covers several siRNAs, ASOs and stable lentiviral transduced cell lines, well-established strategies to study loss of function in lncRNAs. However, each approach has different off-target spectra and may induce unintended effects^38^. For example, the effects of ASO-induced premature termination of transcription^44^, siRNA-induced off-target effects, differences in acute (siRNAs-induced knockdown) versus long-term (stable cell lines with shRNA-induced knockdown) and siRNA-induced transcriptional gene silencing/activation^45^, should never be overlooked.

Finally, we explored how lncRNAs may modulate burst kinetics of nearby protein-coding genes. Although the regulation of transcriptional bursting is generally poorly understood, we showed that lncRNAs can modulate both burst sizes and burst frequencies (Fig. 8a–d). Clearly, more lncRNA-mRNA interactions need to be characterized in greater detail to investigate if certain lncRNA-mRNA orientations (for example, antisense, divergent promoters) may be associated with similar transcriptional bursting effects. Yet, our observations suggest that lncRNAs are involved in the biochemical processes that control the initiation frequencies of transcription (by modulating burst frequency; Fig. 8b,d) or the numbers of RNA polymerase II complexes that get loaded during an active burst (by modulating burst size; Fig. 8a,c). The precision of the inferred burst parameters are gene-specific (Extended Data Fig. 10a,b) and dependent on the expression levels, SNP coverage, the number of cells sequenced and the sequencing depth of the experiments (two out of seven scRNA-seq experiments failed due to the genes studied having too large CIs). The development of more sensitive scRNA-seq protocols, lowered cost for sequencing and a general increased throughput of cells should improve the precision in burst inference and allow for analysis at larger scales.

## Methods

### Ethical compliance

The research carried out in this study has been approved by the Swedish Board of Agriculture, Jordbruksverket: N343/12.

### Cell culture

Mouse primary fibroblasts were derived from adult (>10 weeks old) CAST/EiJ × C57BL/6J or C57BL/6J × CAST/EiJ mice by skinning, mincing and culturing tail explants (for at least 10 d) in DMEM high glucose, 10% embryonic stem cell FBS, 1% penicillin/streptomycin, 1% nonessential amino acids (NEAAs), 1% sodium pyruvate, 0.1 mM 2-mercaptoethanol (Sigma-Aldrich) in culture dishes coated with 0.2% gelatin (Sigma-Aldrich). NIH/3T3 cells were maintained in DMEM supplemented with 10% FBS and 1% penicillin/streptomycin. All supplements were purchased from Thermo Fisher Scientific (unless stated otherwise).

### Generation of Smart-seq2 libraries

Smart-seq2 libraries were prepared as described earlier^18^ using the following parameters: (1) 20 cycles of PCR for preamplification; (2) a ratio of 0.8:1 for bead:sample purification of preamplified complementary DNA (in-house-produced 22% polyethylene glycol (PEG) beads); (3) tagmentation of approximately 1 ng bead-purified cDNA (in-house-generated Tn5 (ref. ^46^)); (4) 10 cycles of PCR for library amplification of the tagmented samples using Nextera XT Index primers; and (5) a ratio of 1:1 for bead purification of DNA sequencing libraries (in-house-produced 22% PEG beads). Sequencing was carried out on an Illumina HiSeq 2000 generating 43 base pair (bp) single-end reads. The libraries related to Figs. 1, 3 and 6 were derived from one tail explant (F1 offspring of C57 × CAST mouse female adult) and combined with previously published Smart-seq2 data^20^. The additional Smart-seq2 data generated for Fig. 7 were derived from one additional tail explant (F1 offspring of CAST × C57 mouse female adult).

### Generation of Smart-seq3 libraries

Smart-seq3 libraries were generated according to a previously published protocol^6^. Cells were stained with propidium iodide (PI) before being sorted (BD FACSMelody 100 μM nozzle; BD Biosciences) into 384 well plates containing 3 μl of Smart-seq3 lysis buffer (5% PEG (Sigma-Alrich), 0.10% Triton X-100 (Sigma-Aldrich), 0.5 U μl^−1^ of recombinant RNase inhibitor (Takara), 0.5 μM Smart-seq3 oligo(dT) primer (5′-biotin-ACGAGCATCAGCAGCATACGA T_30_VN-3′; Integrated DNA Technologies), 0.5 mM deoxynucleoside triphosphate (Thermo Fisher Scientific)) and stored at −80 °C. From this point, the standard protocol for Smart-seq3 was applied: (1) 20 cycles of PCR for preamplification of cDNA; (2) a ratio of 0.6:1 for bead:sample purification of preamplified cDNA (in-house-produced 22% PEG beads); (3) tagmentation of 150 ng bead-purified cDNA using 0.1 μl of Amplicon Tagment Mix; and (4) 12 cycles of PCR for library amplification of the tagmented samples using custom-designed Nextera Index primers containing 10-bp indexes. Samples were pooled, bead-purified at a ratio of 0.7:1 (in-house-produced 22% PEG beads) and prepared for sequencing on a DNBSEQ-G400RS (MGI) generating 100-bp paired-end reads. The data related to Fig. 2 were obtained from one tail explant (F1 offspring of C57 × CAST female adult mouse) and is also part of a previous study^47^. The libraries with siRNA-perturbed lncRNAs (related to Fig. 8) were derived from one tail explant (F1 offspring of C57 × CAST female adult mouse).

### Processing of RNA-seq data

A subset of primary fibroblasts analyzed in this study (sequenced by Smart-seq2) are part of previously published studies and were reanalyzed for consistency^7,20^ (NCBI Sequence Read Archive ID SRP066963). The zUMIs v.2.7.1b pipeline^48^ was used for alignment (mm10 assembly), gene quantification (Ensembl, GRCm38.91) and allelic calling for primary fibroblast data. To pass quality control, cells were required to have (**1)** ≥500,000 reads, (2) 4,000 genes expressed at ≥ 5 read counts, (3) distribution of allelic counts within −0.10 < allelic SNPs < 0.10 on autosomes (imprinted and genes on the X chromosome excluded) and (4) no more than 20% of allelic counts mapped to the imprinted X chromosome (escapee genes excluded). Genes with at least five read counts in two cells were kept for downstream analysis (unless stated otherwise).

Smart-seq3 libraries of HEK293 cells had previously been generated by Hagemann-Jensen et al.^6^ (ArrayExpress ID E-MTAB-8735). The zUMIs v.2.7.0a pipeline^48^ was used for alignment (hg38 assembly) and quantification of gene expression (Ensembl, GRCh38.95). Cells were required to have (1) ≥500,000 read counts mapped to exons and (2) ≥7,500 genes (≥1 read count). Genes with at least one read count in three cells were considered for downstream analysis. Gene types were annotated according to BioMart release 91.

The Smart-seq2 libraries of mouse embryonic stem cells had previously been generated by ﻿Ziegenhain et al.^19^ (Gene Expression Omnibus (GEO) ID GSE75790). The zUMIs v.2.7.2a pipeline was used for alignment (mm10 assembly) and quantification of gene expression (Ensembl, GRCm38.91). Cells were required to have ≥ 400,000 read counts mapped to exons and ≥8,000 genes (≥5 read counts). Genes with at least five read counts in two cells were considered for downstream analysis and gene types were annotated according to Supplementary Table 2 (downloaded from https://m.ensembl.org/biomart/martview/; gene list also available at https://github.com/sandberg-lab/lncRNAs_bursting).

For the Smart-seq3 libraries of primary fibroblasts treated with siRNAs, the zUMIs v.2.9.4b pipeline^48^ was used for alignment (mm10 assembly) and quantification of gene expression (Ensembl, GRCh38.95). Cells were required to have (1) ≥100,000 read counts mapped to exons, (2) ≥50,000 unique UMI counts and (3) ≥5,000 genes (≥1 UMI count). Genes with at least one UMI count in three cells were considered for downstream analysis.

### Annotation of lncRNAs

The Ensembl BioMart annotation (GRCm38.p6; Supplementary Table 2) was used to assign lncRNAs. Genes were first filtered (above) and lncRNAs categorized as: (1) divergent (no gene–gene overlap and TSS not separated by more than 500 bp); (2) convergent (gene–gene overlap and TSS not separated by more than 2 kb); (3) intergenic (no gene–gene overlap and at least 4 kb from any other expressed gene); and (4) separated transcriptional units (TSS separated with at least 4,000 bp from any other expressed gene). The threshold of 4 kb was established by manual inspection of Extended Data Fig. 1d where mean expression had been measured (median of sliding window size = 51) against the distance between the 2 most closely located TSSs (only genes passing quality control were considered for these analysis).

### Permutation test for CV^2^

For the analysis of cell-to-cell variability, only genes meeting the following criteria were considered: (1) not imprinted; (2) not encoded on the X chromosome; and (3) being classified as separated transcriptional units (Extended Data Fig. 1d).

#### CV^2^

For each lncRNA meeting the criteria, ten separated transcribed protein-coding genes having the most similar mean expression (min(mean(RPKM_lncRNA_) − mean(RPKM_mRNA_))) were selected. The matching allowed for the same protein-coding gene to be selected multiple times (sample replacement). For the permutation test (*n* = 10,000), 1 expression-matched protein-coding gene was randomly sampled for each lncRNA and the expected CV^2^ (median) was calculated for each permutation. The *P* value represents the frequency of median(CV^2^_sampled_) > median(CV^2^_lncRNA_).

To estimate the number of lncRNAs needed to detect median(CV^2^_lncRNA_) > median(CV^2^_mRNA_) (Extended Data Fig. 2c), the permutation test was repeat 100 times for each subsampling size (between 10 and 200 lncRNAs) of the frequency where 50% and 95% of the permutations reached median(CV^2^_lncRNA_) > median(CV^2^_sampled_) was assessed.

### Transcriptional bursting kinetics inference

Transcriptional bursting kinetics were inferred from homogenous sets of cells using the two-state model of transcription, based on previous methodology^7^. In detail, we first computed the UMI expression values from the Smart-seq3 libraries^6^ and the fraction of allele-sensitive reads were used to assign the UMI counts to the CAST or C57 allele, respectively. Cells having UMIs but lacking allelic read counts for individual genes were assigned as missing values for the inference whereas cells lacking UMIs and allelic information were considered as ‘true’ zeros and included in the analysis. The allelic expression level per cell was provided as input to the maximum likelihood inference (https://github.com/sandberg-lab/txburst); instead of using profile likelihood to estimate CIs, we performed 1,000 bootstraps per gene and allele and collected the inferred burst frequency and size of each sampled input, and importantly, each new bootstrap used a random initialization of kinetic parameter to ensure proper sampling of kinetic space. We continued with 95% CIs based on the bootstrapped parameters. For the downstream analyses we required that each gene had: **(**1) ≥1 UMI count in ≥5 cells; (2) burst size within 0.2 < size < 50; (3) burst frequency 0.01 < frequency < 30; (4) UMI expression 0.01 < UMI_mean_ < 100; and (5) width of CIs (CI_High_/CI_Low_) below 10^1.5^ (for burst size and frequency). Finally, only non-imprinted autosomal genes, identified as independent transcriptional units, were considered for downstream analysis.

### Permutation test of bursting kinetics for lncRNAs with highly ranked CV^2^

The CV^2^ for each lncRNA was ranked to 100 mRNAs of similar mean expression (using allele-distributed UMIs, equally distributed with 50 mRNAs with higher or lower expression). The top 50 ranked lncRNAs, for each individual allele, were used for downstream analysis of bursting kinetics where each lncRNA was matched with 10 mRNAs of similar expression followed by subsampling 1 expression-matched mRNA for each lncRNA (similar as for Fig. 1f). The *P* values represent the frequency where lncRNAs (median) was higher (for burst frequencies) or lower (for burst sizes) than the burst parameters for sampled mRNAs (median).

### Identification of cell cycle stage of individual primary fibroblasts

The most variable genes were identified using the R package Seurat^27^ v.4.0.5. Genes were first filtered for being expressed in ≥5 cells (≥5 read counts). Counts were normalized using LogNormalize (setting scale.factor = 10,000) and the most variable genes were identified using the vst method of FindVariableFeatures. We next extracted the cell cycle-related genes reported by Whitfield et al.^28^ (Supplementary Table 3) and used the top 50 ranked genes with the highest variability for PCA. The cell cycle phase of individual cells was identified using the first three principal components as input for the R package princurve v.2.1.6 and the Lambda factor used to align cells to the cell cycle. Expression of individual genes was illustrated using a rolling mean of 15 cells (using the R package zoo v.1.8.9). The assignment of cells to cell cycle phase was performed based on the expression levels of known cell cycle regulators (*Gas1*, *Ccne2*, *Ccnb1* and *Ccnd1*) using the rolling mean of Seurat-normalized read counts.

### Differential expression of lncRNAs in the cell cycle

Differential expression analysis between cell cycle phases (G0, G1, G1/S and G2/M) was performed using a one-way ANOVA (Benjamini–Hochberg-adjusted, *P* < 0.01) with normalized read counts (log-normalized, Seurat).

### Correlation of cell cycle genes

Genes were first filtered for being expressed in ≥2 cells (≥5 read counts). Seurat was used to log-normalize the read counts and the normalized counts were used to calculate the Spearman correlation of cell cycle genes^28^. For each pairwise comparison, cells lacking expression of both genes were excluded from the analysis.

### Cell cycle analysis

NIH/3T3 cells were washed twice in PBS and treated either with 0.1% FBS, 2 mM thymidine or 800 nM nocodazole for 16–24 h. Cells were collected using TrypLE Express, washed in PBS, resuspended in 70% ethanol and stored at −20 °C. For analysis, cells were washed in PBS and resuspended in 500 µl staining buffer (PBS containing 40 µg ml^−1^ PI, 100 µg ml^−1^ RNase A, 0.1% Triton X-100), incubated on ice for approximately 1 h and analyzed by flow cytometry. The same conditions were used for analysis on RT–qPCR.

### Identification of apoptosis-related lncRNAs

Cells assigned to the G1 cell cycle phase were extracted; fitting to the squared coefficients of variations against the means of normalized gene expressions (reads per kilobase million (RPKM)) was performed using the R function glmgam.fit() (similar to the method presented by Brennecke et al.^49^). The cell-to-cell variability of genes was ranked and the top 75 apoptosis-related genes (GO:0043065) were used for PCA. Cell clusters were identified using the pam function of the R package cluster v.2.1.2.

### RT–qPCR

RNA was extracted (QIAGEN RNeasy Mini Kit) followed by DNase treatment (Ambion DNA-free DNA Removal Kit). Equal amounts of DNase-treated RNA was used to prepare cDNA (SuperScript II or Maxima H Minus RT; Thermo Fisher Scientific) and oligo(dT)_18_ primer according to the manufacturer’s recommendations. Quantification was carried out with Power SYBR Green Master Mix (Thermo Fisher Scientific) on a StepOnePlus or ViiA 7 Real-Time PCR System (Applied Biosystems). The Delta-Delta Ct method was used to quantify relative expression levels (normalized to siControl/ASOControl treatments and *Beta-actin* unless stated otherwise). Sequences for oligonucleotides are provided in Supplementary Table 5a. Samples were required to have similar RNA content (on DNase treatment) and similar Ct values of the *Beta-actin* internal control (on RT–qPCR) to be included in the analysis.

### Cloning and generation of lentiviral U6 expressed shRNAs

Single-stranded oligonucleotides with Nhe1/Pac1 overhangs (synthesized by Integrated DNA Technologies; Supplementary Table 5) were phosphorylated (T4 Polynucleotide Kinase; New England Biolabs), linearized (95 °C for 3 min on a PCR cycler) and annealed by slowly decreasing the temperature on the PCR cycler. The previously generated pHIV7-IMPDH2-U6 construct^50^ was digested by Nhe1/Pac1 restriction enzymes, dephosphorylated (Antarctic Phosphatase; New England Biolabs) and gel-purified (QIAquick Gel Extraction Kit). The annealed oligonucleotides were ligated into the Nhe1/Pac1 and the digested pHIV7-IMPDH2-U6 construct (T4 DNA Ligase; Thermo Fisher Scientific); integration of shRNAs was verified by colony PCR and Sanger sequencing (Eurofins Genomics).

### Lentiviral stable cell lines

HEK293FT cells were transfected with pCHGP-2, pCMV-G pCMV-rev and pHIV7-IMPDH2-U6 (refs. ^50,51^) at a 1:0.5:0.25:1.5 ratio using Lipofectamine 2000 and PLUS Reagent (Thermo Fisher Scientific) in serum-depleted DMEM medium. Medium was changed approximately 6 h post-transfection to DMEM containing 10% FBS, 1% penicillin/streptomycin, 1% NEAA, 1% sodium pyruvate, 2 mM L-glutamine, 0.37% sodium bicarbonate (supplements purchased from Thermo Fisher Scientific) and 1× Viral Boost Reagent (Alstem Cell Advancements). The viral supernatant was collected approximately 48 h post-transfection, passed through a 0.45-µm filter (Sarstedt) and concentrated with PEG-it (System Biosciences) according to the manufacturer’s recommendations. NIH/3T3 cells were transduced using a low titer of lentiviral particles (<10% of transduced cells) and green fluorescent protein^+^ cells sorted at the CMB Core Facility (Karolinska Institutet).

### Colony formation assay

For stable NIH/3T3 cell lines, cells were seeded at 500 cells per well (6-well plates). After 10–14 d, cells were washed in PBS, stained for 20 min with 0.5% Crystal Violet, washed in water and left to dry. For quantification, stained cells were resolubilized in 10% acetic acid solution and then the absorbance was measured.

For siRNAs, NIH/3T3 cells were seeded at 1,000–5,000 cells per well in 6-well plates. Transfection was carried out 24 h after seeding and the procedure described above was repeated.

### siRNA and ASO knockdown

NIH/3T3 and primary cells were transfected using Lipofectamine RNAiMAX Reagent (Thermo Fisher Scientific) according to the manufacturer’s protocol. A final concentration of 10 nM siRNA and 10 nM ASO was used. Cells were transfected the day after seeding and sorted (for Smart-seq3) or RNA-extracted (for RT–qPCR) 72 h after transfection. Sequences, company names and catalog numbers for siRNAs and ASOs are provided in Supplementary Table 5b.

### PI-annexin V staining

PI-annexin V staining was carried out using the Annexin-V-FLUOS Staining Kit (catalog no. 11858777001; Roche) according to the manufacturer’s protocol. MMC treatment was initiated 24 h after siRNA transfection and samples were analyzed on a BD FACSMelody Cell Sorter 48 h later.

### Functional prediction of lncRNAs using allelic imbalance

Genes were first filtered for (1) ≥3 allelic read counts in ≥20 cells, (2) not imprinted, (3) not encoded on the X chromosome and (4) having one of the following Ensembl BioMart annotations (GRCm38.p6, Supplementary Table 2): protein_coding; lncRNA; pseudogenes; transcribed_processed_pseudogene; transcribed_unitary_pseudogene; unitary_pseudogene; unprocessed_pseudogene; and transcribed_unprocessed_pseudogene.

Allelic imbalance of gene expression was measured as defined previously: (CAST_allelicCounts_ / (CAST_allelicCounts_ + C57_allelicCounts_) – 0.5). The allelic score (allelicImbalance_lncRNA_ + allelicImbalance_mRNA_ – diff(allelicImbalance_lncRNA_, allelicImbalance_mRNA_)) was calculated for each lncRNA-mRNA gene pair within 500 kb of the lncRNA TSS. The allelic score of the lncRNA-mRNA gene pairs was compared to a permutation test where each lncRNA (*n* = 542) was moved to 1,000 randomly selected mRNA gene positions. (The 1,000 genomic loci were kept the same for all lncRNAs and required to have at least 2 other genes in proximity.) The allelic score was computed for each lncRNA-mRNA gene pair over the randomly selected genomic loci (within ±500 kb pairs (kbp)) and *P* values were calculated as: allelicScore_lncRNA:mRNA:random_ ≥ allelicScore_lncRNA:mRNA:real_ / *n*_randomGeneInteractions_.

### Functional prediction of lncRNAs using allele-resolved RNA expression

Coordinated allelic expression of lncRNA-mRNA gene pairs (at the single-cell level) was addressed for all lncRNA-mRNA gene pairs within ±500kb of the lncRNA TSS (*n* = 542 lncRNAs). The expression pattern for each gene pair (≥3 allelic read counts) was evaluated using Fisher’s exact test (*P*_Real_, Benjamini–Hochberg-adjusted). To estimate the background, each lncRNA was translocated to 1,000 randomly selected gene locations and a Fisher’s exact test applied for all randomly generated gene pairs (*P*_Random_, Benjamini–Hochberg-adjusted). lncRNA-mRNA gene pairs were considered significant if *P*_Real_ < 0.01 where *P*_Random_ < *P*_Real_ occurred in less than 1% of the permutated gene interactions.

### Estimation of RNA half-lives and decay rates

Primary mouse tail fibroblast explants (F1 offspring from one adult female CAST × C57 and one adult female C57BL6, both in technical duplicates) were treated with actinomycin D (catalog no. SBR00013-1ml; Sigma-Aldrich) at a final concentration of 5 µG ml^−1^ in quadruplicate. RNA was extracted and global levels of RNA measured by poly(A)^+^ RNA-seq. Briefly, approximately 60 ng of DNase-treated RNA was prepared for sequencing using the Smart-seq2 protocol (modified for bulk RNA-seq) and sequenced on an Illumina NextSeq 500 (High-Output Kit v.2.5, 75 cycles). Data were processed using the zUMIs v.2.9.3e pipeline and genes filtered for ≥10 read counts in all 4 samples in the untreated condition (t_0_). Using RPKMs, gene expression was first normalized to the untreated condition (setting t_0_ = 1) for each individual sample. To normalize expression over the actinomycin D-treated time points, we took advantage of previous estimates of RNA half-lives in mouse embryonic stem cells^22^. We identified a subset of control genes with half-life estimates 1 h < t_1/2_ < 8 h with ≥50 read counts at t_0_ in all 4 actinomycin D-treated samples. The expected expression level of the control genes was calculated (y = 1 × exp(−k_control:_ × t)) and used to compute a ‘normalization factor’ (by taking the median) for each time point and sample, to which all genes were normalized to reach the final relative expression levels. Genes with shorter half-lives than 2 h were excluded from the 7 h and 10 h time points when calculating the ‘normalization factor’.

To estimate the half-lives, the normalized expression was fitted to an exponential decay curve (y = a × exp(−kx)) using the R package drc v.3.0.1. The decay rate (*λ*) was calculated using the formula: t_1/2_ = ln(2) / *λ*. Genes with half-lives <10 h and burst duration <72 h were considered for downstream analysis.

### Statistical test for burst inference

To test the hypothesis regarding changes in burst kinetics, we used the likelihood ratio test. The test statistic for this test is essentially the difference between the likelihood of the null hypothesis (no change) and the likelihood of the observed change. Expressed as a formula, it is:

$$\lambda _{\mathrm{LR}} = - 2\left[ {l\left( {\theta _0} \right) - l\left( {\hat \theta } \right)} \right]$$

Where *λ*_LR_ is the likelihood ratio test statistic, *l*(*θ*_0_) is the maximal log-likelihood where the null hypothesis is true, and $l\left( {\hat \theta } \right)$ is the log-likelihood of the maximized likelihood function (that is, the observed change). According to Wilk’s theorem, *λ*_LR_ converges asymptotically to the chi-squared distribution under the null hypothesis. This enables hypothesis testing of burst kinetics by comparing *λ*_LR_ to the chi-squared distribution with 1 d.f. At *α* = 0.05, the critical value is 3.84 for a one-sided test and 7.68 for a two-sided test.

In the context of burst kinetics, we focused on the log-ratio between, for example, burst frequency in the two samples. We set the null hypothesis *θ*_0_ = 0 and the alternative hypothesis $\hat \theta = \log _2\frac{{k_{on_2}}}{{k_{on_1}}}$ where *k*_*on*1_and *k*_*on*2_ are the maximum likelihood estimates for both samples, respectively.

### Simulations of burst inference

Simulations of burst inference were used to estimate the spread in inferred kinetics to be expected, given that the observed changes in expression were only caused by changed burst frequency or size, respectively. To evaluate the spread of changed burst frequency, we first modified the burst frequency by the observed change in mean RNA expression (assuming it is 100% explained by frequency); then, we simulated RNA count observations from the Beta-Poisson model (that is, the two-state model) with the same number of cells as present in the experiment. Then, we inferred the kinetic parameters; the densities of inferred parameters were shown as clouds in the ‘burst kinetics parameter space’. The rationale is that an alteration exclusively caused by any of the parameters would be expected to occur in these subsets of space, to guide intuition and further support the hypothesis testing performed above.

### Statistics and reproducibility

No statistical method was used to predetermine sample size. The experiments were not randomized. The investigators were not blinded to allocation during the experiments and outcome assessment.

### Reporting Summary

Further information on research design is available in the Nature Research Reporting Summary linked to this article.

## Online content

Any methods, additional references, Nature Research reporting summaries, source data, extended data, supplementary information, acknowledgements, peer review information; details of author contributions and competing interests; and statements of data and code availability are available at 10.1038/s41588-022-01014-1.

## Supplementary information

Supplementary Information Supplementary Note discussing the gene expression estimates using the Smart-seq2 and Smart-seq3 protocols.

Reporting Summary

Peer Review Information

Supplementary Table 1 Supplementary Tables 1–9.

## Data Availability

The count tables used for the analysis have been made available at https://github.com/sandberg-lab/lncRNAs_bursting. The HEK293 (Smart-seq3) and mouse embryonic stem cell (Smart-seq2) data underlying the analysis of Extended Data Fig. 2 were downloaded from ArrayExpress (E-MTAB-8735, generated by Hagemann-Jensen et al.^6^) and GEO (GSE75790, generated by ﻿Ziegenhain et al.^19^), respectively. The Smart-seq3 data underlying the analysis of Fig. 2 have been deposited at ArrayExpress (E-MTAB-10148) and are also part of a previous study by Larsson et al.^47^). The previously generated Smart-seq2 data underlying the analysis for Figs. 1, 3, 5 and 7 have been deposited at the GEO (GSE75659, generated by Reinius et al.^20^). The additional Smart-Seq2 and Smart-seq3 data generated within this study have been deposited at ArrayExpress (E-MTAB-11054).

## Code availability

The R code used to reproduce and plot the major findings has been made available at https://github.com/sandberg-lab/lncRNAs_bursting and 10.5281/zenodo.5713263.
