# Supplementary material for: Transcriptional kinetics and molecular functions of long noncoding RNAs
Source: Nat Genet. 2022 Mar 3;54(3):306–17. doi: 10.1038/s41588-022-01014-1 (PMC8920890; doi:10.1038/s41588-022-01014-1)
Supplement: Supplementary file 1 — Supplementary Note discussing the gene expression estimates using the Smart-seq2 and Smart-seq3 protocols. [file 41588_2022_1014_MOESM1_ESM.pdf]

nature portfolio

https://doi.org/10.1038/s41588-022-01014-1

Supplementary information

Transcriptional kinetics and molecular functions of long noncoding RNAs

In the format provided by the authors and unedited

Supplementary Note

The Smart-seq2 protocol lacks incorporation of UMIs and we therefore used normalized read counts (RPKMs) to estimate gene expression. While read-counts and UMIs highly correlate, their exact relationship for gene expression levels is unclear. It should be noted that Smart-seq3 combines full-length transcriptome coverage with a 5' unique UMI for RNA counting and our in-house generated Smart-seq3 libraries generally contain ~50% UMI fragments of total read counts that can be used to count RNA molecules. Because of the low lncRNA expression levels, we opted to stick with read-based quantification to leverage information obtained from all reads and avoid increasing the needed sequencing depth. In contrast, allele-resolved UMI counts is important to infer accurate transcriptional bursting parameters and the Smart-seq3 data was therefore used for bursting inference.
