# Supplementary material for: Transcriptional kinetics and molecular functions of long noncoding RNAs
Source: Nat Genet. 2022 Mar 3;54(3):306–17. doi: 10.1038/s41588-022-01014-1 (PMC8920890; doi:10.1038/s41588-022-01014-1)
Supplement: Supplementary file 2 — Reporting Summary [file 41588_2022_1014_MOESM2_ESM.pdf]

nature portfolio

Corresponding author(s): Rickard Sandberg

Last updated by author(s): Oct 4, 2021

Reporting Summary

Nature Portfolio wishes to improve the reproducibility of the work that we publish. This form provides structure for consistency and transparency in reporting. For further information on Nature Portfolio policies, see our Editorial Policies and the Editorial Policy Checklist.

Statistics

For all statistical analyses, confirm that the following items are present in the figure legend, table legend, main text, or Methods section.

n/a	Confirmed	
☐	☒	The exact sample size (*n*) for each experimental group/condition, given as a discrete number and unit of measurement
☐	☒	A statement on whether measurements were taken from distinct samples or whether the same sample was measured repeatedly
☐	☒	The statistical test(s) used AND whether they are one- or two-sided<br>*Only common tests should be described solely by name; describe more complex techniques in the Methods section.*
☐	☒	A description of all covariates tested
☐	☐	A description of any assumptions or corrections, such as tests of normality and adjustment for multiple comparisons
☐	☒	A full description of the statistical parameters including central tendency (e.g. means) or other basic estimates (e.g. regression coefficient) AND variation (e.g. standard deviation) or associated estimates of uncertainty (e.g. confidence intervals)
☐	☒	For null hypothesis testing, the test statistic (e.g. *F*, *t*, *r*) with confidence intervals, effect sizes, degrees of freedom and *P* value noted<br>*Give P values as exact values whenever suitable.*
☒	☐	For Bayesian analysis, information on the choice of priors and Markov chain Monte Carlo settings
☒	☐	For hierarchical and complex designs, identification of the appropriate level for tests and full reporting of outcomes
☐	☒	Estimates of effect sizes (e.g. Cohen's *d*, Pearson's *r*), indicating how they were calculated

Our web collection on statistics for biologists contains articles on many of the points above.

Software and code

Policy information about availability of computer code

Data collection	We used a BD FACSMelody to sort cells (FACS software BC FACSChorus 1.3). Libraries were sequenced on an Illumina HiSeq2000 (for Smart-seq2 libraries), an Illumina Nextseq-500 (for RNA decay rates, High output kit v2.5, 75 cycles) and a MGI DNBSEQ-G400 (PE100 for Smart-seq3 libraries with siRNA-induced knockdown of lncRNAs). Preparation of single-cell RNA-sequencing libraries and sequencing was performed in house.
Data analysis	Custom code, preprocessed data and scripts generated within this project is available at Github (https://github.com/sandberg-lab/lncRNAs-bursting). Data analysis was preformed using R (v4.0.4 and v3.6.3) with the following packages: dplyr (v1.0.6). tidyr (v1.1.3), ggplot2 (v3.3.3), scales (v1.1.1), ggpubr (v0.4.0), zoo (v1.8.9), parallel (v4.0.4), viridis (v0.5.1), scde (v2.18.0).<br><br>The zUMIs pipeline (v2.7.0a, v2.7.1b, v2.7.2a, v2.9.3e and v2.9.4b) was used for mappping RNA-sequencing data, gene quantification and allelic calling.

For manuscripts utilizing custom algorithms or software that are central to the research but not yet described in published literature, software must be made available to editors and reviewers. We strongly encourage code deposition in a community repository (e.g. GitHub). See the Nature Portfolio guidelines for submitting code & software for further information.

Data

Policy information about availability of data

All manuscripts must include a data availability statement. This statement should provide the following information, where applicable:

- Accession codes, unique identifiers, or web links for publicly available datasets
- A description of any restrictions on data availability
- For clinical datasets or third party data, please ensure that the statement adheres to our policy

Preprocessed data (post quality control) underlaying the analysis have been made available at https://github.com/sandberg-lab/lncRNAs_bursting/data. All sequencing data has been deposited in ArrayExpress at the European Bioinformatics Institute (EBI) (ID E-MTAB-11054).

A subset of cells analyzed in this study are part of previously published studies and corresponding accession codes to public available datasets have been specified.

Field-specific reporting

Please select the one below that is the best fit for your research. If you are not sure, read the appropriate sections before making your selection.

☒ Life sciences ☐ Behavioural & social sciences ☐ Ecological, evolutionary & environmental sciences

For a reference copy of the document with all sections, see nature.com/documents/nr-reporting-summary-flat.pdf

Life sciences study design

All studies must disclose on these points even when the disclosure is negative.

Sample size	Sample sizes were not predetermined using statistical analysis but normally contained hundreds of cells. Due to the cost of preparing single-cell libraries and provide enough sequencing depth for allele-sensisive scRNA-seq, a few hundreds of cells are generally within our limitation.
Data exclusions	Single-cell RNA-seq data were filtered according to established criteria for removing technically failed cells. Cutoffs are listed where appropriate, and involved reads per cell, number of genes detected per cells and allelic distribution of read counts . qRTPCR samples were filter using the internal control (beta-actin) and RNA concentration after DNase treatment. Quality controls are described in the manuscript.
Replication	All single-cell RNA-seq experiments were performed across hundreds of individual cells. qRTPCR and colony formation experiments were performed on multiple (>3) biologically independent samples.
Randomization	Not relevant because FACS sorting of individual cells into random wells of microplates.
Blinding	Investigators were not blinded to groups of samples as they were required to guide and design the experiments and analysis.

Reporting for specific materials, systems and methods

We require information from authors about some types of materials, experimental systems and methods used in many studies. Here, indicate whether each material, system or method listed is relevant to your study. If you are not sure if a list item applies to your research, read the appropriate section before selecting a response.

Materials & experimental systems

n/a	Involved in the study	
☒	☐	Antibodies
☐	☒	Eukaryotic cell lines
☒	☐	Palaeontology and archaeology
☐	☒	Animals and other organisms
☒	☐	Human research participants
☒	☐	Clinical data
☒	☐	Dual use research of concern

Methods

n/a	Involved in the study	
☒	☐	ChIP-seq
☒	☐	Flow cytometry
☒	☐	MRI-based neuroimaging

Eukaryotic cell lines

Policy information about cell lines

Cell line source(s)	ATCC CTR-1658
Authentication	No further authentication of NIH3T3 cell line was performed. Gene expression patterns (i.e. the lack of Cdkn2a) confirmed the origin of the cell line.

Mycoplasma contamination	NIH3T3 cells were confirmed free of mycoplasma upon arrival (GATC, Eurofins). Subclones of stable shRNA-transduced NIH3T3 cell lines were not further tested for mycoplasma. Primary explants (tail fibroblasts) was not tested for mycoplasma.
Commonly misidentified lines (See ICLAC register)	No commonly misidentified cell line was used.

Animals and other organisms

Policy information about studies involving animals; ARRIVE guidelines recommended for reporting animal research

Laboratory animals	Mouse F1 offspring of CAST/EiJ X C57/Bl6J crosses were used in this study. All F1 mice were 10 weeks or older and we used both male and female mice. The humidity was set to 45%, temperature 21 +/- 1 °C and the dark/light cycle is 12 h light from 06.00 to 18.00 and 12 h darkness with dusk and dawn periods in between.
Wild animals	This study did not involve any wild animals.
Field-collected samples	This study did not involve field-collected samples.
Ethics oversight	The research carried out in this study has been approved by the Swedish Board of Agriculture, Jordbruksverket: N343/12.

Note that full information on the approval of the study protocol must also be provided in the manuscript.
