# Supplementary material for: Transcriptional kinetics and molecular functions of long noncoding RNAs
Source: Nat Genet. 2022 Mar 3;54(3):306–17. doi: 10.1038/s41588-022-01014-1 (PMC8920890; doi:10.1038/s41588-022-01014-1)
Supplement: Supplementary file 3 — Peer Review Information [file 41588_2022_1014_MOESM3_ESM.pdf]

natureresearch

Peer Review Information

Journal: Nature Genetics
Manuscript Title: Transcriptional kinetics and molecular functions of long non-coding RNAs
Corresponding author name(s): Professor Rickard Sandberg

Reviewer Comments & Decisions:

Decision Letter, initial version:

8th Jan 2021

Dear Professor Sandberg,

Your Article, "Transcriptional kinetics and molecular functions of long non-coding RNAs" has now been seen by 3 referees. You will see from their comments copied below that while they find your work of considerable potential interest, they have raised quite substantial concerns that must be addressed. In light of these comments, we cannot accept the manuscript for publication, but would be very

interested in considering a revised version that addresses these serious concerns.

In brief, the three referees present a range of assessments of your manuscript.

Reviewer #1 thought the single-cell analysis is technically sound, but has substantial criticisms of the experimental validation, asking for additional methods (Gapmers, ASOs, CRISPR) and cell line(s) to be used.

Reviewer #2 is very positive, and thinks this is a very important paper. They ask whether a known/expected cis-regulatory relationship appears in the analysis.

Reviewer #3 is more equivocal. They, as with reviewer #1, have criticisms of the of the experimental validation, and ask for greater detail on how candidates were chosen, to avoid impressions of "cherry picking".

We note that all three referees acknowledge the value of the single-cell data and the high quality of the analysis performed; however, we found the criticisms of the experimental validation to be a major concern. We agree with reviewers #1 and #3 that this aspect of the work must be improved, and believe that their suggestions are in line with common practice in the field. We additionally think that improving the characterisation of your candidates would be important to address those reviewers' concerns regarding the degree of novel, mechanistic insight into lncRNA function.

We hope you will find the referees' comments useful as you decide how to proceed. If you wish to submit a substantially revised manuscript, please bear in mind that we will be reluctant to approach the referees again in the absence of major revisions.

To guide the scope of the revisions, the editors discuss the referee reports in detail within the team, including with the chief editor, with a view to identifying key priorities that should be addressed in revision and sometimes overruling referee requests that are deemed beyond the scope of the current study. We hope that you will find the prioritised set of referee points to be useful when revising your study. Please do not hesitate to get in touch if you would like to discuss these issues further.

If you choose to revise your manuscript taking into account all reviewer and editor comments, please highlight all changes in the manuscript text file. At this stage we will need you to upload a copy of the manuscript in MS Word .docx or similar editable format.

We are committed to providing a fair and constructive peer-review process. Do not hesitate to contact us if there are specific requests from the reviewers that you believe are technically impossible or unlikely to yield a meaningful outcome.

If revising your manuscript:

*1) Include a “Response to referees” document detailing, point-by-point, how you addressed each referee comment. If no action was taken to address a point, you must provide a compelling argument. This response will be sent back to the referees along with the revised manuscript.

*2) If you have not done so already please begin to revise your manuscript so that it conforms to our Article format instructions, available <a

natureresearch

href="http://www.nature.com/ng/authors/article_types/index.html">here.
Refer also to any guidelines provided in this letter.

*3) Include a revised version of any required Reporting Summary:
https://www.nature.com/documents/nr-reporting-summary.pdf
It will be available to referees (and, potentially, statisticians) to aid in their evaluation if the manuscript goes back for peer review.
A revised checklist is essential for re-review of the paper.

Please be aware of our guidelines on digital image standards.

You may use the link below to submit your revised manuscript and related files:

[REDACTED]

Note: This URL links to your confidential home page and associated information about manuscripts you may have submitted, or that you are reviewing for us. If you wish to forward this email to co-authors, please delete the link to your homepage.

If you wish to submit a suitably revised manuscript we would hope to receive it within 6 months. If you cannot send it within this time, please let us know. We will be happy to consider your revision so long as nothing similar has been accepted for publication at Nature Genetics or published elsewhere. Should your manuscript be substantially delayed without notifying us in advance and your article is eventually published, the received date would be that of the revised, not the original, version.

Please do not hesitate to contact me if you have any questions or would like to discuss the required revisions further.

Nature Genetics is committed to improving transparency in authorship. As part of our efforts in this direction, we are now requesting that all authors identified as 'corresponding author' on published papers create and link their Open Researcher and Contributor Identifier (ORCID) with their account on the Manuscript Tracking System (MTS), prior to acceptance. ORCID helps the scientific community achieve unambiguous attribution of all scholarly contributions. You can create and link your ORCID from the home page of the MTS by clicking on 'Modify my Springer Nature account'. For more information please visit please visit <a
href="http://www.springernature.com/orcid">www.springernature.com/orcid.

Thank you for the opportunity to review your work.

Sincerely,

Michael Fletcher, PhD
Associate Editor, Nature Genetics

ORCID: 0000-0003-1589-7087

Referee expertise:

Referee #1: lncRNAs, computational biology.

Referee #2: lncRNAs

Referee #3: lncRNAs, single-cell genomics.

Reviewers' Comments:

Reviewer #1:
Remarks to the Author:
In “Transcriptional kinetics and molecular functions of long non-coding RNAs”, the authors exploit an allele-sensitive, single-cell RNA sequencing methodology to dissect transcriptional dynamics and regulatory functions of long non-coding RNAs (lncRNAs). Taking advantage of the single-cell resolution, they smartly demonstrate lncRNAs as a group have distinct transcriptional kinetics compared to mRNAs, that is, have lower burst frequencies, have burst sizes that are either only slightly lower or even higher, eventually resulting in increased cell-to-cell variability. By examining asynchronously growing cells, the authors further identified lncRNAs with roles in cell cycle progression and programmed cell death, and provided a preliminary experimental validation. Lastly, by combining allele and single cell approaches they pinpoint specific lncRNAs that regulate the expression levels and bursting parameters of target genes found in cis.

Taken together, the authors demonstrate the power of single-cell RNA sequencing combined to allele-specific analyses in identifying and uncovering the putative functions of long non-coding RNAs. In particular, they show that some lncRNAs regulate expression levels of other RNAs found in cis to their loci by altering their burst sizes and other times by affecting frequencies, although there isn’t a clear trend or a mechanistic model for the differences between lncRNAs that affect different aspects of gene regulation. The manuscript is very well written and the analyses are generally rigorous and well presented. Overall, from the perspective of the lncRNA field, there are two nice observations about the burst dynamics and molecular phenotypes for several lncRNAs related to cell cycle progression and apoptosis (these lncRNAs join the ranks of many other lncRNAs suggested to regulate these processes, including in recent high-throughput screens in fibroblasts done by the FANTOM consortium). The authors make the case that the scRNA-seq data are useful for finding functionally important lncRNAs, but at present it is difficult to gauge to what extent the rate of discovery of functional lncRNAs here is really much better than focusing on conserved, or broadly or highly expressed lncRNAs. Beyond those observations, the novelty for lncRNA field is limited. Description of lncRNA expression levels and variability across cells have been already described at the single-cell level (10.1186/s13059-016-0932-1), and as mentioned there are already numerous lncRNAs is proposed functions in proliferation (but unknown modes of action).

Major Comment
1. The description and validation of the proposed functions in regulating cell proliferation and control of neighboring genes are very superficial compared to the current standard in the field. It is appreciated that this is not the main focus of the manuscript, but it is not sufficient to show that RNAi of the lncRNAs leads to effects on proliferation, as those may result from potential off-target effects. If the lncRNAs that regulate the cell cycle indeed have trans effects, can the authors show that the

phenotype can be rescued/reversed using ectopic RNA expression? Are these observed when the lncRNAs are perturbed using Gapmer/ASOs or CRIPSR interference? Moreover, adding another, unrelated mouse cell line in which experimental validation is carried on may provide a broader relevance of the considered lncRNAs, as well as potentially uncovering additional functions. It is not necessarily required to find the mode of action or to validate all the tested lncRNAs, or to use multiple cell lines for all experiments, but at present the analysis of lncRNA functions is very superficial. Additional experiments will be able to discriminate between cis and trans effects, as well as help dissect which one between transcriptional activity at lncRNA locus or the lncRNA molecule itself is the mediator of the phenotypes considered.

Minor comments
1. Binomial distributions applied to allele-specific expression (ASE) are prone to false positives, given the easiness of rejecting the null hypothesis combined with the below-average sequencing depth of heterozygous sites which may lead to higher allelic imbalances. Other approaches employ beta-binomial distributions (10.1038/nature08872, 10.1093/bioinformatics/btu802, 10.7554/eLife.33480.001), among other methods, which may be further expanded by including pseudo-counts to avoid excluding conditions in which one allele is nearly or not expressed at all. Therefore, repeating the relevant analyses by identifying ASE by modeling allelic counts with a different statistical distribution and then comparing the results across methods may strengthen the results and provide more biologically-relevant examples.
2. The paper begins with analysis in Figure 1 of adult tail fibroblasts using Smart-seq2 (which to my understanding does not include UMIs, and so it is difficult to go from read counts to RNA molecules in this method, which should be mentioned/discussed). The authors then switch in Figure 2 to another dataset obtained using Smart-seq3. Do the results shown in Figure 1 still hold in this second dataset? If so, why not use the Smart-seq3 dataset for those analyses as well? This should be at least discussed.

Reviewer #2:
Remarks to the Author:
The study entitled "Transcriptional kinetics and molecular functions of long non-coding RNAs" is tor de force of, allele-specific, RNA imaging towards understanding patterns and potential functions of lncRNAs. The authors use tail fibroblasts derived from a hybrid mouse line in order to develop allele-specific RNA-fish probes owing to the evolutionary distance between the two strains. The authors first perform RNA-seq to determine the repitoire of genes that can be detected in an allele, and single-cell, specific manner -- across a large cohort of 500+ fibroblast lines. The authors find that as a whole lncRNAs are more heterogenously expressed than mRNAs and yet sub-sampling of equal expression and smaller sets of lncRNAs results in the previous finding that lncRNAs and mRNAs have similar distributions of per cell expression. Next the authors explore "bursting" rates relative to expression and as expected these two parameters are correlated. Interestingly, lncRNAs have slower bursting rates that could explain overall lower abundance. Taking this a step further the authors investigate decay rates and find, as other studies have, that lncRNA and mRNA decay rates are unlikely to account for the lower abundance of lncRNAs. This detailed analysis converges at an important finding that highly variable lncRNAs are more likely to have larger bursts relative to more homogeneously expressed lncRNAs.

The authors next look at antisense and bidirectional lncRNAs expression properties -- to my

knowledge this has not yet been explored globally and is a very interesting aspect of this study. The authors find that bidirectional promoters result in increased bursting -- with out increased product -- and thus are more abundant in this orientation. The authors continue to use single cell analysis, combined with cell-cycle markers to identify those lncRNAs with "cell-specific" expression patterns resulting a candidate list of ~120 lncRNAs -- that are used to explore the dynamics of this cell-specific expression. Seven of these candidates were validated by qRT-PCR.

Heroically, the authors continue to determine the functional role, if any, of these lncRNAs in cell cycle progression using "synchronized" NIH 3T3 cells. Indeed 3 of the three tested using shRNA knockdown showed an increase or decrease in cell cycle progression (including the previously published LOCKD locus). The authors find that the RNA product (owing to shRNA KD) resulted in the phenotype, where as previously LOCKD genetic deletion only affect expression of the upstream CDKN1b RNA and no other genes. The authors find that depletion of the RNA product did not affect CDKN1b, as would be expected with an intact enhancer element embedded in the LOCKD DNA locus. This is actually quite important for another reason: recent advances have found siRNA, shRNA and ASO depletions can result in defective PAS recognition and thus results in epigentic silencing of the locus. If this were the case the authors would have observed CDKN1b being depleted -- as the enhancer would eventually be silenced. Yet unlike the previous study they found ~700 genes that were influenced upon depletion of the LOCKD RNA product. A similar strategy was carried out for the WINCR1 lncRNA and several candidates "associated by guilt" with DNA damage response.

Finally, the authors harness the power of skewed expression between alleles in hybrid mouse lines as established (Andergassen et al.). Using a very clever approach the authors look for lncRNA-mRNA pairs on specific alleles to see how they are correlated in expression "in cis". Importantly the authors employed a permutation analysis to ensure observed affects are not due to position in the genome -- it is well established that proximal genes will correlate more than distal genes. So this empirical null distribution is a very classy way to normalize these potential positional affects.

Overall, this study sheds a wonderful new insight into the transcriptional dynamics of lncRNAs that underly many of the known properties that have been observed, yet not understood on "how" these properties of lncRNAs could arise. I am very impressed with the authors command of statistics, often using empirical distributions, permutation analyses that are absolutely essential in these studies and if not done properly can result in very misleading results from the same data. At every step the authors measure background and find those "extremophiles" that stand out and in turn functionally validate their observations. I had to read this manuscript several times, not out of concern, rather to learn more. In short I have one suggestion for the authors below. Other than that I am very grateful for the authors not only in the scale, rigor and integrative approaches, but the refreshingly statistical based findings that are consistently tested by LOF studies.

In short, I congratulate the authors on such impressive diligence on a first submission! I am confident that this study, as is, will be of great interest to the broad readership of Nature Genetics.

1) It has been published that "Hottip" correlates with the expression of HOXA9-13 (which should be expressed in NIH 3T3 cells as they encode a "distal hox expression pattern, with a binary switch between off (HoxA1-A7) and on HOXA9-13. Although the authors have done a rigorous statistical analysis to derive allele based correlations -- I am surprised this interaction was not noticed as it is

well defined to occur in cis. Can the auhtors use this example as a "benchmark" in their analyses to determine where a known cis-proximal allele regulation occurs? It maybe that the candidates studied here are more dramatic examples, but I would expect this to come out of their analyses (even if not allelic skewed).

Reviewer #3:
Remarks to the Author:
The authors find that lncRNAs, compared with mRNAs, tend to be expressed at lower burst frequencies with larger burst sizes. This new finding is welcome because it sheds light on lncRNA transcriptional processes. Subsequently, the authors seek to use single cell allelic imbalances of transcription to associate the expression of a lncRNA with a chromosomally neighbouring protein coding gene. This is technically challenging because lncRNAs tend to be lowly expressed and C57/Cast-informative SNPs are relatively rare. Nevertheless, some evidence of "coordinated" (in cis) coexpression was found, which would be consistent with previous observations of transcriptional rippling (Ebisuya et al. Nature Cell Biol 10, 1106). Whether such coordination has a consequential effect on cellular phenotypes (i.e. "is functional") is not investigated or determined. Technically, the single cell and allelic imbalance experiments and analyses are performed to a high level. Nevertheless, the shRNA experiments could have been affected by substantial off-target effects, but these were not addressed. Finally, at critical points in the authors' narrative it appears that subjective decisions were taken to select lncRNAs and experiments (i.e. "cherry-picking"). Greater clarity and transparency over these issues would be welcome.

Three major issues:
1. p10 Figure 3D. Was shRNA KD of the 4 other lncRNAs attempted, and if so what were the results? Acknowledging that shRNAs often have dramatic off-target effects, the findings would be more robust if results for all 7 were shown (and with shRNA duplicates) and, given the variation, with replicate numbers greater than 3 or 4. A more definitive experiment (e.g. Figure 4A; 4D; 4E; 4F; 4G; 5E) would show that shRNA-mediated effects are absent when the designed target site is removed. In the main text clarify that a significance effect on fibroblasts was observed with siLockd-1 – currently this effect is obscured by the vague phrase "no consistent change". The possibility of off-target effects is briefly mooted (bottom of p11) and, indeed, it does look from Fig S6D that among the 752 genes more are reduced in expression than increased, although we are not told this. Indeed, the effects of shRNAs could be transcriptional (rather than post-transcriptional) (e.g. Kalantari et al. NAR 2016), and alternative explanations of the authors' shRNA data are currently lacking in their manuscript.
2. Cherry-picking. Greater rigour when justifying the selection of various lncRNAs or experiments is essential.
3. Figure 6. The authors define lncRNAs as being "cis-functioning" if they are co-expressed with their adjacent pc gene in an allele-dependent manner. Nevertheless, this conflates expression with function: the lncRNA may not have any selected effect function (see Doolittle WF et al. GBE 2014) and might be a by-product of the pc gene's transcription. Panels L/M: these 95% CI are overlapping so I remain unconvinced that there are effects on burst size/frequency. Results should be shown for both c57 and Cast. Panels N/O: these refer to simulations that – together with their statistical tests - are not described adequately. It is not clear to me that these two panels provide biological insight.

Other issues:
Figure 1H Y axis label: typo: "permutaions".

Page 6: "against 100 expression matched mRNAs" (delete 's')
Throughout: For exponents use base 10: i.e. "<1x10-4" not "<1e-4". This causes confusion, especially in Figure 6C where "e" could be confused as Euler's number.
Figure 2H/I Y axis label: "Occurrence".
p7 "possibly regulated by enhancer activities20,28–30" is cryptic and needs clarification.
p8 Explain more clearly that the "50 most variable lncRNAs on each allele (ranked CV2)" were chosen also according to mean expression.
p9 Figure S4M: in the main text explain that the "increase in expression" was approximately 50%. Similarly (Figure 2L) explain that the burst size increase is ~ +50%.
p9 "Asynchronously growing mouse fibroblasts (Figures S1A-C)" – is this Figure S5A-C, instead?
p10 "of which we selected seven highly ranked candidates for further characterization". Please clarify: were these the highest ranked lncRNAs (ranked on p-value?), and how were they distributed among the cell cycle phases?
p12 "This approach strongly reduced the number of candidate genes" – please provide further information regarding q-value thresholds for the Spearman pairwise correlations. Figure 4C: Aside from previous data in the literature, why were 3 kinesins cherry-picked from among 138 genes? Are the other 135 genes also involved in mitosis, or not?
p12 What was the specific rationale for investigating Wincr1 further?
p13 Figure S7B and "coordinated expression". Such "rippling effects" have been known for over 10 years (Ebisuya et al. Nature Cell Biol 10, 1106) and are not necessarily reflective of coordinated effects with cellular consequences.
p13 Typo: "mechansisms". Figure 4F: the possibility that Wincr1 maintains other trans-acting functions is of interest, but the data would also be consistent with expected off-target effects. Bottom of p13, state that you used GO for the genes related to apoptotic signalling. Figure 5A: it is good practice to provide the %age of variance explained by each PC.
p14 Figures 5B and S8B. The "focusing specifically" on a "cluster of cells that expressed genes involved in stress signalling" looks again like cherry-picking, especially when only two genes (Gadd45b and Cdkn1a) are exemplifications of this gene annotation category. At this point, therefore, the reference to "stress signalling" is not merited, and should be removed. Figure 5C: were these the only 5 lncRNAs? If not, then why were these selected? re: "scaled with the concentration of MMC (Figure 5D)" – statistical support (if any) for this statement is required. Why were 3 of 5 lncRNAs chosen, and on what basis?
p15 Using exactly what criteria were the 4 "highly ranked lncRNA-mRNA interactions" selected? Were siRNAs designed for other targets but are not discussed because of null results?
p17 Can you please quantify the "good agreement" between the Smart-seq3 and qRTPCR results?
p23 The code for these processing steps should be made freely available.
Also, it would be of interest to know whether lncRNA burst size/frequency vary by whether the lncRNA has a single or else multiple exons.

Signed: Chris Ponting

Author Rebuttal to Initial comments

Reviewer #1
In "Transcriptional kinetics and molecular functions of long non-coding RNAs", the authors exploit an allele-sensitive, single-cell RNA sequencing methodology to dissect transcriptional dynamics and regulatory functions of long non-coding RNAs (lncRNAs). Taking advantage of the single-cell resolution, they smartly demonstrate lncRNAs as a group have distinct transcriptional kinetics compared to mRNAs, that is, have lower burst frequencies, have burst sizes that are either only slightly lower or even higher, eventually resulting in increased cell-to-cell variability. By examining asynchronously growing cells, the authors further identified lncRNAs with roles in cell cycle progression and programmed cell death, and provided a preliminary experimental validation. Lastly, by combining allele and single cell approaches they pinpoint specific lncRNAs that regulate the expression levels and bursting parameters of target genes found *in cis*.

Taken together, the authors demonstrate the power of single-cell RNA sequencing combined to allele-specific analyses in identifying and uncovering the putative functions of long non-coding RNAs. In particular, they show that some lncRNAs regulate expression levels of other RNAs found *in cis* to their loci by altering their burst sizes and other times by affecting frequencies, although there isn't a clear trend or a mechanistic model for the differences between lncRNAs that affect different aspects of gene regulation. The manuscript is very well written and the analyses are generally rigorous and well presented. Overall, from the perspective of the lncRNA field, there are two nice observations about the burst dynamics and molecular phenotypes for several lncRNAs related to cell cycle progression and apoptosis (these lncRNAs join the ranks of many other lncRNAs suggested to regulate these processes, including in recent high-throughput screens in fibroblasts done by the FANTOM consortium). The authors make the case that the scRNA-seq data are useful for finding functionally important lncRNAs, but at present it is difficult to gauge to what extent the rate of discovery of functional lncRNAs here is really much better than focusing on conserved, or broadly or highly expressed lncRNAs. Beyond those observations, the novelty for lncRNA field is limited. Description of lncRNA expression levels and variability across cells have been already described at the single-cell level (10.1186/s13059-016-0932-1), and as mentioned there are already numerous lncRNAs is proposed functions in proliferation (but unknown modes of action).

We appreciate the insightful comments and welcome that the reviewer values the novel aspect of transcriptional bursting of lncRNAs. We are very grateful for the reviewer's input we think that the manuscript has improved significantly after taking the comments and suggestions into account.

We agree with the reviewer that conservation of lncRNAs as a criterion for functional evaluation is a valuable, although not useful for the many lncRNAs that lack clear conservation (i.e. reviewed by Johnsson et al, 10.1016/j.bbagen.2013.10.035). Moreover, simply using average expression levels as a selection criterion would also be inefficient. For example, many protein-coding transcription factors (TFs) are not among the most highly expressed genes (**Reviewers Figure 1a**, pasted below), yet still fundamental for gene regulation. Importantly, the computational and experimental

approaches presented in our study is effective for lncRNAs at various average expression levels (**Reviewers Figure 1b**, pasted below). In summary, we believe that other strategies, beyond expression levels and conservation, are deeply needed.

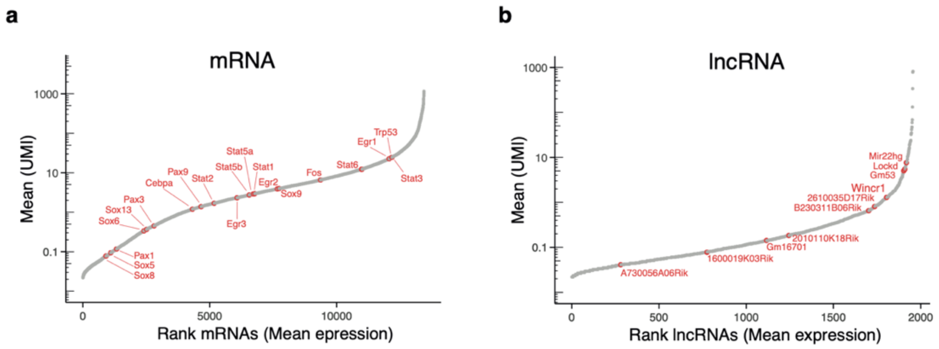

Reviewers Figure 1. (a) Protein-coding genes ranked by their average expression levels. A subset of manually selected transcription factors are highlighted in red. (**B)** lncRNA genes ranked by their average expression levels. LncRNAs that are evaluated as part of our study are highlighted in red.

The reviewer highlights a previous study (*Liu et al,* Genome Biology 2016, 10.1186/s13059-016-0932-1) that studied the expression and variability of lncRNA expression levels in individual cells. This study used Fluidigm C1 to generate 226 single-cell transcriptomes during neocortical development that they classify into 7 cell types. They emphasize that individual cells can have high expression of lncRNAs relative to tissue level RNA-seq which was generated from a heterogenous sample containing a multitude of cell types. It would therefore be expected to find individual cells with relatively high expression of lncRNAs, that most likely merely represent cell type specific expression, that become heavily diluted in the tissue-level RNA-seq. Importantly, the authors do not account for the well-established cell type specific expression pattern of lncRNAs in their analysis. Thus, Liu et al. reports mainly on differential expression of lncRNAs within the seven cell types, although without any modeling of the heterogeneity within cell types. We also note that the study unfortunately lacks any attempt to evaluate and compare cell-to-cell heterogeneity between lncRNAs and mRNAs, in a manner that account for their difference in mean expression levels. As we show in this study, such comparisons are deceiving as they are confounded by the expected variability differences associated with the difference in expression levels. Although the study by *Liu et al* adds important information about lncRNA expression levels during neocortical development, it adds less insights into cell-to-cell heterogeneity of lncRNAs within cell types. Rather, most of their analysis mirror previous bulk-experiments and capture well-known tissue specific expression patterns of lncRNAs.

In our study, we present evidence that lncRNAs have higher cell-to-cell variability than mRNAs expressed at similar mean expression levels. Furthermore, we link the cell-to-cell variability to a shift in transcriptional burst parameters and find lncRNAs to have low burst frequency and high burst size, compared to mRNAs of similar average expression levels. We explore the concept to use single-cell heterogeneity and link lncRNA expression profiles to distinct cellular states. We show that linking lncRNA expression profiles to such transient states provides important information about

how, and where, to carry our functional characterization of lncRNAs. Notably, we show that both *cis*- and *trans*-functioning lncRNAs can be identified. With the increasing amount of scRNA-seq data being publicly available, we believe that our study provides important insights for the scientific community and will help to improve functional characterization of lncRNAs.

Major Comments

1. The description and validation of the proposed functions in regulating cell proliferation and control of neighboring genes are very superficial compared to the current standard in the field. It is appreciated that this is not the main focus of the manuscript, but it is not sufficient to show that RNAi of the lncRNAs leads to effects on proliferation, as those may result from potential off-target effects. If the lncRNAs that regulate the cell cycle indeed have trans effects, can the authors show that the phenotype can be rescued/reversed using ectopic RNA expression? Are these observed when the lncRNAs are perturbed using Gapmer/ASOs or CRIPSR interference? Moreover, adding another, unrelated mouse cell line in which experimental validation is carried on may provide a broader relevance of the considered lncRNAs, as well as potentially uncovering additional functions. It is not necessarily required to find the mode of action or to validate all the tested lncRNAs, or to use multiple cell lines for all experiments, but at present the analysis of lncRNA functions is very superficial. Additional experiments will be able to discriminate between *cis* and *trans* effects, as well as help dissect which one between transcriptional activity at lncRNA locus or the lncRNA molecule itself is the mediator of the phenotypes considered.

The reviewer raises several important points. We agree with the reviewer that more comprehensive validation of the candidate lncRNAs was needed. Still, we want to emphasize that multiple siRNAs had been assessed, and stable shRNA lentiviral transduced cell lines generated for a subset of candidates. As suggested by the reviewer, we have made a comprehensive effort to target candidate lncRNAs using 32 ASOs. In line with previous reports (i.e., *Ramilowski J et al*, where 879 of 2021 ASOs generated a modest 40-60% knock-down), only a handful of the 32 generated ASOs reached efficient knockdown (**Reviewers Figure 2,** pasted below), and we below validate core aspects of our study using some of these ASOs.

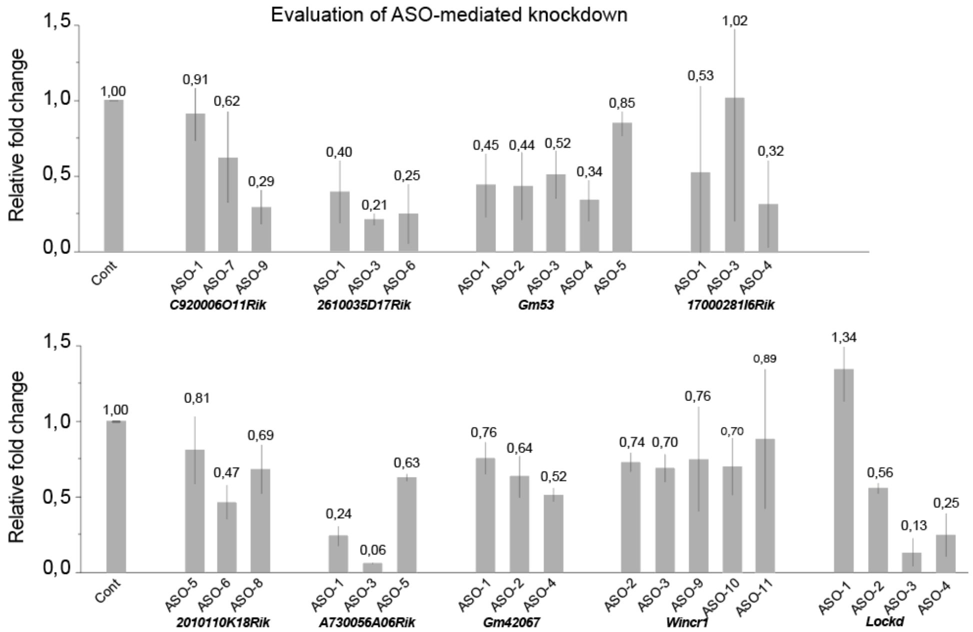

Reviewers Figure 2. qRTPCR measurements upon ASO-mediated knockdown of nine lncRNAs. The ASOs were designed using the Qiagen online algorithm.

Cell cycle associated lncRNAs, related to figures 3 and 4 in manuscript

We initially reported an effect on colony formation upon knockdown of the cell cycle associated lncRNAs *Wincr1*, *Lockd* and *A730056A06Rik*. Further, *Wincr1* was found to regulate several genes in *cis* ($p19^{arf}$, $p16^{ink4a}$ and $p15^{ink4b}$) while we suggested *Lockd* to function in *trans,* mainly through its regulation of the *Kif11* and *Kif14* genes.

Lockd: We evaluated the effect of one ASO (the one with most efficient knockdown of the 4 ASOs that was assessed) and confirmed the effect on colony forming cells as well as *Kif14*. These new data have been added to **Figure 4A** (colony formation), **Figure S6C** (validation of knockdown) and **Figure S6F** (the effect on *Kif14* upon ASO induced knockdown of *Lockd*). These new data support our previous findings using siRNAs.

Wincr1: Of the 5 ASOs targeting *Wincr1* at modest knockdowns (**Reviewers Figure 2,** pasted above), we evaluated the effect of *Wincr1-ASO3* in greater detail. The modest knockdown of *Wincr1* by *Wincr1-ASO3* significantly induced the expression of $p19^{arf}$, $p16^{ink4a}$ and $p15^{ink4b}$, (**Reviewers Figure 3**, pasted below), thus validating the siRNA-mediated knockdown effects reported in initial submission. These new data have been added as **Figures S7E** of the revised manuscript. Noteworthy, the induction of $p19^{arf}$, $p16^{ink4a}$ and $p15^{ink4b}$ scaled with the knockdown (**Figures 4G** and **S7E** in manuscript) where *Wincr1-ASO3* (~40% knockdown) had a relatively modest effect compared to siRNA-induced knockdown (~75% knockdown). This was also illustrated on colony formation where ASO induced knockdown did not have any effect, likely representing the incomplete knockdown using the ASO (added as **Figures S7F** of the revised manuscript). The ASO-mediated knockdown of *Wincr1* has been introduced on page 14 of the revised manuscript and the data is presented as **Figure S7E-F.**

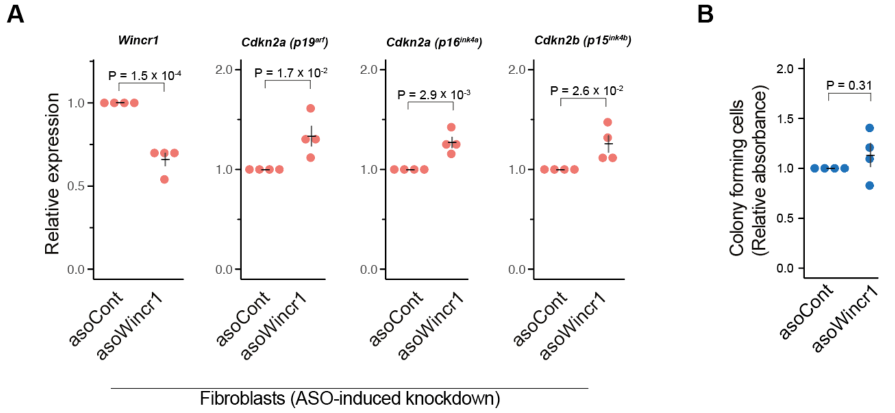

Reviewers Figure 3. (A) qRTPCR measurement upon ASO-mediated knockdown of *Wincr1* in primary fibroblasts. **(B)** Quantification of colony forming cells upon ASO induced knockdown of *Wincr1*.

<u>*A730056A06Rik:*</u> We identified two ASOs with good knockdown of *A730056A06Rik* (**Reviewers Figures 2** and **5**). Detailed analysis of *A730056A06Rik* upon revision identified this lncRNA to be an antisense transcript to the cell cycle regulatory gene *Rgma*. ASO induced knockdown combined with FBS starvation (in NIH3T3 cells) exposed *A730056A06Rik* to function as a Natural Antisense Transcript (*cis*-NAT) to *Rgma,* where knockdown of *A730056A06Rik* attenuated the induction *Rgma* *(***Reviewers Figure 4 A-C**, pasted below**)**. In contrast to the *A730056A06Rik* lentiviral stable cell line, a decrease in colony forming cells was observed upon ASO induced knockdown (**Reviewers Figure 4 D,** pasted above). The ASO-mediated knockdown of *A730056A06Rik* has been added into to the revised manuscript (**Figures 4H-K**) and these new data are being discussing on page 14 of the revised manuscript.

"In summary, our ASO mediated knockdown support A730056A06Rik to function as a cis-NAT, while the effects on colony formation remains inconclusive and need further evaluation. We speculate that these disparities could relate to shRNA off target effects, their different modes of knockdown (to target spliced or unspliced transcripts), or potentially long-term induced compensatory effects (of shRNAs) versus the short-term induced knockdown (of ASOs)."

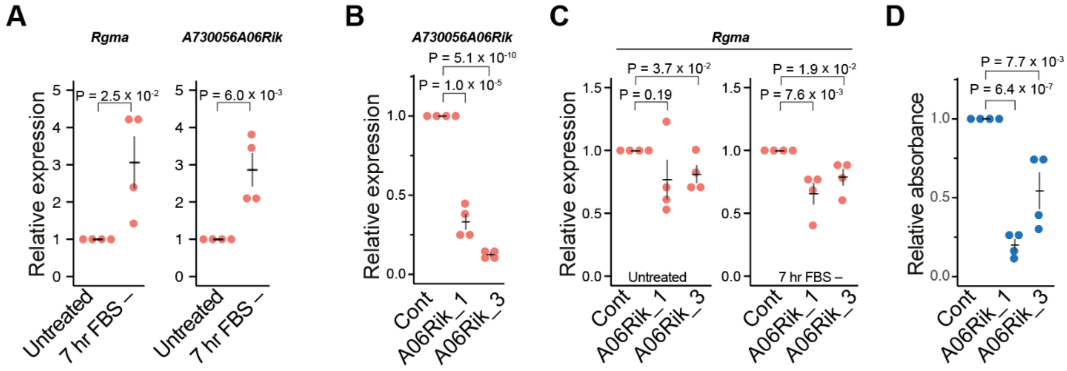

Reviewers Figure 4. (A) Relative expression of *Rgma* and *A730056A06Rik* measured by qRTPCR upon 7-hour serum starvation in NIH3T3 cells. **(B)** Relative expression of *A730056A06Rik* measured by qRTPCR upon ASO induced knockdown in NIH3T3 cells. **(C)** Relative expression of *Rgma* measured by qRTPCR upon serum starvation and ASO induced knockdown of *A730056A06Rik.* **(D)** Quantification of colony forming cells upon ASO induced knockdown of *A730056A06Rik* in NIH3T3 cells.

Cis-functioning lncRNA-mRNA gene pairs, related to figure 6 in manuscript

In addition to evaluating cell cycle associated lncRNAs with ASOs, we generated ASOs against two lncRNAs with *cis*-regulatory effects: *1700028I16Rik* (acting on *Txnrd1)* and *2610035D17Rik* (acting on *Sox9*). After ASO induced knockdown for each of these two lncRNAs (using the most efficient ASOs identified in **Reviewers Figure 2**, pasted above), we observed increased expression levels of *Txnrd1* and *Sox9* (**Reviewers Figure 6,** pasted below). This experiment validates the siRNA-mediated effects reported in the initial manuscript, and these new data has been added to **Figure S10B** and **S10E** in the revised manuscript (data is being introduced on page 17).

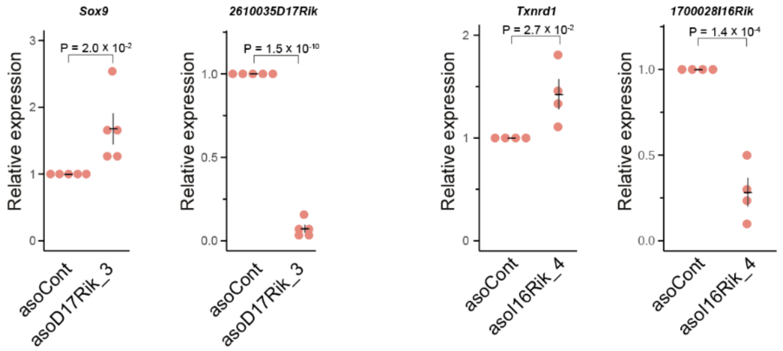

Reviewers Figure 6. qRTPCR measurements of *cis*-functioning lncRNA:mRNA gene pairs validated by ASO induced knockdown.

As suggested by the reviewer, we also evaluated the possibility of using CRISPR-Cas9 (dCas9a). We generated 'mini-pools' of gRNAs (4 gRNAs targeting the same lncRNA-promoter) for a total of 11 lncRNAs (and 4 gRNA controls) and cloned a total of 48 individual gRNAs. Upon plasmid DNA transfections (using the transfection Lipofectamine LTX reagent, supposed to be *'a plasmid transfection reagent suitable for challenging cell types such as primary fibroblasts'*) very minor/no effect was observed of lncRNA expression levels in NIH3T3 cells (**Reviewers Figure 7A**, pasted below). A subset of gRNA-pools was also evaluated in primary fibroblast with similar observations as in NIH3T3 cells (**Reviewers Figure 7B**, pasted below). Plasmid transfection of NIH3T3 and primary fibroblasts is challenging, and we believe that the lack of induction is primarily due to low transfection efficacy. It should be noted that the Cas9 enzyme is large, and the vector used for these experiments (pAC154-dual-dCas9-VP160) relatively large (~8.8 kb), therefore not making it optimal for highly efficient transfections. We have also seen that transfection of plasmids (in our hands) quite severely effect proliferation and apoptosis and we therefore decided not to pursue these experiments any further.

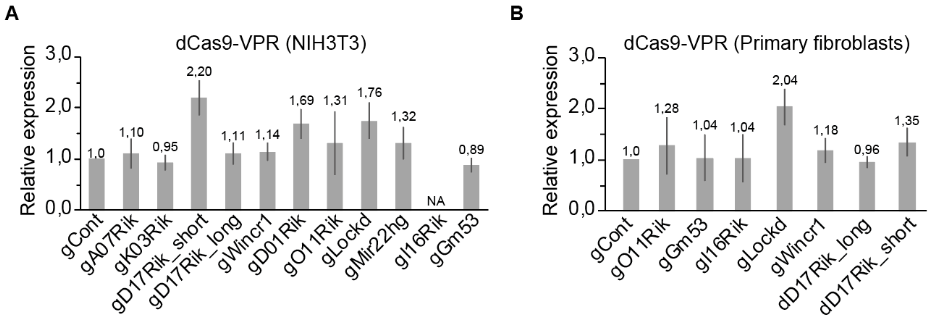

Reviewers Figure 7. qPCR-measurement of CRISPR-dCas9a targeted lncRNAs in **(A)** NIH3T3 cells and **(B)** primary fibroblasts.

The reviewer also points out that a broader relevance of the lncRNA effects can be demonstrated if the effects were observed in multiple cell lines. We agree with this statement and in the initial submission we demonstrated the effects of *Lockd* and *Wincr1* in both primary tail fibroblasts and the immortalized embryonic fibroblast NIH3T3 cell line. Although not explicitly discussed in the manuscript, we generally observed a greater effect when targeting cell cycle related lncRNAs in primary fibroblast compared to NIH3T3, possibly reflecting that the immortalized NIH3T3 cells might have less controlled cell cycle and fewer intact cell cycle check points. In the revised manuscript, we have put more emphasis on the fact that certain lncRNAs were investigated in two different cell lines and now write (on page 10, new text is underlined) *"To functionally evaluate the selected lncRNA candidates' potential involvement in cell cycle progression, <u>we used an independent cell line</u>, the immortalized mouse embryonic fibroblast NIH3T3 cell line."*. We also want to emphasize that many lncRNAs have highly restricted expression (e.g. cell or tissue-specific) and it could be challenging to find multiple established model systems having the lncRNA of interest expressed. Out of interest, we explored the expression pattern of lncRNA-*Wincr1* using the FANTOM Zenbu browser and quite interestingly noticed that expression of *Wincr1* seems restricted to **1)** mouse fibroblast cells and **2)** Tracheal epithelial cells differentiated into ciliated epithelial cells (**Reviewers Figure 8** in response letter). We believe this quite clearly illustrates that it is not always straight forward to find multiple model systems for characterization of lncRNAs.

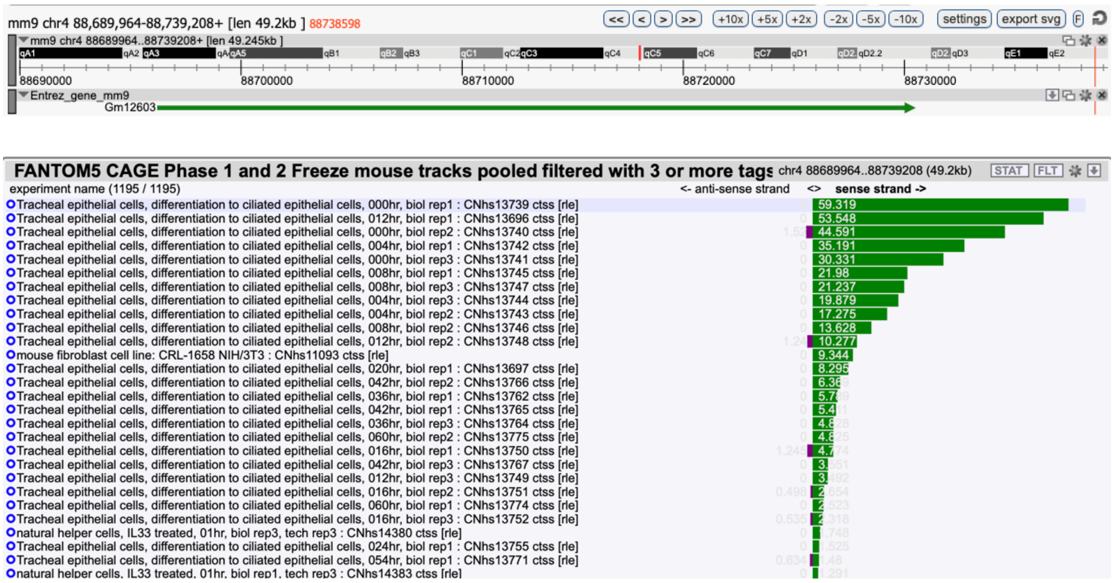

Figure 8 Screen shot from FANTOM5 Zenbu 3.0 browser representing Cage-tags of lncRNA-*Wincr1* (on the sense strand) in green (*Wincr1* here annotated as *Gm12603*).

The reviewer also raises the possibility of dissecting whether the effects of a lncRNA is mediated by the transcriptional activity itself or the effects of the resulting RNA. This is a highly important and debated area of lncRNA research, however outside the scope of our present study. To shed light on that issue typically require a whole study on a single lncRNA and not feasible to the list of candidates reported in our present study which demonstrated important general aspects of lncRNA cell-to-cell variability that

we relate to specific transcriptional bursting patterns. We further show how variable expression even within a cell type can lead to strong functional predictions that we validate with multiple knockdown strategies. On this topic, our favored approach to target lncRNAs have been siRNAs since recent work have raised the concern that ASOs may induce premature termination of transcription and cannot always be used to study RNA-mediated functions of lncRNA loci (Mol Cell 2020, 10.1016/j.molcel.2019.12.011). To our knowledge, no such observations have been reported for siRNAs.

Altogether, in the revised manuscript we have validated both cell cycle effects and *cis*-regulatory effects of lncRNAs using ASOs. These new experiments were added as part of **Figure 4**, **Supplementary Figures 7** and **10** of the revised manuscript. We thank the reviewer for this suggestion and the inclusion of these experiments have strengthened our work further.

Minor comments

1. Binomial distributions applied to allele-specific expression (ASE) are prone to false positives, given the easiness of rejecting the null hypothesis combined with the below-average sequencing depth of heterozygous sites which may lead to higher allelic imbalances. Other approaches employ beta-binomial distributions (10.1038/nature08872, 10.1093/bioinformatics/btu802, 10.7554/eLife.33480.001), among other methods, which may be further expanded by including pseudo-counts to avoid excluding conditions in which one allele is nearly or not expressed at all. Therefore, repeating the relevant analyses by identifying ASE by modeling allelic counts with a different statistical distribution and then comparing the results across methods may strengthen the results and provide more biologically-relevant examples.

We generally agree with the reviewer's statement regarding false positives in ASE when defined by a certain distribution (typically binomial), and in particular when combined with below-average sequencing coverage. Moreover, to confidently ascertain allelic expression patterns in single cells, there is the additional challenge with the periodic nature of transcription that can lead to lowly expressed genes not being detected.

In this manuscript, we have however used a distribution agnostic permutation test to determine the statistical significance of ASE. We have further restricted this analysis to only analyze genes that had at least 3 allelic read counts in 20 cells (in the analysis of allelic imbalance). Therefore, the strategy we used was developed to directly avoid the two main challenges raised by the reviewer (defining significance by a statistical test and low sequence depth). We also refer to our reply to reviewer 2 for further discussion related to validation of allelic expression estimated from the scRNA-seq data.

From the references provided by the reviewer, 10.1093/bioinformatics/btu802 explicitly models the genotype likelihood of the bases at the heterozygous sites. This is not directly relevant to this work since we use well defined F1 crossbreeds with

deeply sequenced and highly reliable reference genomes. According to the reasons mentioned above, we are confident in our estimates of ASE.

Alternative strategies to compute ASE by comparing observed read counts to different theoretical null distributions are interesting to pursue and something that we would highly appreciate as a resource for the field (although permutation tests should always be performed to directly assess the accuracy of the distribution-based tests used on the data).

2. The paper begins with analysis in Figure 1 of adult tail fibroblasts using Smart-seq2 (which to my understanding does not include UMIs, and so it is difficult to go from read counts to RNA molecules in this method, which should be mentioned/discussed). The authors then switch in Figure 2 to another dataset obtained using Smart-seq3. Do the results shown in Figure 1 still hold in this second dataset? If so, why not use the Smart-seq3 dataset for those analyses as well? This should be at least discussed.

This is an important point that we had not clearly described in the first submission. The reviewer is correctly pointing out that Smart-seq2 data lacks UMI information for molecule counting. We repeated the analyses shown in Figure 1 on the dataset generated using Smart-seq3 and can confirm the results, adding further confidence from this independent experiment (**Reviewers Figure 9**, pasted below, left panel). While read counts and UMIs are of course highly correlated, their exact relationship for gene expression levels is unclear. It should be noted that Smart-seq3 combines full-length transcriptome coverage with a 5' unique UMI for RNA counting and our in-house generated Smart-seq3 libraries generally contain ~50% UMI fragments of total read counts that can be used to count RNA molecules. Because of the very low lncRNA expression levels, we opted to stick with read-based quantification to leverage information obtained from all reads and avoid increasing the needed sequencing depth. Notably, we would lose approximately 50% of the 'information content' by using the UMIs only. To illustrate, we have compared the number of lncRNAs detected per cell using either read counts or UMIs and see approximately 50% more lncRNAs per cell when using all read counts (lncRNA(UMI) = 137, lncRNA(read counts) = 207, median) (**Reviewers Figure 9,** pasted below, right panel). To better clarify this in the manuscript, we now discuss the difference between Smart-seq2 and Smart-seq3 in Methods (on page 28):

"The Smart-seq2 protocol lacks incorporation of UMIs and we therefore used normalized read counts (RPKMs) to estimate gene expression. While read-counts and UMIs highly correlate, their exact relationship for gene expression levels is unclear. It should be noted that Smart-seq3 combines full-length transcriptome coverage with a 5' unique UMI for RNA counting and our in-house generated Smart-seq3 libraries generally contain ~50% UMI fragments of total read counts that can be used to count RNA molecules. Because of the low lncRNA expression levels, we opted to stick with read-based quantification to leverage information obtained from all reads and avoid increasing the needed sequencing depth. In contrast, allele-resolved UMI counts is critical to resolve accurate burst parameters and the Smart-seq3 data is therefore used for bursting inference."

Another reason for using the Smart-seq2 data, was simply that the cells iteratively obtained from many different animals over several experiments, and therefore slightly more heterogenous compared to the Smart-seq3 data. This seems beneficial when exploiting cellular heterogeneity and assigning lncRNAs to transient cellular states. To highlight this difference, we now write (on page 5, new text is underlined):
-*"We also made use of a previously published data set consisting of additional 158 cells, generating a comprehensive data set of 533 deep-sequenced fibroblasts from several animals".*
Related to Smart-seq3, we now write (on page 7, new text is underlined):
-*"To do this, we generated a comprehensive data set of adult tail fibroblast using Smart-seq3 (682 cells post quality control isolated from a single animal)".*

Finally, we should also admit that the structure of the manuscript to some extent mirrors a historical perspective of our in-house development of the Smart-seq 2/3 protocols. While we quite early on found that lncRNAs have higher cell-to-cell variability (related to **Figure 1** in the manuscript) using the Smart-seq2 protocol (2014, doi:10.1126/science.1245316), we were not able to link this observation to transcriptional burst kinetics until more recently with the development of Smart-seq3 (2020, doi:10.1038/s41587-020-0497-0) and the computational encoding of transcriptional burst kinetics(2019, doi:10.1038/s41586-018-0836-1).

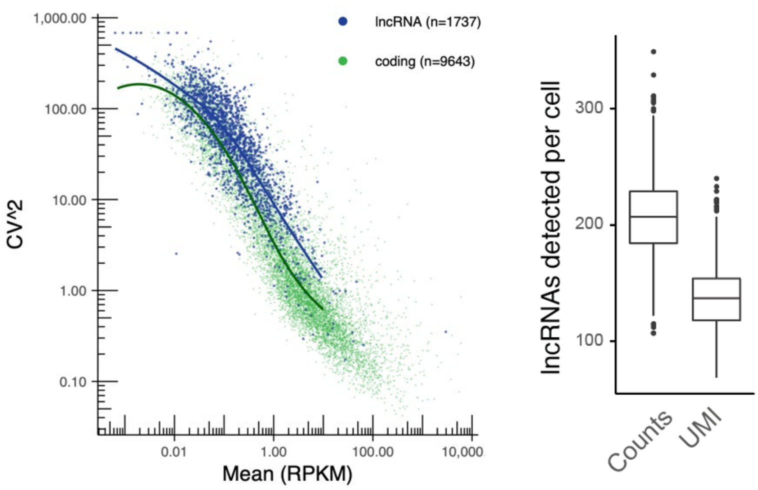

Reviewers Figure 9. **(Left panel)** Scatter plot of mean expression against the CV^2 for lncRNAs (blue) and mRNAs (green). Lines denote a smoothed fit to the rolling mean for lncRNAs and mRNAs. **(Right panel)** Number of lncRNAs being detected using either read counts or UMIs.

Reviewer #2

The study entitled "Transcriptional kinetics and molecular functions of long non-coding RNAs" is tor de force of, allele-specific, RNA imaging towards understanding patterns and potential functions of lncRNAs. The authors use tail fibroblasts derived from a hybrid mouse line in order to develop allele-specific RNA-fish probes owing to the evolutionary distance between the two strains. The authors first perform RNA-seq to determine the repertoire of genes that can be detected in an allele, and single-cell, specific manner -- across a large cohort of 500+ fibroblast lines. The authors find that as a whole lncRNAs are more heterogenously expressed than mRNAs and yet sub-sampling of equal expression and smaller sets of lncRNAs results in the previous finding that lncRNAs and mRNAs have similar distributions of per cell expression. Next the authors explore "bursting" rates relative to expression and as expected these two parameters are correlated. Interestingly, lncRNAs have slower bursting rates that could explain overall lower abundance. Taking this a step further the authors investigate decay rates and find, as other studies have, that lncRNA and mRNA decay rates are unlikely to account for the lower abundance of lncRNAs. This detailed analysis converges at an important finding that highly variable lncRNAs are more likely to have larger bursts relative to more homogeneously expressed lncRNAs.

The authors next look at antisense and bidirectional lncRNAs expression properties -- to my knowledge this has not yet been explored globally and is a very interesting aspect of this study. The authors find that bidirectional promoters result in increased bursting -- with out increased product -- and thus are more abundant in this orientation. The authors continue to use single cell analysis, combined with cell-cycle markers to identify those lncRNAs with "cell-specific" expression patterns resulting a candidate list of ~120 lncRNAs -- that are used to explore the dynamics of this cell-specific expression. Seven of these candidates were validated by qRT-PCR.

Heroically, the authors continue to determine the functional role, if any, of these lncRNAs in cell cycle progression using "synchronized" NIH 3T3 cells. Indeed 3 of the three tested using shRNA knockdown showed an increase or decrease in cell cycle progression (including the previously published LOCKD locus). The authors find that the RNA product (owing to shRNA KD) resulted in the phenotype, where as previously LOCKD genetic deletion only affect expression of the upstream CDKN1b RNA and no other genes. The authors find that depletion of the RNA product did not affect CDKN1b, as would be expected with an intact enhancer element embedded in the LOCKD DNA locus. This is actually quite important for another reason: recent advances have found siRNA, shRNA and ASO depletions can result in defective PAS recognition and thus results in epigentic silencing of the locus. If this were the case the authors would have observed CDKN1b being depleted -- as the enhancer would eventually be silenced. Yet unlike the previous study they found ~700 genes that were influenced upon depletion of the LOCKD RNA product. A similar strategy was carried out for the WINCR1 lncRNA and several candidates "associated by guilt" with DNA damage response.

Finally, the authors harness the power of skewed expression between alleles in hybrid mouse lines as established (Andergassen et al.). Using a very clever approach the authors look for lncRNA-mRNA pairs on specific alleles to see how they are correlated in expression "*in cis*". Importantly the authors employed a permutation analysis to ensure observed affects are not due to position in the genome -- it is well established that proximal genes will correlate more than distal genes. So this empirical null distribution is a very classy way to normalize these potential positional affects.

Overall, this study sheds a wonderful new insight into the transcriptional dynamics of lncRNAs that underly many of the known properties that have been observed, yet not understood on "how" these properties of lncRNAs could arise. I am very impressed with the authors command of statistics, often using empirical distributions, permutation analyses that are absolutely essential in these studies and if not done properly can result in very misleading results from the same data. At every step the authors measure background and find those "extremophiles" that stand out and in turn functionally validate their observations. I had to read this manuscript several times, not out of concern, rather to learn more. In short I have one suggestion for the authors below. Other than that I am very grateful for the authors not only in the scale, rigor and integrative approaches, but the refreshingly statistical based findings that are consistently tested by LOF studies.

In short, I congratulate the authors on such impressive diligence on a first submission! I am confident that this study, as is, will be of great interest to the broad readership of Nature Genetics.

Comment

1) It has been published that "Hottip" correlates with the expression of HOXA9-13 (which should be expressed in NIH 3T3 cells as they encode a "distal hox expression pattern, with a binary switch between off (HoxA1-A7) and on HOXA9-13. Although the authors have done a rigorous statistical analysis to derive allele based correlations -- I am surprised this interaction was not noticed as it is well defined to occur *in cis*. Can the authors use this example as a "benchmark" in their analyses to determine where a known *cis*-proximal allele regulation occurs? It maybe that the candidates studied here are more dramatic examples, but I would expect this to come out of their analyses (even if not allelic skewed).

We thank the reviewer for the enthusiastic and encouraging comments. The idea to benchmark our analyses on a validated and well-studied example is excellent and we therefore looked more carefully into the *Hottip-HoxA* locus. Unfortunately, there are no allele-informative SNPs for *Hottip in* our mouse F1 cross (although for several of the *HoxA* protein-coding genes) and the *Hottip-HoxA* interaction is therefore not included in our analysis. *Hottip* is also relatively low expressed, and it is therefore likely that more cells and deeper sequenced cells might be needed to capture this interaction. Nevertheless, without resolving the allelic expression in our single-cell data, we do observe the binary switch between *HoxA1-A7* (transcriptionally suppressed) and *HoxA9-A13* (transcriptionally active) (**Reviewers Figure 10,** pasted below).

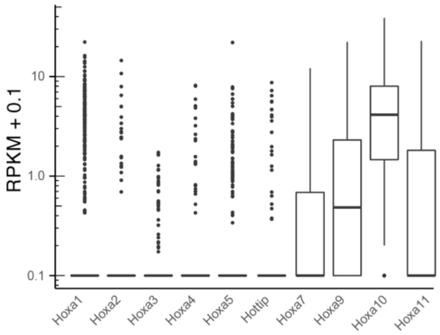

Reviewers Figure 10. Boxplots showing UMI expression levels for the *Hoxa-Hottip* locus.

Along the same lines as suggested by the reviewer, we have typically validated the power in the allelic expression estimated from the scRNA-seq data using known imprinted genes (i.e. the lncRNA-*Airn*) and X-chromosome inactivation (**Reviewers Figure 11**). In earlier studies on female embryonic cells (e.g. Deng et al. Science 2014 and Reinius et al. Nature Genetics 2016) we accurately identify Xist expression from the silenced X, whereas all other genes except escapee genes are detected from only the active X. In Reinius et al, we also showed that the scRNA-seq data had sufficient power to identify the monoallelic expression of all non-escapee genes only transcribed from active X. Moreover, the few imprinted genes expressed in the cells studied showed the expected patterns. To this end, we are generally confident that the gene-level power to identify allelic expression patterns is relatively large. These data are currently not presented in the manuscript (can be added upon request) since we consider that the allelic distribution for non-escapee genes on the X-chromosome (**Figure S1C** in manuscript) serves as good validation of our method.

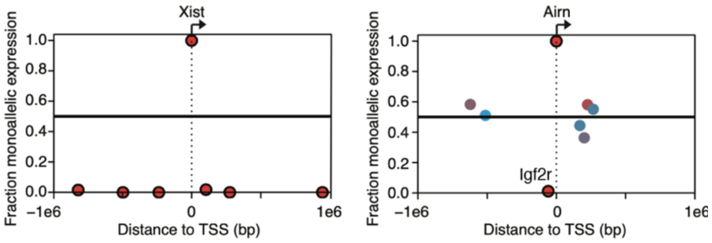

Reviewers Figure 11. (A) Allelic read count distributions of Xist (expressed on inactive Xi) and genes in proximity (expression on active Xa). **(B)** Allelic read count distributions of lncRNA-*Airn* (paternally expressed) and *Igf2r* (maternally expressed) and genes in proximity.

Reviewer #3

The authors find that lncRNAs, compared with mRNAs, tend to be expressed at lower burst frequencies with larger burst sizes. This new finding is welcome because it sheds light on lncRNA transcriptional processes. Subsequently, the authors seek to use single cell allelic imbalances of transcription to associate the expression of a lncRNA with a chromosomally neighbouring protein coding gene. This is technically challenging because lncRNAs tend to be lowly expressed and C57/Cast-informative SNPs are relatively rare. Nevertheless, some evidence of "coordinated" (*in cis*) coexpression was found, which would be consistent with previous observations of transcriptional rippling (Ebisuya et al. Nature Cell Biol 10, 1106). Whether such coordination has a consequential effect on cellular phenotypes (i.e. "is functional") is not investigated or determined. Technically, the single cell and allelic imbalance experiments and analyses are performed to a high level. Nevertheless, the shRNA experiments could have been affected by substantial off-target effects, but these were not addressed. Finally, at critical points in the authors' narrative it appears that subjective decisions were taken to select lncRNAs and experiments (i.e. "cherry-picking"). Greater clarity and transparency over these issues would be welcome.

We appreciate the comprehensive and constructive comments from reviewer. As seen in the detailed answers to the major comments below we have made a significant effort to validate effects using other knockdown strategies, that all have different off-target spectra. We have also made efforts to explain certain decisions regarding candidates studied, and to extend the analysis to more lncRNAs to demonstrate the general power of the approach. Within the response letter, we leave very comprehensive details about the decision making and strategies to select lncRNA candidates for functional validation. At present, these notes have not been added to the manuscript, but we will be happy to incorporate these points as supplementary notes upon the reviewer's guidance. Though, throughout the manuscript we now clarify the selection of candidate genes (although more briefly) and also attach several additional Supplementary Tables where the reader can easily access the data for further analysis.

The reviewer also raises the intriguing question of to what extent proximal genes in our genomes tend to be expressed simultaneously. In fact, this we have studied with each iteration of our single-cell RNA-seq data with allelic resolution. Already in Deng et al. Science 2014 we show that genes with divergent promoters (i.e. really proximal and with shared/close by promoters) do have allelic bursts more synchronous than expected by chance. However, this did not extend to close by genes without divergent promoters (although that could have been affected by the lack of power in the initial scRNA-seq data at that time). Regarding the work by *Ebisuya et al* (Nature Cell Biol 10, 1106), we think the observation is interesting, although focused on the strong induction of immediate-early genes in response to growth factor stimulation (upon 36 hours of serum starvation). Investigations of allelic expression at the level of individual transcriptional bursts across large number of cells would be likely required to reach conclusive insights on this important topic.

Three major issues:

1. a) p10 Figure 3D. Was shRNA KD of the 4 other lncRNAs attempted, and if so what were the results? Acknowledging that shRNAs often have dramatic off-target effects, the findings would be more robust if results for all 7 were shown (and with shRNA duplicates) and, given the variation, with replicate numbers greater than 3 or 4. **b)** A more definitive experiment (e.g. Figure 4A; 4D; 4E; 4F; 4G; 5E) would show that shRNA-mediated effects are absent when the designed target site is removed. **c)** In the main text clarify that a significance effect on fibroblasts was observed with siLockd-1 – currently this effect is obscured by the vague phrase "no consistent change". **d)** The possibility of off-target effects is briefly mooted (bottom of p11) and, indeed, it does look from Fig S6D that among the 752 genes more are reduced in expression than increased, although we are not told this. **e)** Indeed, the effects of shRNAs could be transcriptional (rather than post-transcriptional) (e.g. Kalantari et al. NAR 2016), and alternative explanations of the authors' shRNA data are currently lacking in their manuscript.

The reviewer raises many important questions in this paragraph, and for clarity we have answered them in pieces (denoted by **a)** to **e)** in the reviewers comment above).

1a) Our initial effort to characterize cell cycle related lncRNAs was to generate multiple cell lines for each lncRNA, however we generally did not receive effective knockdowns (lncRNA-*2010110K18Rik* was included at this stage). We used low virus titer (moi < 0.1) to generate polyclonal cell lines with single integration sites. Retrospectively, higher titer might have been more beneficial in generating stronger knockdowns, although it might have led to transcriptional silencing of the viral insert and negative selection of cells with suppression of lncRNAs. After these initial drawbacks, we instead designed siRNAs and found siRNA-mediated knockdown to be highly efficient in both primary fibroblast and NIH3T3 cells (using the Lipofectamine RNAiMax reagent). It should be noted that using siRNAs over ASOs was an active decision making since our primary interest has been to study the function of the lncRNA transcript, rather than its transcription (since recent literature suggests ASOs to trigger premature termination of transcription, 10.1016/j.molcel.2019.12.011). To overcome the general issue of siRNA induced off-target effects, we generally used 2 (or more) siRNAs for each lncRNA.

As suggested by the reviewer, we have made a comprehensive effort to target additional cell cycle related lncRNAs. We designed several siRNAs towards *Mir22hg* (4 siRNAs in total), *1600019K03Rik* (3 siRNAs in total) and *2010110K18Rik* (3 siRNAs in total) while we were unfortunately not able to find any pre-designed siRNAs towards *Gm12963*. Together with the data we presented in our initial manuscript, we now study 6 of the 7 cell cycle associated lncRNAs (**Reviewers Figure 12,** pasted below, also as **Figure 3E-F** in the revised manuscript, data being introduced on page 11). Importantly, we could demonstrate effects of 4 lncRNAs on colony formation over multiple siRNAs (*Lockd*, *Wincr1*, *Mir22hg*, *2010110K18Rik*). However, the effect on colony formation upon knockdown of *1600019K03Rik* was inconsistent between siRNAs *(**Reviewers figure 12**, pasted below, and **Figure 3E-F** in manuscript)* and the effect of targeting *A730056A06Rik* was not consistent between all experiments

(shRNA and ASO, also see the reply on page 5 to reviewer #1). Briefly, we introduce two highly efficient ASOs in the revision that were internally consistent (both reducing colony forming cells) although in disagreement with the stable lentiviral shRNA-*A730056A06Rik* transduced cell line (that increased colony formation). This motivated us to perform additional experiments on *A730056A06Rik* where we found this lncRNA to function as a cis-NAT transcript to the protein-coding gene *Rgma* (summarized in the reply to reviewer #1, page 5 in reply letter, **Reviewers figure 4**). As pointed out by the reviewer, this clearly highlights the need to use multiple siRNAs and additional strategies like ASOs for validation of lncRNA functions. We believe this likely reflects off-target effects of the stable cell line, although we cannot exclude alternative explanations (i.e, ASOs induced premature termination of transcription, differences in acute versus long term knockdown). The ASO-mediated knockdown of *A730056A06Rik* has been added into to the revised manuscript (**Figures 4H-K**) and these new data are being discussing on page 14 of the revised manuscript.
"In summary, our ASO mediated knockdown support A730056A06Rik to function as a cis-NAT, while the effects on colony formation remains inconclusive and need further evaluation. We speculate that these disparities could relate to shRNA off target effects, their different modes of knockdown (to target spliced or unspliced transcripts), or potentially long-term induced compensatory effects (of shRNAs) versus the short-term induced knockdown (of ASOs)."

Altogether, in the revision we have added data for 3 additional cell cycle associated lncRNAs, evaluated the effect of 9 siRNAs, evaluated CRISPR-dCas9a for 11 lncRNAs (**Reviewers figure 7,** not feasible in our model systems) screened 32 ASOs (**Reviewers figure 2**) and selected 6 of them for further downstream molecular characterization. These new additions were added to revision as **Supplementary figures** (S7, S10) and main **Figures 3** and **4.**

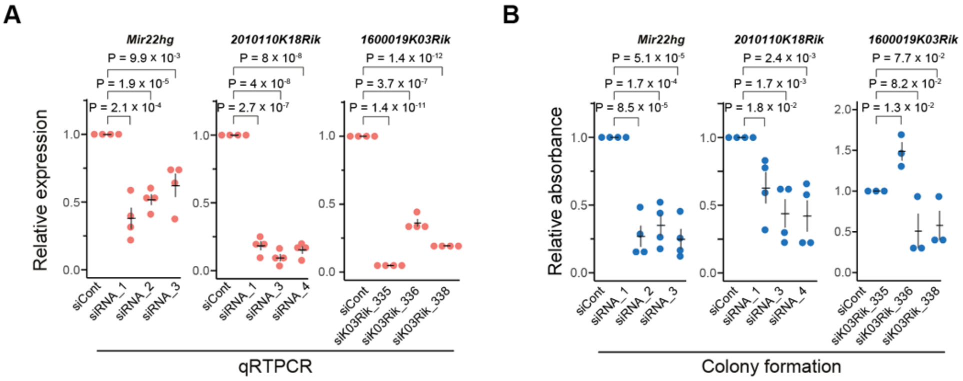

Reviewers Figure 12. (A) Relative expression of three cell cycle associated lncRNA upon siRNA knockdown measured by qRTPCR. **(B)** Quantification of colony forming cells upon siRNA induced knockdown of three cell cycle associated lncRNA.

1b) We agree with the reviewer that removing the target site is a very elegant control experiment, although not trivial for this large number of siRNAs/shRNAs that are being evaluated throughout this study (>15 in total). Additional challenges include that **i)** primary fibroblasts have a very limited time in culture before being senescent (~2 weeks), **ii)** the NIH3T3 cell line is largely tetraploid and **iii)** RNA secondary structures might be affected upon disruption of the siRNA-seed sequence. In this study, we use

multiple siRNAs, shRNAs and ASOs with different seed sequences, that together should exclude the possibility that the reported consistent phenotypes were due to off-target effects (which should be specific to each targeting sequence).

1c) This has been clarified. On page 11, at the end of the first paragraph, we now write (new text is underlined):
"In line with the previous report, no consistent change in RNA expression was observed for *Cdkn1b* upon knockdown of the *Lockd* transcript in NIH3T3 or primary fibroblast cells, though siLockd-3 induced the expression of *Cdkn1b* in primary fibroblasts (**Figure 4B**)."

1d) This is very observant by the reviewer, and we did not notice this during our analysis. The number of genes being significantly induced (292) and reduced (460) in expression has been specified in **Figure S6D** and specified in the text (on p12). Though, we are not fully sure how this relates to off-target effects? It seems likely that this can be explained by downstream effects due to suppression of *Lockd*, thus having slower proliferating cells. Most certainly, there are off-targets effects among the 752 significant genes that we try to overcome by the incorporation of Spearman correlations. However, to our understanding we would expect that off-target effects are roughly equally distributed among positively and negatively affected genes. To increase transparency and to allow readers to further explore our data, we now attach a supplementary table with all 752 significant genes (new Supplementary Table S7).

1e) The reviewer highlights a review by *Kalantari et al* (NAR 2016) discussing that shRNA/siRNAs can trigger transcriptional effects. As illustrated by Kevin Morris and colleagues (Science 2004, 10.1126/science.1101372), it was found that RNAi itself can trigger transcriptional changes in the nucleus of mammalian cells (frequently named siRNA-induced Transcription Gene Silencing, TGS, and transcriptional activation / de-repression, TGA). Subsequent studies have found that this mode of regulation requires AGO1 and/or AGO2 (Morris KV, RNA 2006 10.1261/rna.2235106; Rossi JJ, NSMB 2006 10.1038/nsmb1142; NSMB 2006, 10.1038/nsmb1140) where the siRNA-AGO complex interacts with nascent promoter overlapping RNA transcripts (PNAS 2007, 10.1073/pnas.0701635104). Throughout our studies, it has been of greatest priority to use siRNAs that are well separated from promoter regions to avoid unintended targeting/disruption of any promoter-associated transcript(s). Two siRNAs, siK14Rik_1 and siGm53_3, were directed towards the proximity of a promoter region (exon 1 of the lncRNAs). However, the effect of these lncRNAs have been confirmed with siRNAs targeting further downstream. We are therefore confident that the concerned raised by the reviewer has been avoided.

To increase justification of our findings, alternative explanations related to 1) premature termination of transcription, 2) differences in acute versus long term knockdown, 3) off-target effects and 4) TGS, have added to the discussion. The following text has been added to the discussion, page 21:
"In summary, our functional analysis covers several siRNAs, ASOs and stable lentiviral transduced cell lines, well established strategies to study loss of function of lncRNAs. However, each approach has different off-target spectra and may induce unintended

effects. For example, the effects of ASO-induced premature termination of transcription, siRNA-induced off-target effects, differences in acute (siRNAs-induced knockdown) versus long-term (stable cell lines with shRNA-induced knockdown) and siRNA-induced transcriptional gene silencing/activation, should never be overlooked."

2. *Cherry-picking. Greater rigour when justifying the selection of various lncRNAs or experiments is essential.*
(Also reply to the following minor comment):
-p10 "of which we selected seven highly ranked candidates for further characterization". Please clarify: were these the highest ranked lncRNAs (ranked on p-value?), and how were they distributed among the cell cycle phases?

We agree with the reviewer that the selection criteria were not transparent throughout the manuscript, and as specified below we have improved that considerably in the revised manuscript.

We aimed to investigate a few lncRNAs from each phase of the cell cycle (G0, G1S, G2M) and to include intergenic as well as antisense lncRNA transcripts. To reach this, we essentially used four criteria for selecting candidates, **1)** p-values (ANOVA test, Benjamini-Hochberg adjusted), **2)** fold induction, **3)** the possibility of doing downstream functional studies and **4)** literature research. For example, *Lockd* and *Mir22hg* are found among the lncRNAs with the lowest p-values while *1600019K03Rik*, *A730056A06Rik* and *2010110K18Rik* are found among the lncRNAs with the greatest fold induction within their respective cell cycle phase (with a significant adjusted p-value). We also noticed that *2010110K18Rik* and *A730056A06Rik* are Natural Antisense Transcripts (NATs) to the cell cycle related genes *Cdc25c* and *Rgma*, respectively, while *1600019K03Rik, Mir22hg, Lockd* and *Wincr1* are intergenic lncRNAs. While adjusted p-values and fold inductions served as our primary filter, the literature research, and the possibility of doing downstream functional studies narrowed down the candidates further.

While *Lockd*, *Mir22hg*, *1600019K03Rik, A730056A06Rik* and *2010110K18Rik* are among the top ranked genes (adjusted p-values and/or fold changes) it should be noted that many of the candidates with higher ranking were not suitable for downstream analysis. For example: ***C230088H06Rik*** (ranking #1 on adjusted p-value) is a 474kb long gene that overlaps 4 other genes. ***Gm34821*** (ranking #3 on adjusted p-value) is an antisense transcript that overlaps one additional gene. ***Gm26573*** (ranking #4 on adjusted p-value) is a 870kb long gene that overlaps > 5 other genes. ***Gm4961*** (ranking #5 on adjusted p-value) is likely a pseudogene to the protein coding gene *Kif2c*. Based on previous work done by the main author on the PTEN pseudogene (10.1038/nsmb.2516, and 10.1038/s41598-021-89389-9), functional investigation of pseudogenes with high sequence homology to their parental gene(s) is extremely challenging. However, we must admit that lncRNA-***Gm11867*** (ranking #2 on adjusted p-value) seems to be a suitable target for downstream analysis but was unfortunately not considered.

From the literature perspective: **1)** We found the expression pattern of *Lockd* intriguing since *Lockd* had been reported to positively regulate transcription of the negatively cell cycle regulator p27. Thus, the expression pattern of *Lockd* was non-intuitive since we observed high expression levels of *Lockd* in proliferating cells. **2)** *Wincr1* was found to be located at the same locus as the key cell cycle regulators *Cdkn2a* / *Cdkn2b* and there is a human encoded lncRNA (*MIR31HG*) that has been found to regulate *CDKN2A*. It should also be noted that only 27 lncRNAs reached our cutoff in the same category as *Wincr1* (G1S), thus making it possible to briefly evaluate the literature and availability of pre-designed siRNAs, for all candidates.

The selection of making lentiviral stable cell lines targeting *Wincr1*, *Lockd* and *A730056A06Rik* simply represent one candidate from each cell cycle phase.

Although not clear in the first submission, a systematic effort was indeed spent to select candidate genes (and to functionally validate their effect) and we hope this clarification has made the process transparent. In the revised manuscript, we have clarified the selection strategy (first section on page 10) and added a supplementary table (new **Supplementary Table 4**) with more detailed information about fold changes and p-values for all lncRNAs. We now write:
"For validation experiments, we selected at least two highly ranked candidate lncRNAs from each cell cycle phase (based on adjusted p-values and fold change inductions), excluded lncRNAs that overlapped with multiple other genes to facilitate downstream perturbation experiments, and proceeded with seven lncRNA candidates for further characterization."
We have not included the full text above (as it becomes long and anecdotal), however we would be happy to include it at full length (e.g. as a supplemental note) if the reviewer thinks that would be more appropriate.

Better justification for the making the lentiviral transduced cell lines has been added on page 10 (new text is underlined):
*"Having validated cell cycle specific expression of these lncRNA transcripts, we next generated individual lentiviral transduced NIH3T3 cell lines with stable shRNA-induced knockdown for three of the candidates (Wincr1, Lockd and A730056A06Rik, representing candidates from each cell cycle phase) to perform in-depth functional investigation (**Figure 3C**)."*

3. *Figure 6. The authors define lncRNAs as being "cis-functioning" if they are co-expressed with their adjacent pc gene in an allele-dependent manner. Nevertheless, this conflates expression with function: the lncRNA may not have any selected effect function (see Doolittle WF et al. GBE 2014) and might be a by-product of the pc gene's transcription. Panels L/M: these 95% CI are overlapping so I remain unconvinced that there are effects on burst size/frequency. Results should be shown for both c57 and Cast. Panels N/O: these refer to simulations that – together with their statistical tests - are not described adequately. It is not clear to me that these two panels provide biological insight.*

We fully agree that coordinated expression of lncRNAs and protein-coding genes should not be confounded with function. We have in the revised manuscript rephrased this part to make the distinction between a strategy to identify pairs of potential *cis*-regulatory function from experiments needed to demonstrate such a function. On page 17, second section, we now write (new text is underlined):
*"To fully take advantage of the allelic resolution, we next assessed if allele-specific expression patterns on the single-cell level for the same set of lncRNA-mRNAs gene pairs as above (5,824 gene pairs +/- 500 kb of the lncRNA TSS, **Figure 6B**), could be used as a strategy to identify pairs with potential cis-regulatory function for in depth molecular characterization."*

Whether coordinated expression of lncRNA-mRNA gene pairs, on the allelic level, provide information of *cis*-functions had not been evaluated and we here used it as a strategy to select candidates for further experimental investigation. We note that coordinated allelic expression is a much stronger selection criteria than expression level correlations within or across different tissues, and even correlation in gene expression on the single-cell level (non-allele resolved).

The reviewer brings up several aspects of the analysis of bursting kinetics after lncRNA knockdown for which we apologize since the descriptions were clearly insufficient. First, the reason for not showing both alleles in the analysis of bursting kinetics after lncRNA knockdown was simply that certain effected mRNAs were not sufficiently expressed from the other allele for us to resolve the burst parameters. As presented in figures **S9D-E**, most genes (mRNAs and lncRNAs) present allelic imbalance and we therefore have different power to infer bursting parameters for the individual alleles. It is also likely that the siRNA induced knockdown of lncRNAs have different effect on each individual allele due to the allelic imbalance. We have attached the alternative alleles within the response letter (**Reviewers Figure 13**, pasted below) but do not find these of great interest for the readers. We have in the revised manuscript instead better explained the inability to infer parameters for those alleles for which the bursting parameter could not deconvolute (on page 19, new text is underlined).
*"We next inferred burst parameters for Txnrd1, Gsta4, Sox9, Cdkn2a and Hoxb13 from the allele with the highest precision in burst inference (generally the highest expressed allele) (**Figures S9D-F**) since their allelic imbalance precluded bursting inference from both alleles, while Tmc7 and Fam78b did not reach sufficient UMI counts and SNP coverage for burst inference from either allele."*

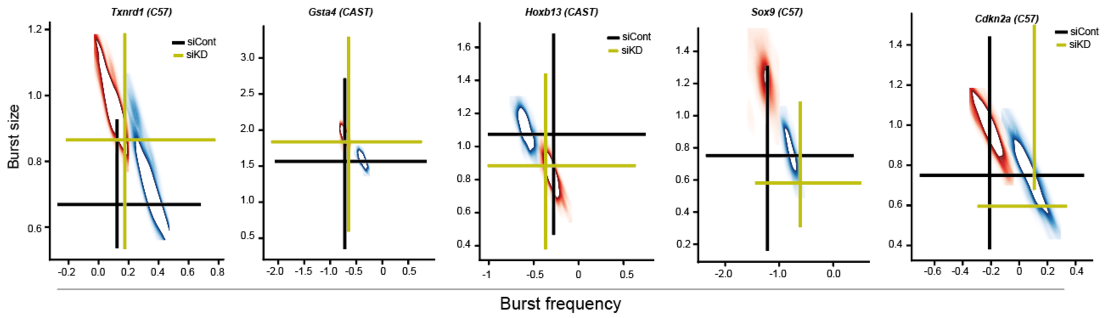

Reviewers Figure 13. Bursting inference and simulation for the alternative alleles.

Regarding the confidence intervals. Although the visual inspection of overlap of confidence intervals is often used to gauge for significance, it is well established that it is a convenient, yet conservative, approach (e.g. in these two papers: Cumming G, Finch S. Inference by eye: confidence intervals and how to read pictures of data. Am Psychol. 2005 Feb-Mar;60(2):170-80. doi: 10.1037/0003-066X.60.2.170. PMID: 15740449, and https://www.tandfonline.com/doi/abs/10.1198/000313001317097960). To quote the conclusion from the latter study above:

"The overlap method is simple, and it is convenient when lists or graphs of confidence intervals are presented. It can be useful as a quick and relatively rough method for exploratory data analysis. It should not be regarded as an optimal method for significance testing, however, given its conservatism and low power relative to the standard method in the common situation that we have considered. Thus, the overlap method should not be used for formal significance testing unless the data analyst is aware of its deficiencies and unless the information needed to carry out a more appropriate procedure is unavailable."

For Figure 6L,M, we apologize for not properly describing the nature of the statistical tests performed and the simulations. For hypothesis testing for changes in burst kinetics we use the likelihood-ratio test. The test-statistic for this test is essentially the difference between the likelihood of the null hypothesis (no change) and the likelihood of the observed change. Expressed as a formula

$$\lambda_{LR} = -2[l(\theta_0) - l(\hat{\theta})]$$

Where λ_{LR} is the likelihood-ratio test statistic, $l(\theta_0)$ is the maximal log-likelihood where the null hypothesis is true, and $l(\hat{\theta})$ is the log-likelihood of the maximized likelihood function (i.e observed change). By Wilk's theorem, λ_{LR} converges asymptotically to the χ^2-distribution under the null hypothesis. This enables hypothesis testing of burst kinetics by comparing λ_{LR} to the χ^2-distribution with one degree of freedom. At $\alpha = 0.05$, the critical value is 3.84 for a one-sided test and 7.68 for a two-sided test.

In the context of burst kinetics, we focus on the log-ratio between e.g. burst frequency in the two samples. I.e. we set the null hypothesis $\theta_0 = 0$ and the alternative hypothesis $\hat{\theta} = \log_2 \frac{k_{on_2}}{k_{on_1}}$ where k_{on_1} and k_{on_2} are the maximum likelihood estimates for both samples respectively.

Regarding the simulations, here we again apologize for its cryptic presentation. Our goal was to estimate the spread in inferred kinetics parameter values, given that the observed changes in expression were only caused by changed burst frequency or size, respectively. To evaluate the spread of changed burst frequency, we first modified the burst frequency by the observed change in mean RNA expression (assuming it is 100% explained by frequency) and then we simulated RNA count observations from the beta-Poisson model (i.e. the two-state model) with the same amount of cells as present in the experiment. Then we infer the kinetic parameters and the densities of inferred parameters were shown as clouds in the "burst kinetics parameter space". The rationale is that an alteration exclusively caused by any of the parameters would

be expected to occur in these subsets of space, to guide the intuition and further support the hypothesis testing performed above.

In the revised manuscript, we have added a proper methods description that carefully describes these important steps (on page 36-37), and we thank the reviewer for bringing this to our attention.

Other issues:

-Figure 1H Y axis label: typo: "permutaions".

The typo has been fixed.

-Page 6: "against 100 expression matched mRNAs" (delete 's')

The grammar has been fixed.

-Throughout: For exponents use base 10: i.e. "<1x10-4" not "<1e-4". This causes confusion, especially in Figure 6C where "e" could be confused as Euler's number.

The base has been changed throughout the paper.

-Figure 2H/I Y axis label: "Occurrence".

The typo has been fixed.

-p7 "possibly regulated by enhancer activities[20,28–30]" is cryptic and needs clarification.

We agree. This statement was not very clear and has been removed. We now briefly speculate how enhancer activities might regulate transcriptional burst frequencies (of lncRNAs) in the Discussion. On page 19, first paragraph, we now write (new text is underlined):

*"While the lowered burst frequency of lncRNAs (four-fold decrease, **Figures 2D** and **S4A**) likely represent a decrease in enhancer-mediated transcriptional initiation, the more modest effect on burst size (two-fold decrease, **Figures 2E and S4B**) could relate to differences in promoter features."*

-p8 Explain more clearly that the "50 most variable lncRNAs on each allele (ranked CV2)" were chosen also according to mean expression.

We agree with the reviewer that this selection was not very well motivated. A lower threshold for mean expression was mainly needed since there are few mRNAs that can be used to match the expression of lncRNAs. It was also considered that burst inference tends to be less sensitive for low abundant genes. We now write (new text is underlined):

*"Having determined that lncRNAs on average show increased cell-to-cell variability compared to expression matched mRNAs (**Figures 1D-G** and **S2A-B**), we next explored if this observation was associated with a systematic pattern of bursting parameters. To this end, we focused specifically on a subset of lncRNAs with high variability on each allele (ranked CV^2, top 50 lncRNAs) (**Figures S4H-I**), excluded genes with mean expression levels below 0.1 UMI (since there were too few genes that could be expression matched in this region) and generated thousands of sets of randomly drawn expression-matched mRNAs (similar as in **Figure 1F**)."*

-p9 Figure S4M: in the main text explain that the "increase in expression" was approximately 50%. Similarly (Figure 2L) explain that the burst size increase is ~ +50%.
To clarify, we added the fold changes related to Figure 2K (UMI, not allele resolved) to the figure legend. We now write:
"$Fold_{coding\text{-}coding}$: 5.44, $Fold_{coding\text{-}lncRNA}$: 5.37, median"
In the main text, we further specify by writing:
*"(**Figure 2K**; <u>approximately 5-fold increase)</u>."*

<u>Related to Figure S4M.</u> We have specified the increase in expression in the figure legend (we did not find this easy to read as part of the main text). We now write:
"$C57_{coding\text{-}coding}$ = 1.31, $C57_{coding\text{-}lncRNA}$ = 1.27, $CAST_{coding\text{-}coding}$ = 1.27, $CAST_{coding\text{-}lncRNA}$ = 1.29, fold changes, median"
<u>Related to Figure 2L:</u> We do not fully understand this comment. There is no/very minor change is burst size, here exemplified by the C57 allele:
-Fold difference of burst size, intergenic / divergent (coding-coding): 1.05
-Fold difference of burst size, intergenic / divergent (coding-lncRNA): 1.01

-p9 "Asynchronously growing mouse fibroblasts (Figures S1A-C)" – is this Figure S5A-C, instead?
Figures S1A-C represent the quality control for cells in figures S5A-C. We have clarifeid this by adding the number of cells (n=533) on page 9.

-p12 "This approach strongly reduced the number of candidate genes" – please provide further information regarding q-value thresholds for the Spearman pairwise correlations.
The q-value (+/- 0.1) has been added to figure 4C.

-Figure 4C: Aside from previous data in the literature, why were 3 kinesins cherry-picked from among 138 genes? Are the other 135 genes also involved in mitosis, or not?
The 3 kinesins were all ranked among the top 15 genes (raking 3, 12, 15 based on positive spearman correlation, genes also required to be significant). The literature also suggests Kif11 and Kif14 to be directly regulated by p27 (the protein coding gene in closest proximity to *Lockd*). Based on these collective features and observations, the kinesins were selected for further investigations. To clarify our selection criteria, we now specify that all 3 kinesins were among the top 15 ranked genes (on page 12). Several other genes related to cell cycle progression are also identified, i.e. *Mki67*, *Smc4* (involved in chromosome condensation), *Prc1* (associated with the mitotic spindle during anaphase). The complete list of genes passing our criteria is now attached as Supplementary Table 7.

-p12 What was the specific rationale for investigating Wincr1 further?
We found it interesting that there is a lncRNA at this locus has been discovered to regulate transcription of the p16 gene locus in human cells (the lncRNA-MIR31HG, Montes M et al. doi: 10.1038/ncomms7967). *Wincr1* is not annotated in the human genome but seems to have similar functions as MIR31HG. Since we also found *Wincr1*

to be highly reduced in expression upon serum starvation (**Figure S5G**), we decided to continue with further in-depth characterization. We also refer to our more comprehensive reply to main comment 2 regarding selection of our candidate genes.

-p13 Figure S7B and "coordinated expression". Such "rippling effects" have been known for over 10 years (Ebisuya et al. Nature Cell Biol 10, 1106) and are not necessarily reflective of coordinated effects with cellular consequences.
Coordinated expression on the allelic level has, to our knowledge, not been investigated and evaluated for lncRNA-mediated functions. The 'rippling effect', as discussed by Ebisuya et al, relates to bulk expression levels and whether the rippling effect takes place in individual cells and/or on the same allele, is not addressed. As discussed also under main comment 3, we fully agree that coordinated expression of lncRNAs and protein-coding genes should not be confounded with function. We have rephrased this part to make the distinction between a strategy to identify pairs of potential *cis*-regulatory function from experiments needed to demonstrate such a function.

-p13 Typo: "mechansisms". Figure 4F: the possibility that Wincr1 maintains other trans-acting functions is of interest, but the data would also be consistent with expected off-target effects.
-The typo has been corrected.
- We find it more likely that the effects are specific because, 1) Two siRNAs as well as a stable shRNA transduced cell line show a similar phenotype, 2) The siRNA induced phenotype is dose dependent (in NIH3T3 cells) and scales with the knockdown (**Figure S7A** left panel, and **Figure 4F**). We now better highlight that the degree of knockdown scales with the effect in NIH3T3 cells. The reader is now directed to **Figure S7A** where the siRNA induced knockdown in presented.

-Bottom of p13, state that you used GO for the genes related to apoptotic signaling.
GO 0043065 has been added on page 13.

-Figure 5A: it is good practice to provide the %age of variance explained by each PC.
The variance explained by each PC has been added to **Figure 5A** (PC1 25%, PC2 12.1%).

-p14 Figures 5B and S8B. The "focusing specifically" on a "cluster of cells that expressed genes involved in stress signalling" looks again like cherry-picking, especially when only two genes (Gadd45b and Cdkn1a) are exemplifications of this gene annotation category. At this point, therefore, the reference to "stress signaling" is not merited, and should be removed.
We are not sure if we fully understand the reviewer's comment. We agree that the assignment of cell clusters and cell states is highly dependent on manually curation of gene expression, and more automated processes would be highly desirable. However, using marker genes to annotate cell clusters is a common practice within the scRNA-seq field, and *Ckdn1a* is a very well-studied target upon p53 activation. Upon revision, we have clarified that *Gadd45b* and *Cdkn1a* are two representative genes (among others), and we no longer refer to 'stress signaling'. Attached are also two more

representative genes, *Btg1* and *Ccn1* (**Reviewers Figure 14**). On page 14, second section, we now write:

*"We identified three clusters of cells (**Figure 5A**) and focused specifically on one cluster of cells that expressed genes involved in growth arrest and DNA damage, exemplified by Gadd45b and the p53 target gene Cdkn1a."*

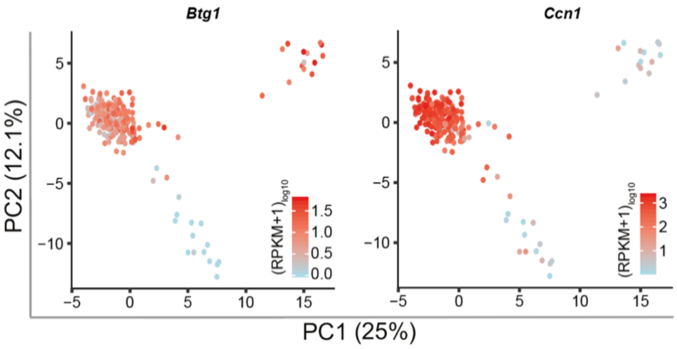

Reviewers Figure 14. Low dimensional PCA projection of cells, based on the most variable genes annotated in Figure 8A. The expression levels of two marker genes are presented.

-Figure 5C: were these the only 5 lncRNAs? If not, then why were these selected? re: "scaled with the concentration of MMC (Figure 5D)" – statistical support (if any) for this statement is required. Why were 3 of 5 lncRNAs chosen, and on what basis?

Statistical support has been added for dose dependent response. Indeed, only two of five candidates have a significant difference when comparing treatments of 2.5µM to 5µM of MMC. This has also been clarified in the text on page 14.

For selecting the 5 candidate lncRNAs we primarily refer to our comprehensive reply regarding the cell cycle annotated lncRNAs (main comment 2). Briefly, we selected candidates based on **1)** p-values, **2)** fold induction, **3)** the possibility of doing downstream functional studies and **4)** literature research. We initially evaluated 4 additional lncRNAs (**Reviewers Figure 15**, pasted below, the data represents a biological duplicate). The selection of candidates worked very well, and we had to make some priorities since we simply could not handle that large number of candidates. At this point, *5830432E09Rik* was excluded from further analysis due to its difficult genomic localization (fully internal and antisense to the protein coding gene *Ptpre*) while we considered *2410017I17Rik* to be expressed at too low levels (Ct value >35 in NIH3T3 cells). We have currently not added this information to the manuscript, but it can be attached as a supplementary note upon the reviewer's request. In the revised manuscript, we have clarified the selection strategy further (on page 15, new text is underlined):

*"Single-cell differential expression (SCDE) was applied to find lncRNAs with increased expression in this cluster of cells and a total of five highly ranked lncRNAs <u>(based on adjusted p-values and fold changes, using a similar selection strategy as for the cell cycle related lncRNAs),</u> towards which siRNAs could be designed, were selected for further validation (**Figure 5C**)."*

Regarding selection of the final three candidates: It was practically undoable to proceed with all lncRNA for AnnexinV staining, using multiple siRNAs and MMC treatments.

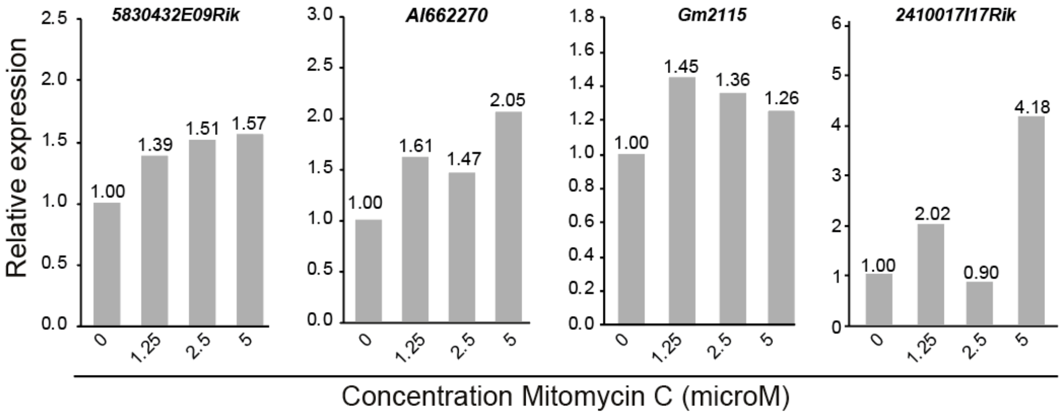

Reviewers Figure 15. qRTPCR measurements of lncRNAs associated with MMC treatment. Bars represent the mean average of 2 replicates.

-p15 Using exactly what criteria were the 4 "highly ranked lncRNA-mRNA interactions" selected? Were siRNAs designed for other targets but are not discussed because of null results?

The candidates were among the lncRNA-mRNA gene pairs with the highest rank (illustrated in **Figure 6F**). We also aimed for 2 gene pairs with a negative allelic score and 2 gene pairs with a positive allelic score. As discussed above under main comment 2, we generally used **1)** p-values, **2)** fold changes (here allelic imbalance), **3)** the possibility of doing downstream functional studies and **4)** literature research for the selection. Here, we also took genomic proximity (mRNA-lncRNA) in consideration (illustrated in **Figure 6E)** and aimed for different lncRNA subgroups (antisense and intergenic lncRNAs (illustrated in **Figure S9G-H**). We have strengthened our criteria by writing (on page 17, new text is underlined):

*"Four highly ranked lncRNA-mRNA interactions, all accessible to siRNA depletion, within 25kb of each other and with diverse genomic organization (**Figure S9G**), were examined more deeply."*

Also note that the 25 kb limit was extracted from **Figures 6 D, H** and **I**, where we illustrate an enrichment of significant lncRNA-mRNA interactions within this distance. Finally, no more lncRNA-mRNA interactions were evaluated in addition to these four.

-p17 Can you please quantify the "good agreement" between the Smart-seq3 and qRTPCR results?

*We agree with the reviewer that this statement was vague. We now specify that we refer to relative fold changes and attach a supplementary table (new **Supplementary Table 11**) showing the individual fold changes of qRTPCR and Smart-seq3 measurements. We now write (new text is underlined):*

*"We first compared fold changes of the Smart-seq3 measurements (**Figures S11G-L**) with those of qRTPCR (**Figures S10A-F**) and found generally good agreement with approximately similar fold changes (**Supplementary Table 10**)."*

-p23 The code for these processing steps should be made freely available.

Code is being prepared and will be made available on GitHub (https://github.com/sandberg-lab).

-Also, it would be of interest to know whether lncRNA burst size/frequency vary by whether the lncRNA has a single or else multiple exons.

We thank the reviewer for this excellent suggestion, and we have now investigated how splicing/exons affect transcriptional bursting. We initially addressed the question by using the burst inference for mRNAs (intergenic / non-divergent genes) and observed some intriguing effects where burst frequencies seems affected, while burst sizes are not (**Reviewers Figure 16A**, pasted below). While we find this of great interest, we did not see a similar trend for lncRNAs (**Reviewers Figure 16B**). Although we currently do not fully understand this observation, we speculate that the relatively few lncRNAs genes with a single exon (and successful burst inference) is not powerful enough to see any effect of splicing / exons (21 lncRNAs and 103 mRNAs with a single exon). To evaluate the lack of power, we randomly downsampled mRNAs to match the number of lncRNAs and simultaneously required the same distribution of exons. Notably, after downsampling the effect on burst frequencies was no longer as prominent (**Reviewers Figure 16C**). At this point, we do not see that this finding fits within this paper and these new data have not been added to the manuscript. We are however looking forward to further evaluate this early observation and we are confident that single-cell protocols with higher sensitivity and data sets covering more cells will help us to do so.

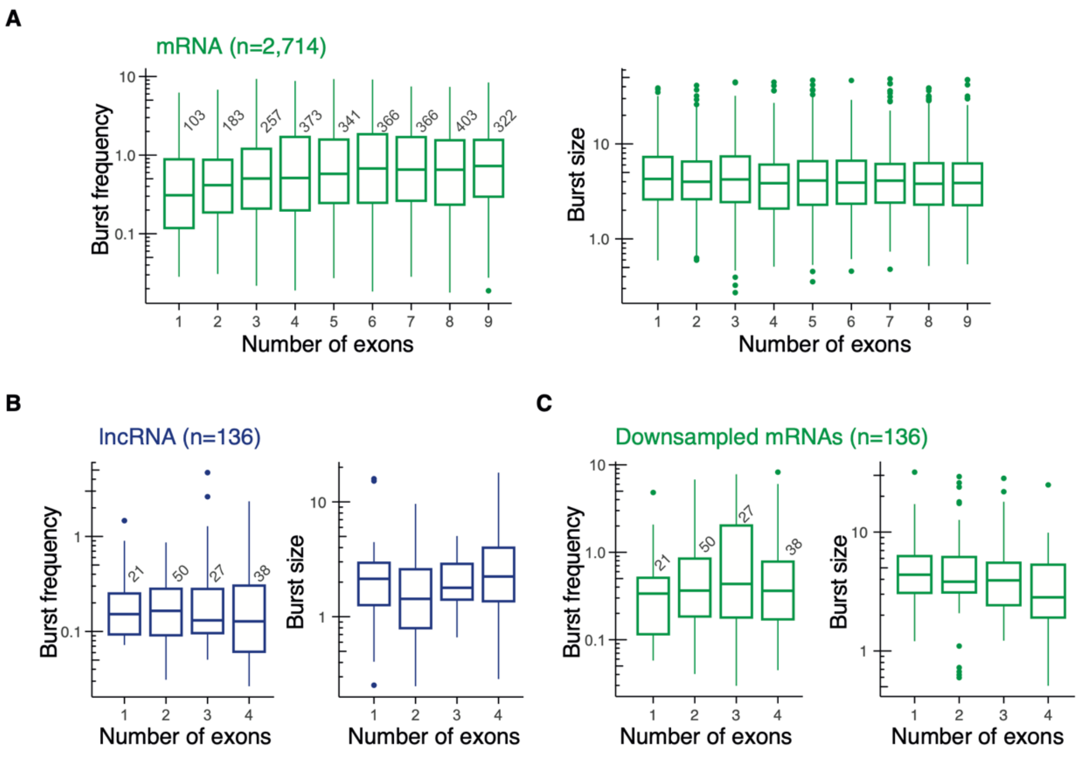

Reviewers Figure 16 (A) Burst frequencies and burst sizes for mRNAs having 1-9 exons. (B) Burst frequencies and burst sizes for lncRNAs having 1-4 exons. **(C)**. Burst frequencies and burst sizes for downsampled mRNAs (equal number genes for each number of exons).

natureresearch

Decision Letter, first revision:

Our ref: NG-A56364R

28th Jul 2021

Dear Rickard,

Thank you for submitting your revised manuscript "Transcriptional kinetics and molecular functions of long non-coding RNAs" (NG-A56364R). It has now been seen by the original referees and their comments are below. The reviewers find that the paper has improved in revision, and therefore we'll be happy in principle to publish it in Nature Genetics, pending minor revisions to satisfy the referees' final requests and to comply with our editorial and formatting guidelines.

If the current version of your manuscript is in a PDF format, please email us a copy of the file in an editable format (Microsoft Word or LaTex)-- we can not proceed with PDFs at this stage.

We are now performing detailed checks on your paper and will send you a checklist detailing our editorial and formatting requirements in about a week. Please do not upload the final materials and make any revisions until you receive this additional information from us.

Thank you again for your interest in Nature Genetics Please do not hesitate to contact me if you have any questions.

Sincerely,

Michael Fletcher, PhD
Associate Editor, Nature Genetics

ORCID: 0000-0003-1589-7087

Reviewer #1 (Remarks to the Author):

The authors have addressed all our comments in a satisfactory and thoughtful way, and this has led to a substantial improvement of the manuscript. I can now recommend publication in Nature Genetics.

Reviewer #2 (Remarks to the Author):

The authors have addressed my one major concern on ASE. I agree that Airn is a great example of validating ASE as well as Xist. I also note that the authors took great consideration and effort to address the other reviewers concerns as well. In short, I think this manuscript will be of great interest to the general nature genetics readership!

Reviewer #3 (Remarks to the Author):

I'd like to thank the authors for their thorough response to my previous comments. I am still of the opinion that the authors' explanation of low burst frequency for lncRNA expression level is an important novel finding.

• Typos: Supplementary Figure S4E. Y-axis "Half".
• Figure 3 legend (and elsewhere) "Student's t-test".
• Throughout including Figures: For exponents use base 10: i.e. "<1x10-4" not "<1e-4". This causes confusion.
• Page 13 Probably no need for "finally" in sentence beginning: "The effect on Kif14 was…"
• New section: "Functional investigation of A730056A06Rik". Some of this would benefit from a more concise rewrite.
• Page 18 "Figures J-K" – insert "6". "single-cell resolution", add "approach".
• Page 19 "found [to be] less efficient…"
• Discussion "we detected a typical lncRNA in only 3% of cells". Because the cDNA conversion efficiency of scRNA-seq is low, then this will not be the same as saying that a lncRNA is typically expressed in 3% of cells. This difference could be made clear.
• Also, it could be clearer that some conclusions drawn are specific only to divergently transcribed lncRNA-mRNA pairs.
• The conclusion (Discussion) that different knockdown strategies have different off-target effects is well known (e.g. Stojic et al. NAR 2018 46, 5950).
• It would be preferable if the code were to be available prior to being requested.
• Page 28 Add word "data" somewhere in sentence starting "Smart-seq2 libraries of mES cells…"
• Figure 1H not necessarily required as one of the main Figures.

Author Rebuttal, first revision:

Remaining reviewer comments

We are again very grateful for the comprehensive and insightful comments from the reviewer. The manuscript has improved greatly with the help from the reviewer.

• Typos: Supplementary Figure S4E. Y-axis “Half”.
Reply: The typo has been fixed.

• Figure 3 legend (and elsewhere) “Student’s t-test”.
Reply: The misspelling has been corrected throughout the manuscript.

• Throughout including Figures: For exponents use base 10: i.e. “<1x10-4” not “<1e-4”. This causes confusion.
Reply: The exponents have been changed throughout the manuscript.

• Page 13 Probably no need for “finally” in sentence beginning: “The effect on Kif14 was…”
Reply: ’Finally’ has been removed.

• New section: “Functional investigation of A730056A06Rik”. Some of this would benefit from a more concise rewrite.
Reply: This section has been revised.

• Page 18 “Figures J-K” – insert “6”. “single-cell resolution”, add “approach”.
Reply: ’Approach’ has been added.

• Page 19 “found [to be] less efficient…”
Reply: ’To be’ has been added to sentence.

• Discussion “we detected a typical lncRNA in only 3% of cells”. Because the cDNA conversion efficiency of scRNA-seq is low, then this will not be the same as saying that a lncRNA is typically expressed in 3% of cells. This difference could be made clear.
Reply: We refer to ‘detected’ to avoid confusion and discuss the sensitivity of current single-cell RNA-seq protocols throughout the manuscript.

• Also, it could be clearer that some conclusions drawn are specific only to divergently transcribed lncRNA-mRNA pairs.
Reply: Divergently transcribed genes are discussed in a separate section of the Discussion, starting with ‘Interestingly, pairs of genes that are divergently transcribed (lncRNA-mRNA as well as mRNA-mRNA gene pairs)…’

• The conclusion (Discussion) that different knockdown strategies have different off-target effects is well known (e.g. Stojic et al. NAR 2018 46, 5950).
Reply: The reference has been added.

• It would be preferable if the code were to be available prior to being requested.
Reply: R code for data analysis and plotting has been made available at (https://github.com/sandberg-lab/lncRNAs_bursting).
Pre-processed count tables and bursting kinetic parameters have been uploaded and made available.

• Page 28 Add word “data” somewhere in sentence starting “Smart-seq2 libraries of mES cells…”
Reply: ‘Data’ has been added.

• Figure 1H not necessarily required as one of the main Figures.
Reply: Figure 1H has been moved to Extended Data Figure 2c.

Final Decision Letter:

In reply please quote: NG-A56364R1 Sandberg

5th Jan 2022

Dear Rickard,

I am delighted to say that your manuscript "Transcriptional kinetics and molecular functions of long non-coding RNAs" has been accepted for publication in an upcoming issue of Nature Genetics.

Over the next few weeks, your paper will be copyedited to ensure that it conforms to Nature Genetics style. Once your paper is typeset, you will receive an email with a link to choose the appropriate publishing options for your paper and our Author Services team will be in touch regarding any additional information that may be required.

After the grant of rights is completed, you will receive a link to your electronic proof via email with a request to make any corrections within 48 hours. If, when you receive your proof, you cannot meet this deadline, please inform us at rjsproduction@springernature.com immediately.

You will not receive your proofs until the publishing agreement has been received through our system.

Due to the importance of these deadlines, we ask that you please let us know now whether you will be difficult to contact over the next month. If this is the case, we ask you provide us with the contact information (email, phone and fax) of someone who will be able to check the proofs on your behalf, and who will be available to address any last-minute problems.

Your paper will be published online after we receive your corrections and will appear in print in the next available issue. You can find out your date of online publication by contacting the Nature Press Office (press@nature.com) after sending your e-proof corrections. Now is the time to inform your Public Relations or Press Office about your paper, as they might be interested in promoting its publication. This will allow them time to prepare an accurate and satisfactory press release. Include your manuscript tracking number (NG-A56364R1) and the name of the journal, which they will need when they contact our Press Office.

Before your paper is published online, we shall be distributing a press release to news organizations worldwide, which may very well include details of your work. We are happy for your institution or funding agency to prepare its own press release, but it must mention the embargo date and Nature Genetics. Our Press Office may contact you closer to the time of publication, but if you or your Press Office have any enquiries in the meantime, please contact press@nature.com.

Acceptance is conditional on the data in the manuscript not being published elsewhere, or announced in the print or electronic media, until the embargo/publication date. These restrictions are not intended to deter you from presenting your data at academic meetings and conferences, but any enquiries from the media about papers not yet scheduled for publication should be referred to us.

Please note that <i>Nature Genetics</i> is a Transformative Journal (TJ). Authors may publish their research with us through the traditional subscription access route or make their paper immediately open access through payment of an article-processing charge (APC). Authors will not be required to make a final decision about access to their article until it has been accepted. Find out more about Transformative Journals

<B>Authors may need to take specific actions to achieve compliance with funder and institutional open access mandates. For submissions from January 2021, if your research is supported by a funder that requires immediate open access (e.g. according to Plan S principles) then you should select the gold OA route, and we will direct you to the compliant route where possible. For authors selecting the subscription publication route our standard licensing terms will need to be accepted, including our self-archiving policies. Those standard licensing terms will supersede any other terms that the author or any third party may assert apply to any version of the manuscript.

Please note that Nature Research offers an immediate open access option only for papers that were first submitted after 1 January, 2021.

If you have any questions about our publishing options, costs, Open Access requirements, or our legal forms, please contact ASJournals@springernature.com

If you have posted a preprint on any preprint server, please ensure that the preprint details are updated with a publication reference, including the DOI and a URL to the published version of the article on the journal website.

To assist our authors in disseminating their research to the broader community, our SharedIt initiative provides you with a unique shareable link that will allow anyone (with or without a subscription) to read the published article. Recipients of the link with a subscription will also be able to download and print the PDF.

As soon as your article is published, you will receive an automated email with your shareable link.

You can now use a single sign-on for all your accounts, view the status of all your manuscript submissions and reviews, access usage statistics for your published articles and download a record of your refereeing activity for the Nature journals.

An online order form for reprints of your paper is available at https://www.nature.com/reprints/author-reprints.html. Please let your coauthors and your institutions' public affairs office know that they are also welcome to order reprints by this method.

If you have not already done so, we invite you to upload the step-by-step protocols used in this

manuscript to the Protocols Exchange, part of our on-line web resource, natureprotocols.com. If you complete the upload by the time you receive your manuscript proofs, we can insert links in your article that lead directly to the protocol details. Your protocol will be made freely available upon publication of your paper. By participating in natureprotocols.com, you are enabling researchers to more readily reproduce or adapt the methodology you use. Natureprotocols.com is fully searchable, providing your protocols and paper with increased utility and visibility. Please submit your protocol to https://protocolexchange.researchsquare.com/. After entering your nature.com username and password you will need to enter your manuscript number (NG-A56364R1). Further information can be found at https://www.nature.com/nprot/.

Sincerely,

Michael Fletcher, PhD
Associate Editor, Nature Genetics

ORCID: 0000-0003-1589-7087
